# Label-Free Quantitative Proteomics to Explore the Action Mechanism of the Pharmaceutical-Grade *Triticum vulgare* Extract in Speeding Up Keratinocyte Healing

**DOI:** 10.3390/molecules27031108

**Published:** 2022-02-07

**Authors:** Elva Morretta, Antonella D’Agostino, Elisabetta Cassese, Barbara Maglione, Antonello Petrella, Chiara Schiraldi, Maria Chiara Monti

**Affiliations:** 1 Department of Pharmacy, University of Salerno, Via Giovanni Paolo II, 84084 Fisciano, Italy; emorretta@unisa.it (E.M.); apetrella@unisa.it (A.P.); 2 Department of Experimental Medicine, Section of Biotechnology, Medical Histology and Molecular Biology, University of Campania “Luigi Vanvitelli”, 80138 Naples, Italy; antonella.dagostino@unicampania.it (A.D.); eli.cassese@gmail.com (E.C.); chiara.schiraldi@unicampania.it (C.S.); 3 Farmaceutici Damor S.p.A., 80145 Naples, Italy; barbara.maglione@farmadamor.it

**Keywords:** label-free quantitative proteomics, secretome, *Triticum vulgare* aqueous extract, keratinocytes, wound repair

## Abstract

Plant extracts have shown beneficial properties in terms of skin repair, promoting wound healing through a plethora of mechanisms. In particular, the poly-/oligosaccharidic aqueous extract of *Triticum vulgare* (TVE), as well as TVE-based products, shows interesting biological assets, hastening wound repair. Indeed, TVE acts in the treatment of tissue regeneration mainly on decubitus and venous leg ulcers. Moreover, on scratched monolayers, TVE prompts HaCat cell migration, correctly modulating the expression of metalloproteases toward a physiological matrix remodeling. Here, using the same HaCat-based in vitro scratch model, the TVE effect has been investigated thanks to an LFQ proteomic analysis of HaCat secretomes and immunoblotting. Indeed, the unbiased TVE effect on secreted proteins has not yet been fully understood, and it could be helpful to obtain a comprehensive picture of its bio-pharmacological profile. It has emerged that TVE treatment induces significant up-regulation of several proteins in the secretome (153 to be exact) whereas only a few were down-regulated (72 to be exact). Interestingly, many of the up-regulated proteins are implicated in promoting wound-healing-related processes, such as modulating cell–cell interaction and communication, cell proliferation and differentiation, and prompting cell adhesion and migration.

## 1. Introduction

The proteome is well defined as the set of proteins expressed by an organism. It varies from cell to cell, and it is also altered over time and across different cellular states, for instance, after a specific treatment. Nowadays, total cellular proteome can be accurately measured by proteomics-based mass spectrometry (MS); moreover, in several cases, it seems relevant to focus on selected smaller sets of proteins of interest (the so-called POIs). POIs could be heterogeneous, comprising, for instance, those belonging to a chemically modified subset, to a newly synthesized one, to a pool of membrane proteins, or to a secreted subset of the total proteome. Indeed, the analysis of a more limited proteome fraction provides the advantage that it enables one to identify and quantify those proteins that may be in low abundance in respect to the background of the full proteome [1]. In the research described in this manuscript, which is focalized on the effect of a plant extract on wound healing, it would be straightforward to focus on the secretome, which represents all the proteins that are secreted outside the cell membrane into the extracellular matrix [2,3]. The secretome encompasses a significant group of proteins, expected to be around 13–20% of the entire proteome and regulating a plethora of biological and physiological processes, as a relevant supply for therapeutic target detection [4]. Secreted proteins include cytokines, coagulation factors, growth factors, cell–cell adhesion, and other signaling molecules; the secretome is easy to collect and, using opportune precipitation methods, it is possible to prepare samples with an appropriate protein concentration for proteomic analysis.

**Figure 1:**
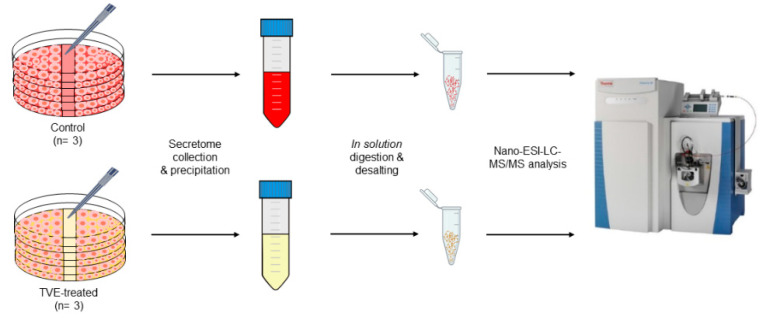
Experimental workflow.

In this paper, label-free quantified (LFQ) secreted proteins were measured by nano-liquid chromatography coupled to tandem mass spectrometry (nLC–MS/MS; Figure 1) to study the alterations in HaCat cell secretomes upon scratching induced by an aqueous extract of *Triticum vulgare* (TVE) compared to the same sample with scratching induced without aqueous TVE.

Indeed, plant extracts have shown beneficial properties in terms of skin repair, promoting wound healing through a plethora of mechanisms [5]. Wound healing is a multifaceted process, and it is balanced by several features, such as the level of damage; it is often linked with the concomitant inflammation process, making wound management a tricky matter. Recently, the development of agents speeding tissue wound repair has been of interest, the aim being to reduce the therapeutic process [6]. In this scenario, pure bioactive phytochemical constituents of plants, such as alkaloids, flavonoids, tannins, saponins and phenols, essential oils, and extracts, have been tested as wound healing promoting agents due to their pharmacological anti-inflammatory, antioxidant, and antibacterial potential and their potential as inducers of pro-collagen synthesis [5]. Among these, the poly-/oligosaccharidic aqueous TVE as well as TVE-based products showed interesting biological features, prompting scratch repair through the modulation of specific biomarkers, in particular on simple model cells such as HaCat monolayers. However, a detailed and unbiased study on the up- and down-regulation of the secreted proteins has never been reported to the best of our knowledge. TVE acts in the treatment of tissue regeneration mainly on decubitus and venous leg ulcers, sores, and burns [7,8,9]. The recent literature provides more information on the positive effect of TVE on the HaCat cellular model: it has been demonstrated that on scratched HaCat monolayers, TVE prompted cell migration, correctly modulating the expression of metalloproteases toward physiological matrix remodeling. Furthermore, TVE up-regulated, in the short time, the expression of several biomarkers, such as MMP-2, MMP-9, collagen I, elastin, integrin αV, and aquaporin 3, thus resulting in the optimal remodeling of dermal tissue during healing [10]. These pieces of evidence were gained using time-lapse video microscopy (TLVM), gene expression by RT-PCR, and protein quantification by immunoblotting analyses on the whole cells and cell extracts [10].

The present work proceeds from the previous paper by Prof. Schiraldi’s group [10], using the same HaCat-monolayer-based in vitro model to deepen the TVE effect, thanks to LFQ proteomic analysis of the secretome, in order to shed light on the TVE action mechanism as an active principle in pharmaceutical preparations for wound treatment (Figure 1). Indeed, as shown by D’Agostino et al. [10], the most interesting proteins whose expression has been modulated by TVE extract are either secreted, or present on the cell surface, or reside in the extracellular space. Thus, a secretome analysis to unveil novel targets responsible for TVE biological outcome has been favored. Indeed, to the best of our knowledge, TVE effect on secreted proteins has not yet been fully analyzed and it could be helpful for a comprehensive picture of its bio-pharmacological profile.

We have demonstrated that TVE treatment, in respect of the control, induces the up-regulation of several proteins in the secretome (153) whereas only a few are down-regulated (72). Interestingly, many of the up-regulated proteins are implicated in promoting wound-healing-related processes, such as modulating cell–cell interaction and communication, modulating cell proliferation and differentiation, and prompting cell adhesion and migration.

## 2. Results

### 2.1. Evaluation of the TVE Effect in the Keratinocytes Monolayer Scratch Assay

**Figure 2:**
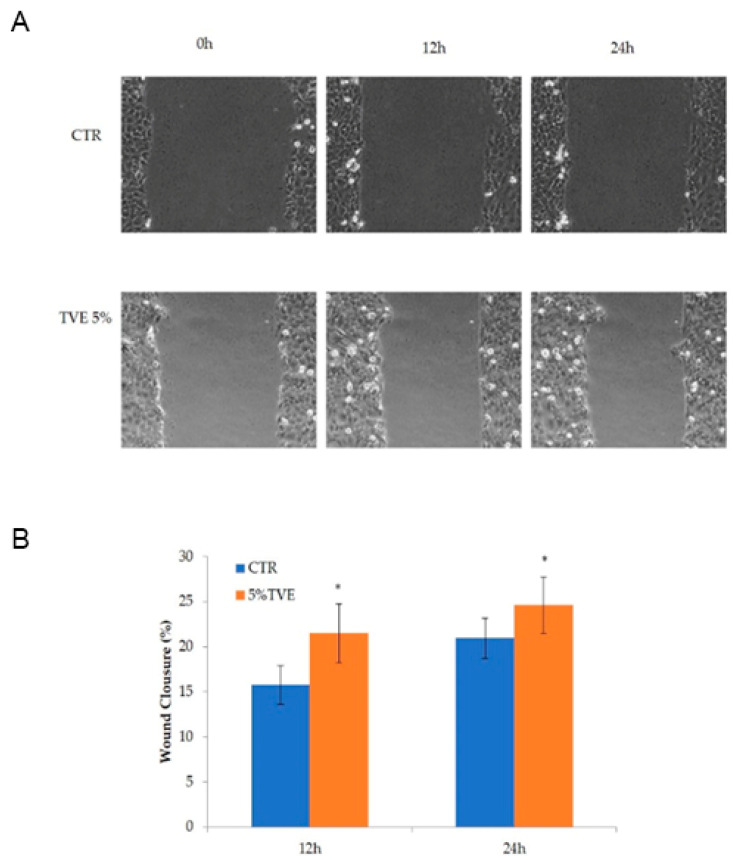
(**A**) Representative panel of scratched cell monolayers during incubation in the control and in the presence of TVE at 5% *v/v*. (**B**) Quantitative analysis of wound closure at 12 and 24 h. Six fields of view were measured for each sample. * *p* < 0.05.

This experiment was accomplished using starved cells, scratched and observed during healing through time-lapse video microscopy. The migration was evident in the first 24 h, and quantitative image analyses showed a significant increase in reparation in the presence of TVE at 5% *v/v*, in comparison to the control. The data are presented in Figure 2.

### 2.2. Proteome Profiling of HaCat Secretomes

**Figure 3:**
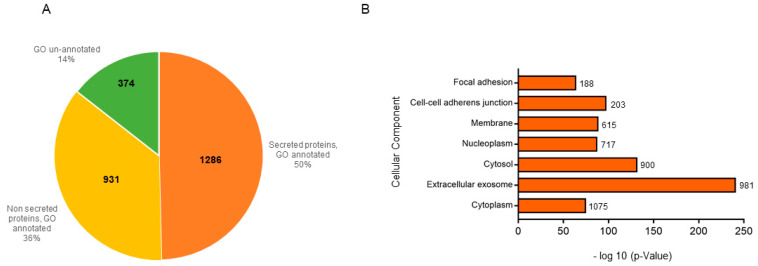
(**A**) Pie-chart of PD cellular component annotation of proteins identified in two secretome samples. (**B**) GO annotation of the cellular component terms of all the identified proteins vs. their *p*-values, performed using the DAVID database and the full *Homo sapiens* proteome as a background. For each term, the corresponding protein count is reported next to the bar.

First of all, to identify HaCat secretome proteins, the samples (n = 2) were precipitated with trichloroacetic acid and re-dissolved in a small volume of a denaturing buffer to achieve the optimal concentration for an in solution digestion. The samples were then analyzed by liquid chromatography–tandem mass spectrometry (nanoLC-MS/MS) on a high-resolution mass spectrometer (Q-Exactive Classic) and protein identification was achieved through the Proteome Discoverer software (PD). For more details, MSPepSearch was used to perform a spectral library search and, subsequently, MS/MS spectra were searched by Sequest against a reviewed *Homo sapiens* database. In total, we identified 2591 proteins common to the two analyzed samples. Then, to assess the quality of our dataset, also gaining functional insights into the identified proteins, we evaluated PD-performed annotation for their cellular component, reflecting protein localization in the cell. As can be observed in Figure 3A, 50% of the PD-identified proteins were annotated as being secretome ones, 36% were annotated as non-secreted ones, and around 14% were un-annotated.

Subsequently, for a deeper analysis of our dataset and to validate PD-obtained results, we performed a Gene Ontology (GO) enrichment analysis through the DAVID database [11,12]. Briefly, this is a system for hierarchically classifying genes or proteins in a graph structure (i.e., an ontology), retrieving, in a ranked list, both the set of terms used to describe these proteins and the corresponding *p*-values. We focalized our attention on the cellular component terms for a direct comparison with PD. Thus, Figure 3B graph reports the terms for which at least 100 proteins were retrievable (y-axis) vs. their corresponding *p*-values (x-axis). Only terms showing a *p*-value smaller than 10^−5^ were selected as the most confident ones. As can be observed, although the highest count was obtained for proteins annotated as being cytoplasmic ones, the lowest *p*-value was the one related to proteins annotated to reside in extracellular exosomes, key component of secretomes, thus boosting these proteins to be the most significant ones among all.

### 2.3. MS-Based Quantitative Proteomics to Disclose Secretome Content Alteration by TVE

Following this initial validation step, we moved to the identification and the label-free quantification (LFQ) of the secretome proteins upon a scratch assay followed by TVE treatment. Three independent secretome samples deriving from HaCat cells treated or not with TVE were subjected to in solution tryptic digestion followed by high-resolution tandem mass spectrometry (LC-MS/MS) analysis, as previously reported and as depicted in Figure 1. Subsequently, both protein identification (performed as already described) and label-free quantification were achieved through PD, allowing one to obtain a list of proteins with their corresponding TVE vs. control abundance ratios retrieved merging all the replicates. For each ratio, the corresponding *p*-value was also obtained.

**Figure 4:**
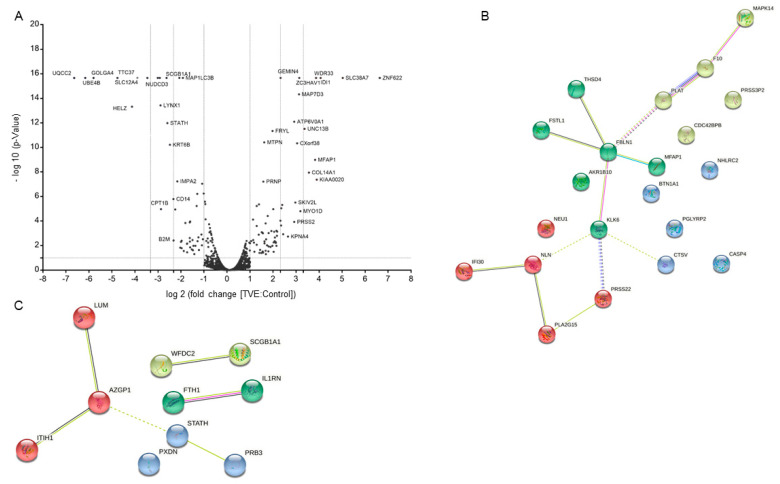
(**A**) Volcano plot of significance vs. fold change of TVE vs. control proteins. STRING analysis of TVE more abundant (**B**) and less abundant (**C**) proteins annotated as being extracellular ones by PD. The four clusters are shown in different colors. Each protein is labeled through its gene name.

In total, we identified 2693 proteins. Of these, 2511 were common between untreated and treated samples (Figure S1) and we focused on high-confidence 2529 proteins, identified by an FDR less than 5%. All of these proteins were also quantified (Supplementary Materials Table S1). Then, the ones quantified in TVE vs. control experiments were compared (Figure 4A) and those proteins with a fold change ≥2 or ≤0.5 (ratio of TVE vs. ctr) and with a *p*-value ≤0.1 in 3 out of 3 experiments were considered as significantly up- and down-regulated. This resulted in 153 significantly up-regulated proteins (i.e., 6.1% of the total) and 72 less abundant ones (i.e., 2.9% of the total). We then focused on the proteins whose Gene Ontology “Cellular Component” annotation reported “extracellular” as a key word (Table 1) and, to cluster them, we exploited the kmeans clustering approach of the web tool STRING [13]. The obtained results are shown in Figure 4B (more abundant proteins) and Figure 4C (less abundant proteins).

### 2.4. Validation through Immunoblotting Analysis

**Figure 5:**
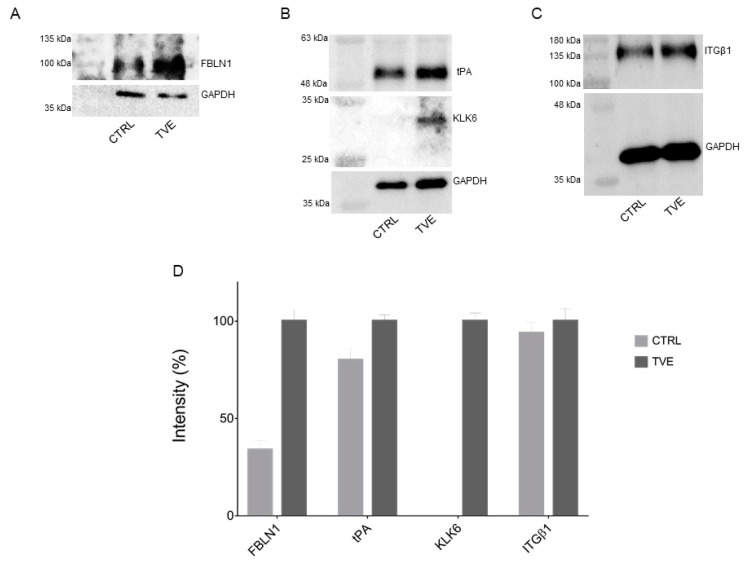
Mass spectrometry data validation through Western blotting for FBLN1 (**A**), tPA and KLK6 (**B**), and ITGβ1 (**C**), each reported with their respective GAPDH for loading normalization purposes. (**D**) Densitometric analyses of panel A, B, and C Western blots. GAPDH was used as a loading normalizer, and TVE intensities were set as 100%.

Primary antibodies specific to fibulin 1 (FBLN1), tissue plasminogen A (tPA), kallikrein 6 (KLK6), and integrin β1 (ITGβ1) were used to carry out immunoblotting experiments for the validation of the proteomic analysis, using GAPDH as a loading normalizer. As shown in Figure 5A–C and in the densitometric analysis in Figure 5D, the over-expression of fibulin 1, tissue plasminogen A, and kallikrein 6 induced by TVE was verified and validated. As expected, the signal relative to integrin β1, whose amount did not change according to proteomic experiments, was unaltered (see also Figure S2).

It should be noted that the up-regulation of kallikrein 6 by TVE by a factor of 5 times has been reported according to proteomics analysis. The results of immunoblotting revealed the presence of kallikrein 6 solely in TVE-treated samples, probably due to the low detection edge and the limited efficiency of the used antibody.

## 3. Discussion

Population aging is a major concern in Western countries, often accompanied by an obesity increase. In this condition, diabetic foot ulcers are a major issue, together with other persistent lesions. Thus, research on potential active principles for new pharmaceutical devices still represents an important challenge [14]. In this scenario, the only plant extract registered as a pharmaceutically active ingredient is TVE, by Farmaceutici Damor S.p.A. TVE is composed of a discrete series of malto-oligosaccharides ranging from a few glucose molecules to 40–60 units, with a molecular weight ranging from ca. 1000 to 8000 Da [9,10].

Despite the long tradition of TVE-based products’ use, the full characterization of an in vitro model treated with TVE and based on the proteomic technologies has never been reported. Among natural products, in fact, many plant-derived polysaccharides may play a valuable role in skin repair although their mechanism of action is still under study, since they are supposed to be active in a complex process involving lots of pathways. In particular, as reported by Romanelli et al. [8], TVE improves the healing of superficial damaged tissues and a reduction in the lesion surface area has been observed; clinical signs (perilesional erythema and bleeding) and symptoms (burning, pain, and itching) of venous leg ulcers were improved using the extract. Moreover, TVE has been tested on HaCat-supported cell proliferation, correctly modulating the timing of metalloproteases expression toward a consistent and well-assessed matrix remodeling [10].

In this paper, a quantitative proteomic approach was applied to investigate TVE-treated HaCat cells with a scratch assay. In detail, the secretomes of HaCat cells in the presence and in the absence of TVE were explored to identify and quantify their protein contents. Indeed, in the era of the -omic sciences, a quantitative MS-based approach can be useful to reveal which proteins and, consequently, which pathways were altered by a specific treatment, such as the use of TVE. Our MS-based results were submitted to appropriate software to identify the up and down-regulated proteins, and a discrete number of the altered ones were validated by immunoblotting analysis, strengthening the proteomic approach.

A considerable number of proteins differentially regulated in HaCaT secretomes in the presence of TVE are known to be involved as promoters of cell migration and in cytoskeleton reorganization, two important events that form the basis of the wound healing process.

Among these proteins, the serine/threonine-protein kinase MRCK beta, which has been totally up-regulated by TVE (the TVE vs. ctr ratio is 100), takes prominence. Indeed, MRCKα and MRCKβ (myotonic-dystrophy-kinase-related Cdc42-binding kinases) belong to a subgroup of Rho-GTPase-activated serine/threonine kinases acting on the actomyosin cytoskeleton. Together with their role in myosin light chain (MLC) phosphorylation, MRCK isoforms control cell shape and motility. Moreover, they are involved in modulating lamellar actomyosin retrograde flow, crucial to cell protrusion and migration [15,16]. Interestingly, the positive effects of TVE in supporting the repair of skin lesions has also been revealed by the finding of the overexpression of thrombospondin type-1 domain-containing protein (the TVE vs. ctr ratio is 100); microfibrillar-associated protein 1, named MFAP-1 (the TVE vs. ctr ratio is 14); and fibulin-1 (the TVE vs. ctr ratio is 4.5, also validated by immunoblotting), all playing a role in cell adhesion and migration and contributing to the supramolecular organization of extracellular matrix (ECM) architecture. These proteins are extracellular matrix glycoproteins, which take part in microfibril assembly, elastinogenesis, and tissue homeostasis [17]. Indeed, microfibrils are composed of several proteins, including fibrillin, fibulins, emilin, thrombospondin, and MFAPs [18,19], with numerous roles during the building of the elastic fiber network [20,21]. Furthermore, follistatin-related protein 1, which is 4.1 times up-regulated by TVE, is a secreted glycoprotein involved in various physiological processes, such as angiogenesis, regulation of the immune response, and cell proliferation and differentiation. There is still no comprehensive understanding of the spectrum of mechanisms of action of follistatin-like proteins, even though it is reported that they promote endothelial cell survival, migration, and differentiation into network structures in an AKT-dependent manner [22].

TVE has been shown as a natural agent able to provide skin homeostasis. This feature can be further explained through the up-regulation of kallikrein 6 (KLK6). In general, KLKs are serine proteases exhibiting trypsin- or chymotrypsin-like activities and sharing important structural and functional properties and they are essential for upholding the epidermal microenvironment. Most of the members of this family are present in skin and regulate skin desquamation and inflammation. KLK involvement has been proved in skin regeneration and pathology [23]. Focusing on KLK6, Klucky and collaborators showed that its overexpression in human keratinocytes increases proliferation and migration, thanks to mRNA-based assays. The KLK6 molecular mechanism of action involves also E-cadherin: in fact, KLK6 up-regulation endorses E-cadherin removal, facilitating keratinocyte migration to wrap the site of injury. In line with this, the overexpression of KLK6 in mice notably diminishes cell-membrane-anchored E-cadherin [24].

During wound healing, the re-epithelialization phase generally begins with the keratinocyte’s migration. Next, these cells trigger the differentiation program, moving from the basal layer toward the outer surface of the skin [25]. Since these events are notably required for a correct sequence of the phases of tissue repair, we have focused on cathepsin L2, also well known as cathepsin V, which has been found to be fully up-regulated in TVE-treated samples, in line with its role in the regulation of keratinocyte differentiation. Indeed, cathepsin L2, which is a cysteinyl protease, is deputed to the assembly of collagen fibrils and other multimeric structures involved in skin homeostasis maintenance [26]. To make possible keratinocyte relocation upon wounding, cells must separate from each other to move into the wounded region, demanding the remodeling of ECM by its limited proteolytic degradation and by de novo biosynthesis of ECM machinery [27,28,29,30,31,32]. In the past, ECM degradation, which in turn promotes cell migration, was thought to be exclusively carried out by serine proteases and by matrix metalloproteinases (MMPs) [28]; several studies have shown that cysteine cathepsins are involved in the partial hydrolysis of ECM constituents in the extracellular space [30,33,34]. Indeed, the discovery that cathepsins are secreted and remain functionally active outside of the lysosome has indicated new roles of these proteins [35], including wound healing [36], bone remodeling, and processing of pro-hormones.

Strictly related to this protein up-regulation, an increase in the expression of γ-interferon-inducible lysosomal thiol reductase (GILT) has been found. Indeed, cathepsins need to be kept in their reduced-active state in the oxidative environment. GILT has been the only enzyme described in the endosomes, lysosomes, and phagosomes with the potential to catalyze a reduction in cysteine cathepsins [37].

One of the most interesting proteins, one that has been 2.3 times up-regulated by TVE, is tissue-type plasminogen activator (tPA), a key factor in the cellular component movement. Its main function is to switch the abundant, but inactive, zymogen plasminogen to plasmin by hydrolyzing an Arg–Val bond. By controlling this proteolytic step, it plays an important role in tissue remodeling and degradation, in cell migration, and in many other physio-pathological events. It has been reported that the serine proteases urokinase plasminogen activator and tissue-type plasminogen activator (uPA/tPA), together with the inhibitor PA-1, are finely regulated in several cell types during injury repair [38].

Taken together, these data support the knowledge that a balanced expression of proteolytic enzymes and their inhibitors is necessary in the wound bed to enable good control of focal proteolysis, to facilitate matrix restructuring and cell motility in complex environments [39].

Regarding the protein caspase 4, which is greatly up-regulated by TVE, it has to be said that this protein is commonly known for its apoptotic function even though, recently, the nonlethal functions of this protein in diverse developmental processes, such as cell differentiation and tissue remodeling, have been disclosed. In particular, a phenomenon termed apoptosis-induced compensatory proliferation has been investigated, suggesting the caspase action to be important in wound healing and tissue regeneration [40].

Concerning the down-regulated proteins, lumican is a dermatan sulfate proteoglycan highly expressed in connective tissue and has the ability to regulate collagen fibril assembly. Previous studies have shown that lumican is involved in wound healing, but the precise effects of lumican on re-epithelialization and wound contraction have not yet been fully understood. For instance, it is reported that lumican can promote skin wound healing by facilitating wound fibroblast activation and contraction but not by promoting keratinocyte proliferation and migration [41]. In addition, other studies have analyzed the effect of chondroitin variants on keratinocytes response and confirmed that the sulfation pattern has a role in migration proliferation and protein expression and assembly in the ECM. In fact, the sulfation degree of glucosaminoglycans affects different biological processes, such as the interaction with growth factors, cytokines, chemokines, adhesion molecules, and lipo-proteins [42]. Our result is one of the first one obtained from a proteomic study reporting a disregulation of lumican in keratinocyte secretomes. Thus, it appears as an appealing element to deepen the investigation into the role of this protein in keratinocytes and, in particular, in the cross talk between these cells and ECM, during tissue repair.

Another down-regulated protein revealed in this work is ferritin, the major intracellular iron storage protein essential for maintaining the cellular redox status. Interestingly, in recent years, wound healing assays were performed to measure the migration capacity of cells with and without ferritin: it has been reported that the silencing of ferritin expression promotes cellular migration, in full agreement with our data [43]. Finally, the protein called secreted Ly-6/uPAR domain-containing protein 2 (SLURP2) is reported to regulate keratinocytes proliferation, differentiation, and apoptosis. Members of the lymphocyte antigen-6 (Ly6)/urokinase-type plasminogen activator receptor (uPAR) superfamily of proteins are cysteine-rich proteins characterized by a distinct disulfide bridge pattern that creates the three-finger Ly6/uPAR (LU) domain [44]. The SLURP2 is a gene strongly induced in psoriatic skin lesions, but the mechanisms controlling SLURP2 expression are largely unknown, so it still needs to be investigated.

## 4. Materials and Methods

The specific extract of *Triticum vulgare* (TVE) is manufactured by a recently implemented extraction process (U.S. Patent No. 9,895,392) of Farmaceutici Damor (Naples, Italy), and it is used as the active principle in certain pharmaceutical products already marketed in Italy and abroad under the brand name Fitostimoline^®^. As reported by Sanguigno et al. [9] and D’Agostino et al. [10], it is composed by a discrete series of malto-oligosaccharides ranging from a few glucose molecules to 40–60 units, with a molecular weight ranging from ca. 1000 to 8000 Da, as demonstrated by size exclusion chromatographic (SEC)-TDA and mass spectrometry data.

### 4.1. Cell Viability Assay

A human keratinocyte cell line (HaCaT) (purchased from Istituto Zooprofilattico, Brescia, Italy) was cultured in Dulbecco’s Modified Eagle Medium (DMEM) supplemented with 10% (*v/v*) heat-inactivated fetal bovine serum (FBS), 100 U/mL of penicillin, and 100 μg/mL of streptomycin. All materials were purchased from Gibco (MMbiotech, Naples, Italy). The cells were grown on tissue culture plates (Corning Incorporated, New York, NY, USA), using an incubator with a humidified atmosphere (95% air/5% CO_2_ *v/v*) at 37 °C. Collagen was purchased from Sigma Aldrich (Milan, Italy). For the experiment, 5 × 10^4^ cells/cm^2^ were seeded in 6 culture plates (Corning, 100 mm diameter), previously coated with collagen 0.1 mg/mL in CH_3_COOH (2% *v/v*), with immortalized keratinocytes (HaCaT) until complete confluence after 48 h.

### 4.2. Evaluation of TVE Effect on the Keratinocytes Monolayer Scratch Assay

HaCaT cells were seeded at a density of 2 × 10^5^ cells/well in a 12-well plate coated with collagen using DMEM containing 10% *w/v* FBS to obtain a monolayer covering 90–100% of the well area in 24 h [10]. Then, the medium was removed and the monolayers were scratched using a sterile tip as previously described [10]. Then, after rinsing with sterile PBS 3 times, media depleted of FBS was added, to resemble the starvation conditions used in flask cultures for proteomic analyses. Three wells were added with TVE 5% *v/v*, while no additional ingredient was present in control wells. The multiwell was placed on a stage incubator (OKOlab S.r.L., Naples, Italy), and the experiment was followed by the time-lapse video microscopy station for 24 h.

### 4.3. HaCat Secretome Sample Preparation for Proteomics Analysis

Both the preliminary and the TVE experiments were performed in the same way. Thus, HaCat secretomes (preliminary experiment, two biological replicates), HaCat control, and TVE secretomes (three biological replicates) were precipitated with trichloroacetic acid (TCA, final concentration 25% *v/v* in 12 mL) overnight at 4 °C under continuous shaking. Subsequently, the samples were centrifuged (21,000 rcf, 30 min at 4 °C) and the supernatants were discarded. The pellets were then washed with 1 mL of ice-cooled acetone and centrifuged. These operations were repeated twice. The obtained pellets were re-suspended in 80 μL of 8 M Urea/50 mM AmBic (pH 8.5). The protein concentration was evaluated through the Bradford assay (Bio-Rad, Hercules, CA, USA), and then equal protein amounts (50 μg in 60 μL) were submitted to in solution digestion. Briefly, the proteins were reduced with 10 mM 1,4-dithiothreitol (DTT, 1 h, 25 °C, 800 rpm) and the formed thiols were carboxyamidomethylated with 20 mM iodoacetamide (IAA, 30 min, 25 °C, 800 rpm, in the dark), whose excess was then quenched with 10 mM DTT (10 min, 25 °C, 800 rpm). Urea was then diluted to 1 M with 50 mM AmBic, and a trypsin/LysC solution (Promega, Madison, WI, USA) was added at the enzyme-to-proteins ratio of 1:100 *w/w* overnight at 37 °C under continuous shaking. The enzymes were then quenched, adding formic acid (FA) to lower the pH to 3, and the peptide mixture was dried under vacuum, dissolved in 5% FA, and equal amounts of aliquots were desalted through C18 ZipTips (ZipTip^®^ Pipette Tips, Merck Millipore Ltd., Cork, Ireland), as directed by the manufacturer. The obtained samples were dried under vacuum and dissolved in 10% FA for the subsequent nano-UPLC-MS/MS analysis.

### 4.4. Proteomics Analysis: Nano-UPLC-MS/MS

HaCat secretomes for the preliminary experiment and subsequently control and TVE secretome digests (1 μg each) were analyzed on an Orbitrap Q-Exactive Classic Mass Spectrometer (ThermoFisher Scientific, Bremen, Germany) coupled to an UltiMate 3000 Ultra-High-Pressure Liquid Chromatography (UPLC) system (ThermoFisher Scientific, Bremen, Germany), equipped with an EASY-Spray PepMAP^TM^ RSLC C18 column at 35 °C (3 μm, 100 Å, 75 μm × 50 cm, ThermoFisher Scientific, Bremen, Germany). Peptide elution was achieved at a flow rate of 300 nL/min with the following gradient: 1 min at 3% B, 1–120 min to 38% B, 120–121 min to 80% B, 121–131 min at 80% B, 131–132 min back to 3% B, until 140 min (A: 0.1% AcOH, 95% H_2_O, and 5% ACN; B: 0.1% AcOH, 95% ACN, and 5% H_2_O). The mass spectrometer was operated in data-dependent acquisition mode. Full-scan MS spectra were acquired with the following settings: scan range 375–1500 m/z, full-scan automatic gain control (AGC) target 3e6 at 70,000 resolution, and maximum injection time 80 ms. MS2 spectra were generated for up to 6 precursors (normalized collision energy of 28%) and the fragment ions acquired at a resolution of 17,500 with an AGC target of 1e5 and a maximum injection time of 110 ms.

### 4.5. Data Processing through Proteome Discoverer

All the raw MS files were searched with the Proteome Discoverer software (version 2.4.1.15). MSPepSearch was used to perform a spectral library search (NIST Human Orbitrap HCD Library, 1127970 spectra, September 2016) with a mass tolerance of 10 ppm for MS1 and 0.02 Da for MS2. The target false discovery rate (FDR) were set to 1% (strict) and 5% (relaxed). Subsequently, MS/MS spectra were also searched by Sequest against a reviewed *Homo sapiens* database (SwissProt, November 2020, 20380 entries) using the following parameters: trypsin digestion, maximum of two missed cleavages, cysteine carbamidomethylation as fixed modification, methionine oxidization, protein N-terminal acetylation, and/or de-methylation as variable modifications. Mass tolerances and FDR were set as previously reported. Label-free quantification was performed exploiting both unique and razor peptides for protein abundance calculation, and a pairwise ratio-based approach was used to evaluate TVE vs. control protein abundance. For each calculated ratio, a background-based *t*-test was performed.

### 4.6. Data Visualization

Proteome-Discoverer-identified proteins were represented through GraphPad Prism 7.0 as a volcano plot, plotting each protein in terms of –log_10_ *p*-value vs. the related log_2_ (TVE/control) fold change. Subsequently, protein clustering was performed through the web tool STRING using a 0.2 confidence and applying a kmeans cluster approach to four clusters.

### 4.7. Immunoblotting Analysis

Aliquots of the same TVE-treated and control samples of the different biological replicates exploited for the MS analysis were pooled together, and 10 μg of protein of each pool was treated with Laemmli buffer (60 mM Tris-HCl pH 6.8, 2% SDS, 0.001% bromophenol blue, 1% glycerol, 2% β-mercaptoethanol) and heated for 5 min at 95 °C. The samples were then loaded into a 12% polyacrylamide gel for monodimensional electrophoresis, and the subsequently resolved proteins were transferred onto a nitrocellulose membrane. The membrane was soaked for 1 h at room temperature in 5% non-fat dried milk containing TBS-t solution (31 mM Tris, pH 8, 170 mM NaCl, 3.35 mM KCl, 0.05% Tween 20) and then incubated overnight at 4 °C with a monoclonal primary anti-body against fibulin 1 (FBLN1, mouse, 1:500 *v/v*, sc-25281, Santa Cruz Biotechnology, Inc., Dallas, TX, USA), tissue plasminogen A (tPA, 1:250 *v/v*, sc-69740, Santa Cruz Biotechnology, Inc., Dallas, TX, USA), kallikrein 6 (KLK6, 1:250 *v/v*, sc-374564, Santa Cruz Biotechnology, Inc., Dallas, TX, USA), and integrin β1 (ITGβ1, 1:1000 *v/v*, 34971, Cell Signaling Technologies, Danvers, Massachusetts), all in milk/TBS-t. After recognition of the primary antibodies from the proper HPR-tagged secondary ones (1:500 *v/v* mouse for FBLN1, tPA, and KLK6 and 1:1500 *v/v* rabbit for ITGβ1), chemiluminescence, developed upon treatment of the membrane with a mixture of luminol and hydrogen peroxide, was detected using an ImageQuant LAS4000 imaging system (GE Healthcare, Waukesha, WI, USA). The densitometric analyses were elaborated through ImageJ. Normalized densitometric values were calculated based on the signals obtained by hybridizing the membranes with a monoclonal antibody against GAPDH (Invitrogen by Thermo Scientific, 1: 1500 *v/v*). Immunoblotting analyses were repeated twice.

## 5. Conclusions

The quantitative proteomics analysis of HaCat secretomes treated or not treated with TVE threw further light on its mechanism of action. In particular, the ability of this plant-derived polysaccharide to induce the biosynthesis or release of specific proteins from keratinocytes makes even more attractive its use in the treatment of skin lesions. In turn, the majority of the secreted proteins found to be altered by TVE have been recognized as effectors in cell cross talk, which plays a central role in the repair of tissue damage and in the regeneration of diverse cell types. Finally, our results pave a fascinating way for new modalities of investigation into the mechanism of action of products commonly used in wound repair or even those that are still a subject of research in this field.

## Figures and Tables

**Table 1:** List of the significantly quantified proteins whose “cellular component” is annotated to be “extracellular”. Each protein is reported with its accession, gene name, TVE vs. control abundance ratio, and the related *p*-value.

Accession	Gene Symbol	Description	Abundance Ratio	*p*-Value
O60218	AKR1B10	Aldo-keto reductase family 1-member B10	100.0000	0.0000
Q9GZN4	PRSS22	Brain-specific serine protease 4	100.0000	0.0000
P49662	CASP4	Caspase-4	100.0000	0.0000
O60911	CTSV	Cathepsin L2	100.0000	0.0000
Q8NBF2	NHLRC2	NHL repeat-containing protein 2	100.0000	0.0000
Q9Y5S2	CDC42BPB	Serine/threonine-protein kinase MRCK beta	100.0000	0.0000
Q6ZMP0	THSD4	Thrombospondin-type-1 domain-containing protein 4	100.0000	0.0000
P55081	MFAP1	Microfibrillar-associated protein 1	14.2020	0.0000
A6XMV8	PRSS2	Protease serine 2 preproprotein	7.5430	0.0001
Q92876	KLK6	Kallikrein-6	5.4410	0.0012
Q96PD5	PGLYRP2	*N*-acetylmuramoyl-l-alanine amidase	4.9880	0.0001
P23142	FBLN1	Fibulin-1	4.5230	0.0007
Q99519	NEU1	Sialidase-1	4.5030	0.0016
Q13410	BTN1A1	Butyrophilin subfamily 1 member A1	4.1290	0.0007
Q12841	FSTL1	Follistatin-related protein 1	4.0930	0.0056
B4E0K5	MAPK14	Mitogen-activated protein kinase	3.1000	0.0629
Q9BYT8	NLN	Neurolysin, mitochondrial	3.0870	0.0066
Q8NCC3	PLA2G15	Phospholipase A2 group XV	2.7950	0.0683
P00742	F10	Coagulation factor X	2.6100	0.0013
P13284	IFI30	Gamma-interferon-inducible lysosomal thiol reductase	2.3500	0.0778
P00750	PLAT	Tissue-type plasminogen activator	2.2870	0.0317
P19827	ITIH1	Inter-alpha-trypsin inhibitor heavy chain H1	0.4820	0.0030
P02794	FTH1	Ferritin heavy chain	0.4730	0.0000
P25311	AZGP1	Zinc-alpha-2-glycoprotein	0.4430	0.0637
P51884	LUM	Lumican	0.4100	0.0000
Q92626	PXDN	Peroxidasin homolog	0.4000	0.0000
Q14508	WFDC2	WAP four-disulfide core domain protein 2	0.3870	0.0104
P18510	IL1RN	Interleukin-1 receptor antagonist protein	0.3000	0.0073
H0YLF3	B2M	Beta-2-microglobulin (Fragment)	0.2000	0.0038
P02808	STATH	Statherin	0.1660	0.0000
P11684	SCGB1A1	Uteroglobin	0.1610	0.0000
P0DP57	SLURP2	Secreted Ly-6/uPAR domain-containing protein 2	0.1350	0.0000
Q04118	PRB3	Basic salivary proline-rich protein 3	0.0100	0.0000

## Data Availability

Not applicable.

## Supplementary Materials

The following are available online: Table S1: list of the 2529 identified and quantified proteins. Figure S1: Venn diagram of unique and common proteins identified in HaCat cell secretomes treated or not with TVE. Figure S2: uncropped western blotting analysis reported in Figure 5.
