# Supplementary material for: Label-Free Quantitative Proteomics to Explore the Action Mechanism of the Pharmaceutical-Grade Triticum vulgare Extract in Speeding Up Keratinocyte Healing
Source: Molecules. 2022 Feb 7;27(3):1108. doi: 10.3390/molecules27031108 (PMC8839156; doi:10.3390/molecules27031108)
Supplement: Supplementary file 1 [file molecules-27-01108-s001.zip › molecules-1516843-supplementary.pdf]

Label-free quantitative proteomics to explore the action mechanism of the pharmaceutical grade *Triticum vulgare* extract in speeding up keratinocytes healing.

Elva Morretta[1], Antonella D'Agostino[2], Elisabetta Cassese[2], Barbara Maglione[3], Antonello Petrella[1], Chiara Schiraldi[2], and Maria Chiara Monti[1,*]

[1]Department of Pharmacy, University of Salerno, Via Giovanni Paolo II, 84084 Fisciano, Salerno, Italy; emorretta@unisa.it, apetrella@unisa.it, mcmonti@unisa.it
[2]Department of Experimental Medicine, Section of Biotechnology, Medical Histology and Molecular Biology, Un iversity of Campania "Luigi Vanvitelli", 80138 Naples, Italy; antonella.dagostino@unicampania.it, eli.cassese@gmail.com, chiara.schiraldi@unicampania.it
[3]Farmaceutici Damor S.p.A., 80145 Naples, Italy, barbara.maglione@farmadamor.it
*Correspondence: mcmonti@unisa.it;

Supp. Table S1. List of the 2529 identified and quantified proteins. Each protein is reported with its UniProt Accession, Gene Symbol, Molecular Weight (i.e., MW, in kDa), sequence coverage (%), number of peptides (i.e., Pep.) and its TVE *vs* ctrl abundance ratio (i.e., Ratio) with the related p-Value (i.e., p-Val.).

Accession	Gene Symbol	MW	Description	Coverage	Pep.	Ratio	p-Val.
S4R3N1	HSPE1-MOB4	29.7	10 kDa heat shock protein, mitochondrial OS	45	14	100.0000	0.0000
V9GYX7	ACTR10	33.3	Actin-related protein 10 (Fragment) OS	4	1	100.0000	0.0000
O60218	AKR1B10	36	Aldo-keto reductase family 1 member B10 OS	4	1	100.0000	0.0000
A0A024R CR6	BAG6	118. 6	BCL2-associated athanogene 6 OS	2	2	100.0000	0.0000
Q9BXK5	BCL2L13	52.7	Bcl-2-like protein 13 OS	5	2	100.0000	0.0000
Q9GZN4	PRSS22	33.7	Brain-specific serine protease 4 OS	2	1	100.0000	0.0000
E7EX44	CALD1	64.1	Caldesmon OS	4	1	100.0000	0.0000
P49662	CASP4	43.2	Caspase-4 OS	2	1	100.0000	0.0000
O60911	CTSL2	37.3	Cathepsin L2 OS	4	1	100.0000	0.0000
P17676	CEBPB	36.1	CCAAT/enhancer-binding protein beta OS	3	1	100.0000	0.0000
Q5ZPR3	CD276	57.2	CD276 antigen OS	5	1	100.0000	0.0000
Q9Y3E7	CHMP3	25.1	Charged multivesicular body protein 3 OS	4	1	100.0000	0.0000
Q8NHS4	CLHC1	67.2	Clathrin heavy chain linker domain-containing protein 1 OS	3	1	100.0000	0.0000
A0A3B3I RQ8	CTPS1	50.4	CTP synthase OS	3	1	100.0000	0.0000
C9J0E4	CSTA	7.1	Cystatin-A OS	19	1	100.0000	0.0000
F8VW96	CSRP2	26.7	Cysteine and glycine-rich protein 2 OS	7	1	100.0000	0.0000
Q01459	CTBS	43.7	Di-N-acetylchitobiase OS	3	1	100.0000	0.0000
R4GN05	DPP7	17	Dipeptidyl peptidase 2 (Fragment) OS	17	2	100.0000	0.0000
O00273	DFFA	36.5	DNA fragmentation factor subunit alpha OS	5	1	100.0000	0.0000
H0YK99	DNAJC17	16.2	DnaJ homolog subfamily C member 17 OS	15	1	100.0000	0.0000
Q9UNI6	DUSP12	37.7	Dual specificity protein phosphatase 12 OS	3	1	100.0000	0.0000
A0A087W T96	ARIH1	14.1	E3 ubiquitin-protein ligase ARIH1 (Fragment) OS	10	1	100.0000	0.0000
K7ENS6	NEDD4L	17.6	E3 ubiquitin-protein ligase NEDD4-like (Fragment) OS	14	1	100.0000	0.0000
Q9C037	TRIM4	57.4	E3 ubiquitin-protein ligase TRIM4 OS	2	1	100.0000	0.0000
M0QY67	ETFB	19.5	Electron transfer flavoprotein subunit beta (Fragment) OS	5	1	100.0000	0.0000
Q14789	GOLGB1	375. 8	Golgin subfamily B member 1 OS	1	2	100.0000	0.0000
Q92917	GPKOW	52.2	G-patch domain and KOW motifs-containing protein OS	3	1	100.0000	0.0000
Q9UBI6	GNG12	8	Guanine nucleotide-binding protein G(I)/G(S)/G(O) subunit gamma-12 OS	46	2	100.0000	0.0000
A0A087W Y35	ITGBL1	48.5	Integrin beta-like protein 1 OS	6	1	100.0000	0.0000
Q8N1N4	KRT78	56.8	Keratin, type II cytoskeletal 78 OS	4	4	100.0000	0.0000
A0A1B0G UA3	KIFBP	74.7	KIF-binding protein OS	5	2	100.0000	0.0000
A0A2U3T ZL8	KIF23	92.6	Kinesin-like protein OS	2	1	100.0000	0.0000
P42167	TMPO	50.6	Lamina-associated polypeptide 2, isoforms beta/gamma OS	38	13	100.0000	0.0000
H0YJ82	ABCD4	18	Lysosomal cobalamin transporter ABCD4 (Fragment) OS	13	1	100.0000	0.0000
Q15777	MPPED2	33.3	Metallophosphoesterase MPPED2 OS	4	1	100.0000	0.0000
P80297	MT1X	6.1	Metallothionein-1X OS	84	4	100.0000	0.0000
A0A0A0 MQX8	MBNL1	43	Muscleblind-like protein 1 OS	6	2	100.0000	0.0000
A0A5H1Z RP4	NELFB	70	Negative elongation factor B OS	4	2	100.0000	0.0000
A0A0A0 MSN9	NELFE	38.1	Negative elongation factor E (Fragment) OS	3	1	100.0000	0.0000
Q92859	NEO1	159. 9	Neogenin OS	0	1	100.0000	0.0000
Q8NBF2	NHLRC2	79.4	NHL repeat-containing protein 2 OS	1	1	100.0000	0.0000
Q9HAB8	PPCS	34	Phosphopantothenate--cysteine ligase OS	3	1	100.0000	0.0000

Q9ULM0	PLEKHH1	151.1	Pleckstrin homology domain-containing family H member 1 OS	1	1	100.0000	0.0000
E9PKN0	PCF11	88.9	Pre-mRNA cleavage complex 2 protein Pcf11 (Fragment) OS	1	1	100.0000	0.0000
Q99622	C 10	13.2	Protein C10 OS	10	1	100.0000	0.0000
E9PH82	FAM98A	34.4	Protein FAM98A OS	4	1	100.0000	0.0000
Q8WZA0	LZIC	21.5	Protein LZIC OS	11	2	100.0000	0.0000
Q5JSZ5	PRRC2B	242.8	Protein PRRC2B OS	1	1	100.0000	0.0000
O60245	PCDH7	116	Protocadherin-7 OS	1	1	100.0000	0.0000
H3BTP8	PSME3IP1	16	PSME3-interacting protein (Fragment) OS	19	2	100.0000	0.0000
Q53FA7	TP53I3	35.5	Quinone oxidoreductase PIG3 OS	6	2	100.0000	0.0000
O43665	RGS10	21.2	Regulator of G-protein signaling 10 OS	9	1	100.0000	0.0000
A0A286YFD6	RRM2	42.9	Ribonucleoside-diphosphate reductase subunit M2 OS	5	1	100.0000	0.0000
A0A0C4DGB1	DIMT1	30.8	rRNA adenine N(6)-methyltransferase OS	4	1	100.0000	0.0000
H3BTL8	SCAPER	11.3	S phase cyclin A-associated protein in the endoplasmic reticulum (Fragment) OS	7	1	100.0000	0.0000
Q99611	SEPHS2	47.3	Selenide, water dikinase 2 OS	2	1	100.0000	0.0000
Q13501	SQSTM1	47.7	Sequestosome-1 OS	10	2	100.0000	0.0000
Q9Y5S2	CDC42BPB	194.2	Serine/threonine-protein kinase MRCK beta OS	1	1	100.0000	0.0000
Q5F2F8	PPP3CB	56	Serine/threonine-protein phosphatase OS	2	1	100.0000	0.0000
Q15427	SF3B4	44.4	Splicing factor 3B subunit 4 OS	3	1	100.0000	0.0000
Q96A49	SYAP1	39.9	Synapse-associated protein 1 OS	8	2	100.0000	0.0000
F8WB30	TOM1	2.8	Target of Myb protein 1 OS	64	1	100.0000	0.0000
Q15554	TERF2	59.6	Telomeric repeat-binding factor 2 OS	2	1	100.0000	0.0000
Q6ZMP0	THSD4	112.4	Thrombospondin type-1 domain-containing protein 4 OS	1	1	100.0000	0.0000
Q9H3D4	TP63	76.7	Tumor protein 63 OS	3	1	100.0000	0.0000
Q4G0F5	VPS26B	39.1	Vacuolar protein sorting-associated protein 26B OS	4	1	100.0000	0.0000
Q969S3	ZNF622	54.2	Zinc finger protein 622 OS	3	1	100.0000	0.0000
Q9NVC3	SLC38A7	49.9	Putative sodium-coupled neutral amino acid transporter 7 OS	2	1	32.6180	0.0000
C9JD53	IDI1	13.6	Isopentenyl-diphosphate Delta-isomerase 1 (Fragment) OS	10	1	16.7670	0.0000
Q15397	PUM3	73.5	Pumilio homolog 3 OS	1	1	14.9200	0.0000
Q9C0J8	WDR33	145.8	pre-mRNA 3' end processing protein WDR33 OS	1	1	14.6390	0.0000
P55081	MFAP1	51.9	Microfibrillar-associated protein 1 OS	6	1	14.2020	0.0000
J3QT83	COL14A1	92.6	Collagen alpha-1(XIV) chain (Fragment) OS	2	1	11.8300	0.0000
A0A1B0GUS7	UNC13B	483.8	Protein unc-13 homolog B OS	0	1	10.3310	0.0000
J3KRL0	MYO1D	49.7	Unconventional myosin-Id OS	3	1	9.0980	0.0000
Q7Z2W4	ZC3HAV1	101.4	Zinc finger CCCH-type antiviral protein 1 OS	4	3	8.7850	0.0000
Q8IWC1	MAP7D3	98.4	MAP7 domain-containing protein 3 OS	2	1	8.7780	0.0000
Q8TB03	CXorf38	36.6	Uncharacterized protein CXorf38 OS	2	1	8.2920	0.0000
Q15477	SKIV2L	137.7	Helicase SKI2W OS	1	1	7.8160	0.0000
K7EN36	ATP6V0A1	22.4	V-type proton ATPase subunit a (Fragment) OS	5	1	7.5880	0.0000
A6XMV8	PRSS2	26.7	Protease serine 2 preproprotein OS	4	1	7.5430	0.0001
O00629	KPNA4	57.9	Importin subunit alpha-3 OS	8	3	6.2950	0.0018
Q92876	KLK6	26.8	Kallikrein-6 OS	8	1	5.4410	0.0012
Q14393	GAS6 AXLLG	74.9	Growth arrest-specific protein 6 OS	5	3	5.2810	0.0000
E7ENN3	SYNE1	964.2	Nesprin-1 OS	0	1	5.1180	0.0000
P04732	MT1E	6	Metallothionein-1E OS	54	3	5.0840	0.0002
I3L2C7	GEMIN4	118.7	Gem-associated protein 4 OS	1	1	5.0430	0.0000

Q96PD5	PGLYRP2	62.2	N-acetylmuramoyl-L-alanine amidase OS	3	1	4.9880	0.0001
P23142	FBLN1	77.2	Fibulin-1 OS	26	11	4.5230	0.0007
Q99519	NEU1	45.4	Sialidase-1 OS	4	1	4.5030	0.0016
Q9BW91	NUDT9	39.1	ADP-ribose pyrophosphatase, mitochondrial OS	3	1	4.4430	0.0105
Q8IYD1	GSPT2	68.8	Eukaryotic peptide chain release factor GTP-binding subunit ERF3B OS	15	6	4.4280	0.0006
A0A494C0W0	PARN	76.3	Poly(A)-specific ribonuclease PARN OS	2	1	4.3610	0.0052
H0YLH3	RABGGTA	49	Protein geranylgeranyltransferase type II OS	3	1	4.3320	0.0060
A6QRJ1	ATP6AP1	24.9	V-type proton ATPase subunit S1 (Fragment) OS	6	1	4.2240	0.0022
O00762	UBE2C	19.6	Ubiquitin-conjugating enzyme E2 C OS	18	3	4.1870	0.0044
Q13410	BTN1A1	58.9	Butyrophilin subfamily 1 member A1 OS	2	1	4.1290	0.0007
Q12841	FSTL1	35	Follistatin-related protein 1 OS	3	1	4.0930	0.0056
Q13310	PABPC4	70.7	Polyadenylate-binding protein 4 OS	21	13	4.0840	0.0324
E9PQP6	FNTA	30.1	Protein farnesyltransferase/geranylgeranyltransferase type-1 subunit alpha (Fragment) OS	10	2	4.0650	0.0566
Q9UNW1	MINPP1	55	Multiple inositol polyphosphate phosphatase 1 OS	6	2	4.0150	0.0000
A0A2C9F2R7	FRYL	192.1	Protein furry homolog-like (Fragment) OS	0	1	3.9460	0.0000
J3QLI2	ZBTB14	25.1	Zinc finger and BTB domain-containing protein 14 (Fragment) OS	3	1	3.9460	0.0000
P04181	OAT	48.5	Ornithine aminotransferase, mitochondrial OS	4	1	3.9150	0.0104
H7C3I5	CRYZL1	26.3	Quinone oxidoreductase-like protein 1 (Fragment) OS	13	2	3.8170	0.0006
P59923	ZNF445	118.9	Zinc finger protein 445 OS	1	1	3.7040	0.0128
H0YMT3	WDR73	20.1	WD repeat-containing protein 73 (Fragment) OS	9	1	3.5150	0.0118
A0A0S2Z4G6	TPM1	32.7	Tropomyosin 1 (Alpha), isoform CRA_o (Fragment) OS	38	13	3.5090	0.0010
P05413	FABP3	14.8	Fatty acid-binding protein, heart OS	7	1	3.4880	0.0292
Q9NX01	TXNL4B	17	Thioredoxin-like protein 4B OS	9	1	3.4220	0.1254
P62993	GRB2	25.2	Growth factor receptor-bound protein 2 OS	17	4	3.4210	0.0000
A0A1B0GTB0	ATP6AP2	33	ATPase H(+)-transporting lysosomal accessory protein 2 (Fragment) OS	7	1	3.4130	0.0794
P06732	CKM	43.1	Creatine kinase M-type OS	3	2	3.3410	0.0096
Q8TAT6	NPLOC4	68.1	Nuclear protein localization protein 4 homolog OS	3	2	3.3240	0.0637
Q9Y3B4	SF3B14; SF3B6	14.6	Splicing factor 3B subunit 6 OS	21	2	3.2850	0.0889
A0A087WY56	AKT1	16.7	RAC-alpha serine/threonine-protein kinase (Fragment) OS	5	1	3.2430	0.0031
F8W7U0	ITSN1	129.9	Intersectin-1 OS	2	1	3.2300	0.0499
Q15717	ELAVL1	36.1	ELAV-like protein 1 OS	7	3	3.2110	0.0002
Q9NS86	LANCL2	50.8	LanC-like protein 2 OS	9	3	3.1970	0.0266
O14657	TOR1B	38	Torsin-1B OS	2	1	3.1930	0.1543
P11166	SLC2A1	54	Solute carrier family 2, facilitated glucose transporter member 1 OS	2	1	3.1740	0.1759
A0A2R8Y4C2	BCAR1	93.3	Breast cancer anti-estrogen resistance protein 1 OS	2	1	3.1670	0.0511
D6R9E3	MND1	19	Meiotic nuclear division protein 1 homolog OS	6	1	3.1350	0.1052
Q96AZ6	ISG20	20.4	Interferon-stimulated gene 20 kDa protein OS	4	1	3.1270	0.1070
B4E0K5	MAPK14	32.3	Mitogen-activated protein kinase 14 OS	21	4	3.1000	0.0629
Q9BYT8	NLN	80.6	Neurolysin, mitochondrial OS	7	4	3.0870	0.0066
C9JZY6	UBE2H	13.8	Ubiquitin-conjugating enzyme E2 H (Fragment) OS	12	1	3.0850	0.0045
P58546	MTPN	12.9	Myotrophin OS	87	7	3.0800	0.0000
K7ER83	PPP1R12C	26.8	Protein phosphatase 1 regulatory subunit 12C (Fragment) OS	7	1	3.0630	0.1133
A2A2V1	PRNP	27.3	Major prion protein (Fragment) OS	12	2	2.9990	0.0000
O43290	SART1	90.2	U4/U6.U5 tri-snRNP-associated protein 1 OS	5	2	2.9970	0.0511
P50995	ANXA11	54.4	Annexin A11 OS	4	1	2.9890	0.1235

Q5EBL4	RILPL1	47.1	RILP-like protein 1 OS	2	1	2.9820	0.1759
A0A0A0 MT32	LIPA	32.5	Lysosomal acid lipase/cholesteryl ester hydrolase OS	4	1	2.9660	0.0373
K7EKE6	LONP1	95.1	Lon protease homolog, mitochondrial OS	3	1	2.9420	0.0010
Q9NZL4	HSPBP1	39.3	Hsp70-binding protein 1 OS	11	3	2.9400	0.1408
Q5JTZ9	AARS2	107. 3	Alanine--tRNA ligase, mitochondrial OS	1	1	2.9280	0.0302
P11441	UBL4A	17.8	Ubiquitin-like protein 4A OS	18	3	2.9230	0.0841
O43719	HTATSF1	85.8	HIV Tat-specific factor 1 OS	3	1	2.9050	0.0009
P08590	MYL3	21.9	Myosin light chain 3 OS	8	1	2.8400	0.0008
Q9UEE9	CFDP1	33.6	Craniofacial development protein 1 OS	11	2	2.8370	0.0698
Q8TEQ6	GEMIN5	168. 5	Gem-associated protein 5 OS	3	3	2.8270	0.0394
Q86VM9	ZC3H18	106. 3	Zinc finger CCCH domain-containing protein 18 OS	2	1	2.8040	0.0892
Q9UI12	ATP6V1H	55.8	V-type proton ATPase subunit H OS	5	1	2.7990	0.1885
Q8NCC3	PLA2G15	46.6	Phospholipase A2 group XV OS	7	2	2.7950	0.0683
Q9NZ63	C9orf78	33.7	Telomere length and silencing protein 1 homolog OS	8	2	2.7300	0.2046
P35754	GLRX	11.8	Glutaredoxin-1 OS	27	2	2.7290	0.1821
Q8WVY7	UBLCP1	36.8	Ubiquitin-like domain-containing CTD phosphatase 1 OS	10	2	2.7220	0.2158
P26196	DDX6	54.4	Probable ATP-dependent RNA helicase DDX6 OS	15	4	2.7080	0.0226
Q9UBW8	COPS7A	30.3	COP9 signalosome complex subunit 7a OS	22	3	2.7030	0.1243
A0A087X 0M4	SLC4A1AP	82.8	Kanadaptin OS	1	1	2.6960	0.2742
P15529	CD46	43.7	Membrane cofactor protein OS	4	1	2.6810	0.0680
I3NI36	AKAP1	38.8	A-kinase anchor protein 1, mitochondrial (Fragment) OS	2	1	2.6660	0.0393
A0A0U1R RM6	ENAH	87.3	Protein enabled homolog OS	5	3	2.6450	0.1511
A0A2R8Y GH5	AP1S1	18.6	AP complex subunit sigma OS	4	1	2.6410	0.2677
Q6ZNA5	FRRS1	66.1	Ferric-chelate reductase 1 OS	1	1	2.6360	0.0970
P63151	PPP2R2A	51.7	Serine/threonine-protein phosphatase 2A 55 kDa regulatory subunit B alpha isoform OS	43	12	2.6170	0.0000
P00742	F10	54.7	Coagulation factor X OS	2	1	2.6100	0.0013
Q6P1J9	CDC73	60.5	Parafibromin OS	5	3	2.6040	0.1569
Q13158	FADD	23.3	FAS-associated death domain protein OS	13	1	2.5970	0.3658
Q15404	RSU1	31.5	Ras suppressor protein 1 OS	31	6	2.5710	0.0522
P08670	VIM	53.6	Vimentin OS	8	4	2.5350	0.3344
Q68DU8	KCTD16	49.1	BTB/POZ domain-containing protein KCTD16 OS	5	2	2.5220	0.2837
P02647	APOA1	30.8	Apolipoprotein A-I OS	6	1	2.5110	0.1885
Q9UBS4	DNAJB11	40.5	DnaJ homolog subfamily B member 11 OS	14	2	2.5010	0.2765
F5H365	SEC23A	82.9	Protein transport protein SEC23 OS	8	4	2.4690	0.3555
A0A087W YS3	ZMYND8	121. 2	Protein kinase C-binding protein 1 OS	1	1	2.4580	0.1759
Q9ULX6	AKAP8L	71.6	A-kinase anchor protein 8-like OS	1	1	2.4500	0.1021
Q9BS40	LXN	25.7	Latexin OS	4	1	2.4480	0.0086
Q15843	NEDD8	9.1	NEDD8 OS	74	6	2.4480	0.0000
P61088	UBE2N	17.1	Ubiquitin-conjugating enzyme E2 N OS	48	8	2.4370	0.0003
O75674	TOM1L1	53	TOM1-like protein 1 OS	5	1	2.4360	0.2218
A0A2R8Y 5Y7	RPL9	25	60S ribosomal protein L9 OS	26	3	2.3800	0.2528
P13284	IFI30	27.9	Gamma-interferon-inducible lysosomal thiol reductase OS	30	5	2.3500	0.0778
Q9UN81	L1RE1	40	LINE-1 retrotransposable element ORF1 protein OS	4	1	2.3230	0.1125
P15086	CPB1	47.3	Carboxypeptidase B OS	2	1	2.3180	0.3147
O75351	VPS4B	49.3	Vacuolar protein sorting-associated protein 4B OS	6	2	2.3110	0.1692

A0A087X0K1	CAB39	39.4	Calcium-binding protein 39 OS	10	3	2.3000	0.3048
P00750	PLAT	62.9	Tissue-type plasminogen activator OS	5	2	2.2870	0.0317
O95456	PSMG1	32.8	Proteasome assembly chaperone 1 OS	4	1	2.2860	0.4193
Q86X55	CARM1	65.8	Histone-arginine methyltransferase CARM1 OS	2	1	2.2820	0.4429
Q96ST3	SIN3A	145.1	Paired amphipathic helix protein Sin3a OS	2	1	2.2750	0.4920
P51580	TPMT	28.2	Thiopurine S-methyltransferase OS	3	1	2.2580	0.3570
Q15121	PEA15	15	Astrocytic phosphoprotein PEA-15 OS	28	2	2.2570	0.2335
Q9Y6U3	SCIN	80.4	Adseverin OS	3	1	2.2520	0.0058
O15498	YKT6	22.4	Synaptobrevin homolog YKT6 OS	11	2	2.2470	0.2015
A0A1W2PQS6	RPS10-NUDT3	33.1	RPS10-NUDT3 readthrough OS	19	5	2.2410	0.4022
Q9ULZ3	PYCARD	21.6	Apoptosis-associated speck-like protein containing a CARD OS	34	5	2.2400	0.2064
O15514	POLR2D	16.3	DNA-directed RNA polymerase II subunit RPB4 OS	32	2	2.2310	0.4997
P40818	USP8	127.4	Ubiquitin carboxyl-terminal hydrolase 8 OS	1	1	2.2260	0.3532
P46109	CRKL	33.8	Crk-like protein OS	32	8	2.2230	0.1257
O60884	DNAJA2	45.7	DnaJ homolog subfamily A member 2 OS	8	1	2.2180	0.3948
O75368	SH3BGRL	12.8	SH3 domain-binding glutamic acid-rich-like protein OS	54	6	2.2100	0.0036
P62312	LSM6	9.1	U6 snRNA-associated Sm-like protein LSm6 OS	26	2	2.2040	0.1292
P68032	ACTC1	42	Actin, alpha cardiac muscle 1 OS	68	56	2.1960	0.0001
O75506	HSBP1	8.5	Heat shock factor-binding protein 1 OS	49	1	2.1900	0.5205
Q9Y697	NFS1	50.2	Cysteine desulfurase, mitochondrial OS	3	1	2.1720	0.3673
P49959	MRE11	80.5	Double-strand break repair protein MRE11 OS	1	1	2.1630	0.4270
P80723	BASP1	22.7	Brain acid soluble protein 1 OS	23	2	2.1590	0.1625
P61960	UFM1	9.1	Ubiquitin-fold modifier 1 OS	73	4	2.1500	0.3948
B7ZAX5	GALK2	47.6	N-acetylgalactosamine kinase OS	2	1	2.1420	0.3439
P61006	RAB8A	23.7	Ras-related protein Rab-8A OS	17	3	2.1370	0.3090
P14735	IDE	117.9	Insulin-degrading enzyme OS	5	3	2.1320	0.4035
O75884	RBBP9	21	Serine hydrolase RBBP9 OS	33	3	2.1320	0.2687
Q5T0W9	FAM83B	114.7	Protein FAM83B OS	1	1	2.1260	0.3741
Q96T88	UHRF1	89.8	E3 ubiquitin-protein ligase UHRF1 OS	4	3	2.1150	0.1645
Q6P4F2	FDX2	19.9	Ferredoxin-2, mitochondrial OS	12	1	2.1110	0.4499
C9J4M6	POLR2B	121.3	DNA-directed RNA polymerase subunit beta OS	1	1	2.0920	0.0695
Q14CX7	NAA25	112.2	N-alpha-acetyltransferase 25, NatB auxiliary subunit OS	4	3	2.0850	0.1759
A5YKK6	CNOT1	266.8	CCR4-NOT transcription complex subunit 1 OS	0	1	2.0750	0.5307
Q9HAV4	XPO5	136.2	Exportin-5 OS	1	2	2.0750	0.4786
P54727	RAD23B	43.1	UV excision repair protein RAD23 homolog B OS	31	9	2.0640	0.0043
Q9BVM2	DPCD	23.2	Protein DPCD OS	5	1	2.0580	0.5458
Q96GG9	DCUN1D1	30.1	DCN1-like protein 1 OS	25	6	2.0560	0.1780
Q86Y82	STX12	31.6	Syntaxin-12 OS	22	4	2.0370	0.2218
P48556	PSMD8	39.6	26S proteasome non-ATPase regulatory subunit 8 OS	2	1	2.0320	0.3902
Q8TAQ2	SMARCC2	132.8	SWI/SNF complex subunit SMARCC2 OS	2	1	2.0220	0.4001
A6H8L4	ADAM17	29.9	ADAM17 protein OS	17	3	2.0180	0.2526
P51149	RAB7A	23.5	Ras-related protein Rab-7a OS	36	6	2.0020	0.0065
Q6UXN9	WDR82	35.1	WD repeat-containing protein 82 OS	19	4	1.9990	0.2164
F2Z2B1	PCYT1B	20.3	Choline-phosphate cytidylyltransferase B OS	9	1	1.9910	0.4920
Q96NH3	TBC1D32	144.7	Protein broad-minded OS	1	1	1.9910	0.2526

P63167	DYNLL1	10.4	Dynein light chain 1, cytoplasmic OS	61	5	1.9900	0.0249
Q92804	TAF15	61.8	TATA-binding protein-associated factor 2N OS	19	6	1.9890	0.0063
Q5VW32	BROX	46.4	BRO1 domain-containing protein BROX OS	16	4	1.9860	0.2013
Q9BSJ8	ESYT1	122.8	Extended synaptotagmin-1 OS	3	3	1.9780	0.1043
A0A1W2PQF8	ME2	53.6	Malic enzyme OS	3	1	1.9780	0.4033
H0YCN4	DCUN1D5	22.9	DCN1-like protein (Fragment) OS	4	1	1.9740	0.4837
Q96BP3	PPWD1	73.5	Peptidylprolyl isomerase domain and WD repeat-containing protein 1 OS	2	1	1.9740	0.4767
Q8WUI4	HDAC7	102.9	Histone deacetylase 7 OS	2	1	1.9640	0.2526
Q9BYN0	SRXN1	14.3	Sulfiredoxin-1 OS	29	2	1.9610	0.3204
Q96JB5	CDK5RAP3	56.9	CDK5 regulatory subunit-associated protein 3 OS	5	2	1.9600	0.5887
C9JNV3	LSM8	8.3	U6 snRNA-associated Sm-like protein LSm8 (Fragment) OS	48	3	1.9530	0.0058
A0A0B4J1R6	TKT	49.9	Transketolase OS	92	62	1.9520	0.5356
P13473	LAMP2	44.9	Lysosome-associated membrane glycoprotein 2 OS	4	2	1.9470	0.1282
P67870	CSNK2B	24.9	Casein kinase II subunit beta OS	30	3	1.9400	0.3757
Q6X4W1	NELF	60.1	NMDA receptor synaptonuclear signaling and neuronal migration factor OS	1	1	1.9370	0.4310
Q92542	NCSTN	78.4	Nicastrin OS	3	2	1.9330	0.4364
F5GYG5	REXO2	25.3	Oligoribonuclease, mitochondrial OS	30	8	1.9320	0.4920
A0A0A0MTR2	OGFOD1	58.3	Prolyl 3-hydroxylase OGFOD1 OS	7	3	1.9290	0.5137
Q5VV41	ARHGEF16	80.1	Rho guanine nucleotide exchange factor 16 OS	2	1	1.9260	0.4468
Q08752	PPID	40.7	Peptidyl-prolyl cis-trans isomerase D OS	12	4	1.9250	0.2927
Q9Y5Z4	HEBP2	22.9	Heme-binding protein 2 OS	41	4	1.9230	0.3786
A0A3B3ISV3	COL4A1	39.8	Collagen alpha-1(IV) chain (Fragment) OS	12	2	1.9210	0.4464
D6RAX7	COPS4	47.7	COP9 signalosome complex subunit 4 OS	30	10	1.9160	0.1510
O00203	AP3B1	121.2	AP-3 complex subunit beta-1 OS	4	3	1.9150	0.2742
P04217	A1BG	54.2	Alpha-1B-glycoprotein OS	1	1	1.9070	0.1110
O14936	CASK	105.1	Peripheral plasma membrane protein CASK OS	5	3	1.9070	0.2364
P55854	SUMO3	11.6	Small ubiquitin-related modifier 3 OS	75	4	1.9000	0.2014
Q9NWV4	C1orf123	18	CXXC motif containing zinc binding protein OS	34	4	1.8980	0.2086
P61626	LYZ	16.5	Lysozyme C OS	26	4	1.8980	0.1276
Q9BYE9	CDHR2	141.5	Cadherin-related family member 2 OS	1	1	1.8940	0.4819
P02778	CXCL10	10.9	C-X-C motif chemokine 10 OS	31	2	1.8940	0.1168
Q9H5V8	CDCP1	92.9	CUB domain-containing protein 1 OS	1	1	1.8830	0.5740
Q5T1J5	CHCHD2P9	15.5	Putative coiled-coil-helix-coiled-coil-helix domain-containing protein CHCHD2P9, mitochondrial OS	19	1	1.8760	0.4033
Q9H008	LHPP	29.1	Phospholysine phosphohistidine inorganic pyrophosphate phosphatase OS	11	2	1.8730	0.6555
P29317	EPHA2	108.2	Ephrin type-A receptor 2 OS	1	1	1.8660	0.5511
Q6P1N9	TATDN1	33.6	Putative deoxyribonuclease TATDN1 OS	9	2	1.8650	0.4945
Q9Y224	RTRAF	28.1	RNA transcription, translation and transport factor protein OS	44	8	1.8650	0.1793
A0A494BZV2	MPRIP	274.2	Myosin phosphatase Rho-interacting protein OS	1	1	1.8530	0.5881
Q5TDH0	DDI2	44.5	Protein DDI1 homolog 2 OS	4	1	1.8500	0.5108
A0A3B3IS71	RB1	105.4	Retinoblastoma-associated protein OS	1	2	1.8440	0.3781
Q63ZE4	SLC22A10	60.2	Solute carrier family 22 member 10 OS	3	2	1.8440	0.1558
A0A669KAX4	CUL4B	102.5	Cullin-4B OS	6	4	1.8370	0.0674
C9JKF1	SAMD9	148.7	Sterile alpha motif domain-containing protein 9 (Fragment) OS	5	4	1.8370	0.4493
H0Y8X6	NEDD4	104.7	HECT-type E3 ubiquitin transferase (Fragment) OS	7	3	1.8340	0.4501

P29622	SERPINA4	48.5	Kallistatin OS	2	1	1.8290	0.1680
P11279	LAMP1	44.9	Lysosome-associated membrane glycoprotein 1 OS	13	5	1.8240	0.0186
D6RBH1	LMAN2	23	Vesicular integral-membrane protein VIP36 (Fragment) OS	12	2	1.8210	0.4583
Q9UMX5	NENF	18.8	Neudesin OS	9	1	1.8200	0.4692
Q9P016	THYN1	25.7	Thymocyte nuclear protein 1 OS	4	1	1.8200	0.5345
Q9BZK7	TBL1XR1	55.6	F-box-like/WD repeat-containing protein TBL1XR1 OS	15	4	1.8130	0.0440
C9IZ80	BZW1	33.4	Basic leucine zipper and W2 domain-containing protein 1 (Fragment) OS	10	2	1.8110	0.4104
O76071	CIAO1	37.8	Probable cytosolic iron-sulfur protein assembly protein CIAO1 OS	6	2	1.8100	0.2227
P20742	PZP	163.8	Pregnancy zone protein OS	2	3	1.8080	0.6106
P10599	TXN	11.7	Thioredoxin OS	99	14	1.7990	0.0041
P29034	S100A2	11.1	Protein S100-A2 OS	43	5	1.7900	0.0167
P34896	SHMT1	53	Serine hydroxymethyltransferase, cytosolic OS	24	7	1.7880	0.0338
Q92783	STAM	59.1	Signal transducing adapter molecule 1 OS	3	2	1.7880	0.3977
E9PQU5	RBM25	26.7	RNA-binding protein 25 (Fragment) OS	10	2	1.7840	0.4836
P60002	ELOF1	9.5	Transcription elongation factor 1 homolog OS	22	1	1.7840	0.4901
P05161	ISG15	17.9	Ubiquitin-like protein ISG15 OS	18	3	1.7790	0.1133
A0A0A0MSB8	EXOC7	78.8	Exocyst complex component 7 OS	1	1	1.7770	0.0709
Q9P0L0	VAPA	27.9	Vesicle-associated membrane protein-associated protein A OS	38	7	1.7730	0.4528
P15291	B4GALT1	43.9	Beta-1,4-galactosyltransferase 1 OS	18	5	1.7690	0.1778
P60903	S100A10	11.2	Protein S100-A10 OS	49	3	1.7660	0.1030
P42224	STAT1	87.3	Signal transducer and activator of transcription 1-alpha/beta OS	31	17	1.7630	0.0162
P52565	ARHGDIA	23.2	Rho GDP-dissociation inhibitor 1 OS	75	15	1.7570	0.0070
P48745	NOV	39.1	CCN family member 3 OS	4	1	1.7560	0.5763
O95274	LYPD3	35.9	Ly6/PLAUR domain-containing protein 3 OS	18	4	1.7560	0.4930
F8WAN9	GMPR	38.2	GMP reductase OS	13	3	1.7510	0.3977
P68402	PAFAH1B2	25.6	Platelet-activating factor acetylhydrolase IB subunit beta OS	62	7	1.7510	0.0320
Q5T7F0	NRP1	79	Neuropilin OS	3	1	1.7480	0.6406
J3KQ18	DDT	14.2	D-dopachrome decarboxylase OS	33	5	1.7450	0.0083
Q53H82	LACTB2	32.8	Endoribonuclease LACTB2 OS	16	3	1.7430	0.4094
Q9ULC4	MCTS1	20.5	Malignant T-cell-amplified sequence 1 OS	54	7	1.7430	0.0447
F6SYF8	DKK3	39.9	Dickkopf-related protein 3 OS	30	7	1.7410	0.0414
P26572	MGAT1	50.8	Alpha-1,3-mannosyl-glycoprotein 2-beta-N-acetylglucosaminyltransferase OS	2	1	1.7400	0.7484
Q9GZN8	C20orf27	19.3	UPF0687 protein C20orf27 OS	25	4	1.7400	0.4800
Q13033	STRN3	87.2	Striatin-3 OS	2	1	1.7360	0.5192
P35637	FUS	53.4	RNA-binding protein FUS OS	25	8	1.7340	0.1670
Q9NQW6	ANLN	124.1	Anillin OS	1	1	1.7310	0.5915
Q9H7H0	METTL17	50.7	Methyltransferase-like protein 17, mitochondrial OS	2	1	1.7310	0.4198
Q13257	MAD2L1	23.5	Mitotic spindle assembly checkpoint protein MAD2A OS	5	1	1.7300	0.6277
A0A3B3ISS6	GPNMB	65.9	Transmembrane glycoprotein NMB OS	5	2	1.7290	0.5307
H3BT71	RBMX	32.2	RNA-binding motif protein, X chromosome OS	47	16	1.7260	0.0101
C9JLU1	POLR2H	16.9	DNA-directed RNA polymerases I, II, and III subunit RPABC3 (Fragment) OS	26	3	1.7250	0.1372
Q06323	PSME1	28.7	Proteasome activator complex subunit 1 OS	69	16	1.7210	0.0113
Q9H1B7	IRF2BPL	82.6	Probable E3 ubiquitin-protein ligase IRF2BPL OS	1	1	1.7200	0.5367
P61019	RAB2A	23.5	Ras-related protein Rab-2A OS	17	3	1.7180	0.2778
O15511	ARPC5	16.3	Actin-related protein 2/3 complex subunit 5 OS	60	5	1.7170	0.0567

P62316	SNRPD2	13.5	Small nuclear ribonucleoprotein Sm D2 OS	50	4	1.7160	0.2456
A0A2R8Y5S7	RDX	69.3	Radixin OS	54	41	1.7150	0.0446
G3V5X4	SYNE2	787.2	Nesprin-2 OS	1	3	1.7130	0.0462
E9PKG1	PRMT1	37.7	Protein arginine N-methyltransferase 1 OS	52	12	1.7120	0.0414
H7BZW7	CDC123	17.4	Cell division cycle protein 123 homolog (Fragment) OS	6	1	1.7100	0.6681
Q5QPM7	PSMF1	28.9	Proteasome inhibitor PI31 subunit OS	35	5	1.7050	0.3440
A0A140T8Y4	HLA-A	41.6	HLA class I histocompatibility antigen, A alpha chain OS	44	11	1.7030	0.1676
P05455	SSB	46.8	Lupus La protein OS	52	24	1.7030	0.0144
E9PS97	PARVA	23.6	Alpha-parvin (Fragment) OS	15	2	1.7020	0.7292
Q9P013	CWC15	26.6	Spliceosome-associated protein CWC15 homolog OS	16	2	1.7000	0.5465
O43823	AKAP8	76.1	A-kinase anchor protein 8 OS	3	1	1.6950	0.6567
E7EM64	COPS6	36	COP9 signalosome complex subunit 6 OS	17	3	1.6940	0.3031
Q9HCE9	ANO8	135.9	Anoctamin-8 OS	1	1	1.6930	0.1372
Q9UIA9	XPO7	123.8	Exportin-7 OS	3	2	1.6840	0.5567
Q9UNM6	PSMD13	42.9	26S proteasome non-ATPase regulatory subunit 13 OS	23	6	1.6830	0.3018
H0YH15	AQR	75.8	Aquarius homolog (Mouse), isoform CRA_a OS	1	1	1.6830	0.1214
P36871	PGM1	61.4	Phosphoglucomutase-1 OS	41	17	1.6820	0.0613
Q8NGI9	OR5A2	36	Olfactory receptor 5A2 OS	3	1	1.6780	0.0368
O43818	RRP9	51.8	U3 small nucleolar RNA-interacting protein 2 OS	6	3	1.6780	0.2364
A0A087WYW7	HIKESHI	14.4	Protein Hikeshi OS	12	1	1.6770	0.5740
Q9Y3X0	CCDC9	59.7	Coiled-coil domain-containing protein 9 OS	4	1	1.6720	0.0839
A0A087WT44	HMOX2	41.6	Heme oxygenase (biliverdin-producing) OS	27	6	1.6670	0.3470
P11940	PABPC1	70.6	Polyadenylate-binding protein 1 OS	50	26	1.6630	0.0236
E9PL69	RRM1	64.8	Ribonucleoside-diphosphate reductase large subunit OS	1	1	1.6610	0.7080
Q00577	PURA	34.9	Transcriptional activator protein Pur-alpha OS	20	7	1.6610	0.3232
Q15024	EXOSC7	31.8	Exosome complex component RRP42 OS	8	1	1.6580	0.7245
Q9H832	UBE2Z	38.2	Ubiquitin-conjugating enzyme E2 Z OS	17	4	1.6580	0.4851
Q9BQ67	GRWD1	49.4	Glutamate-rich WD repeat-containing protein 1 OS	7	2	1.6530	0.6406
P67809	YBX1	35.9	Y-box-binding protein 1 OS	73	14	1.6530	0.0268
Q13547	HDAC1	55.1	Histone deacetylase 1 OS	18	6	1.6490	0.6145
Q96I24	FUBP3	61.6	Far upstream element-binding protein 3 OS	11	4	1.6480	0.5681
Q92688	ANP32B	28.8	Acidic leucine-rich nuclear phosphoprotein 32 family member B OS	39	11	1.6470	0.0286
O15131	KPNA5	60.3	Importin subunit alpha-6 OS	2	1	1.6460	0.5980
Q6YHK3	CD109	161.6	CD109 antigen OS	28	30	1.6410	0.0304
P41208	CETN2	19.7	Centrin-2 OS	28	3	1.6400	0.6520
A6NGQ3	OBSCN	972.4	Obscurin OS	0	1	1.6400	0.7446
P31942	HNRNPH3	36.9	Heterogeneous nuclear ribonucleoprotein H3 OS	8	2	1.6380	0.7395
Q9H7Z7	PTGES2	41.9	Prostaglandin E synthase 2 OS	11	2	1.6350	0.7418
Q9H1D9	POLR3F	35.7	DNA-directed RNA polymerase III subunit RPC6 OS	3	1	1.6320	0.7494
A6NHR9	SMCHD1	226.2	Structural maintenance of chromosomes flexible hinge domain-containing protein 1 OS	1	1	1.6310	0.6213
H0Y2W2	ATAD3A	16.4	ATPase family AAA domain-containing protein 3A (Fragment) OS	12	1	1.6230	0.7481
Q8IWX8	CHERP	103.6	Calcium homeostasis endoplasmic reticulum protein OS	3	2	1.6230	0.5734
C9J0H3	PLSCR1	19.4	Phospholipid scramblase (Fragment) OS	8	1	1.6220	0.6774
P05387	RPLP2	11.7	60S acidic ribosomal protein P2 OS	100	16	1.6210	0.0385
P05026	ATP1B1	35	Sodium/potassium-transporting ATPase subunit beta-1 OS	10	1	1.6210	0.7605

P36639	NUDT1	22.5	7,8-dihydro-8-oxoguanine triphosphatase OS	9	1	1.6200	0.7446
Q15746	MYLK	210.6	Myosin light chain kinase, smooth muscle OS	0	1	1.6200	0.6267
Q5TCU3	TPM2	32.8	Tropomyosin beta chain OS	32	12	1.6190	0.0871
Q5VU77	UBAP2L	37.9	Ubiquitin-associated protein 2-like (Fragment) OS	16	5	1.6190	0.3682
Q9Y6A5	TACC3	90.3	Transforming acidic coiled-coil-containing protein 3 OS	3	2	1.6090	0.6721
Q92882	OSTF1	23.8	Osteoclast-stimulating factor 1 OS	40	9	1.6070	0.1244
P21589	NT5E	63.3	5'-nucleotidase OS	7	2	1.6060	0.7431
P21741	MDK	15.6	Midkine OS	58	13	1.6040	0.0462
P38435	GGCX	87.5	Vitamin K-dependent gamma-carboxylase OS	1	1	1.6030	0.7437
Q96EK6	GNPNAT1	20.7	Glucosamine 6-phosphate N-acetyltransferase OS	51	6	1.6000	0.3941
O75937	DNAJC8	29.8	DnaJ homolog subfamily C member 8 OS	58	9	1.5940	0.1595
Q9Y5L4	TIMM13	10.5	Mitochondrial import inner membrane translocase subunit Tim13 OS	41	3	1.5940	0.4836
G3V3E8	NPC2	19.2	Epididymal secretory protein E1 OS	48	14	1.5930	0.0516
O60502	MGEA5	102.8	Protein O-GlcNAcase OS	6	3	1.5920	0.6817
O00170	AIP	37.6	AH receptor-interacting protein OS	21	5	1.5890	0.5374
O43747	AP1G1	91.3	AP-1 complex subunit gamma-1 OS	6	4	1.5890	0.4327
Q9Y6E2	BZW2	48.1	Basic leucine zipper and W2 domain-containing protein 2 OS	20	6	1.5880	0.2019
Q9H6T3	RPAP3	75.7	RNA polymerase II-associated protein 3 OS	3	2	1.5860	0.7437
K7ERQ2	FAM210A	20	Protein FAM210A (Fragment) OS	4	1	1.5810	0.6384
P41223	BUD31	17	Protein BUD31 homolog OS	13	3	1.5790	0.6023
P68036	UBE2L3	17.9	Ubiquitin-conjugating enzyme E2 L3 OS	77	9	1.5780	0.3768
A0A669KBI5	NIN	68.8	Ninein OS	2	1	1.5670	0.5884
P46976	GYG1	39.4	Glycogenin-1 OS	15	4	1.5640	0.4627
O15230	LAMA5	399.5	Laminin subunit alpha-5 OS	11	28	1.5620	0.0873
H0YN07	IPO4	33.2	Importin-4 (Fragment) OS	4	1	1.5610	0.8154
O95816	BAG2	23.8	BAG family molecular chaperone regulator 2 OS	33	4	1.5590	0.6670
B5MCT8	RPS9	16.6	40S ribosomal protein S9 OS	12	2	1.5570	0.5677
Q96Q89	KIF20B	210.5	Kinesin-like protein KIF20B OS	0	1	1.5570	0.0756
Q9H788	SH2D4A	52.7	SH2 domain-containing protein 4A OS	3	1	1.5570	0.6406
Q14141	SEPT6	49.7	Septin-6 OS	13	5	1.5530	0.6796
O95817	BAG3	61.6	BAG family molecular chaperone regulator 3 OS	35	13	1.5520	0.1712
P22392	NME2	17.3	Nucleoside diphosphate kinase B OS	93	22	1.5500	0.0806
Q9UBQ0	VPS29	20.5	Vacuolar protein sorting-associated protein 29 OS	43	7	1.5480	0.4454
P61024	CKS1B	9.7	Cyclin-dependent kinases regulatory subunit 1 OS	33	2	1.5470	0.4122
P61978	HNRNPK	50.9	Heterogeneous nuclear ribonucleoprotein K OS	76	35	1.5440	0.6188
O75608	LYPLA1	24.7	Acyl-protein thioesterase 1 OS	31	5	1.5430	0.3446
P80188	LCN2	22.6	Neutrophil gelatinase-associated lipocalin OS	84	25	1.5430	0.0871
Q14512	FGFBP1	26.2	Fibroblast growth factor-binding protein 1 OS	26	5	1.5410	0.3034
Q9BQL6	FERMT1	77.4	Fermitin family homolog 1 OS	9	4	1.5400	0.5803
O14617	AP3D1	130.1	AP-3 complex subunit delta-1 OS	1	2	1.5350	0.7692
P01116	KRAS	21.6	GTPase KRas OS	6	1	1.5340	0.7446
E9PHS0	FKBP3	22	Glutathione S-transferase LANCL1 (Fragment) OS	27	4	1.5320	0.6507
Q00688	FKBP3	25.2	Peptidyl-prolyl cis-trans isomerase FKBP3 OS	54	14	1.5320	0.0995
Q99757	TXN2	18.4	Thioredoxin, mitochondrial OS	8	1	1.5320	0.7437
Q6IBS0	TWF2	39.5	Twinfilin-2 OS	33	6	1.5310	0.3984

E5RI99	RPA2	12.6	60S ribosomal protein L30 (Fragment) OS	44	4	1.5280	0.2953
P15927	RPA2	29.2	Replication protein A 32 kDa subunit OS	41	8	1.5280	0.5884
Q9Y547	HSPB11	16.3	Intraflagellar transport protein 25 homolog OS	18	2	1.5230	0.7582
Q9Y333	LSM2	10.8	U6 snRNA-associated Sm-like protein LSm2 OS	49	4	1.5230	0.3900
A0A5F9ZI63	EED	56.2	Polycomb protein EED OS	2	1	1.5220	0.8107
Q9NX08	COMMD8	21.1	COMM domain-containing protein 8 OS	7	1	1.5210	0.7686
J3QQQ9	N/A	12.7	KOW domain-containing protein OS	31	3	1.5210	0.2464
O60664	PLIN3	47	Perilipin-3 OS	62	16	1.5190	0.2116
P54296	MYOM2	164.8	Myomesin-2 OS	1	1	1.5170	0.6428
P21399	ACO1	98.3	Cytoplasmic aconitate hydratase OS	1	1	1.5160	0.7735
P17050	NAGA	46.5	Alpha-N-acetylgalactosaminidase OS	2	1	1.5150	0.7494
Q9NRN7	AASDHPPT	35.8	L-aminoadipate-semialdehyde dehydrogenase-phosphopantetheinyl transferase OS	21	6	1.5150	0.4901
O00468-6	AGRN	214.7	Isoform 6 of Agrin OS	40	53	1.5130	0.1190
P49720	PSMB3	22.9	Proteasome subunit beta type-3 OS	55	15	1.5120	0.1197
P49902	NT5C2	64.9	Cytosolic purine 5'-nucleotidase OS	14	4	1.5110	0.6500
A0A1W2PRU0	ENSA	13.1	Alpha-endosulfine OS	32	2	1.5100	0.7494
Q01581	HMGCS1	57.3	Hydroxymethylglutaryl-CoA synthase, cytoplasmic OS	3	1	1.5100	0.7196
O94973	AP2A2	103.9	AP-2 complex subunit alpha-2 OS	4	4	1.5080	0.6480
Q16401	PSMD5	56.2	26S proteasome non-ATPase regulatory subunit 5 OS	40	16	1.5010	0.2116
Q96PD2	DCBLD2	85	Discoidin, CUB and LCCL domain-containing protein 2 OS	6	4	1.5000	0.5106
E9PN81	RNASEH2C	26.3	Ribonuclease H2 subunit C OS	6	1	1.5000	0.7811
A0A140T9U0	HLA-C	41.4	HLA class I histocompatibility antigen, C alpha chain OS	46	16	1.4990	0.1362
Q14C86	GAPVD1	164.9	GTPase-activating protein and VPS9 domain-containing protein 1 OS	2	2	1.4980	0.7240
Q12888	TP53BP1	213.4	TP53-binding protein 1 OS	9	10	1.4960	0.3806
Q96GX9	APIP	27.1	Methylthioribulose-1-phosphate dehydratase OS	29	5	1.4940	0.4920
P36955	SERPINF1	46.3	Pigment epithelium-derived factor OS	14	5	1.4940	0.5265
H0YLR3	SNRPA1	9.5	U2 small nuclear ribonucleoprotein A' (Fragment) OS	15	2	1.4930	0.7100
O75436	VPS26A	38.1	Vacuolar protein sorting-associated protein 26A OS	32	8	1.4930	0.1430
H0YEH1	PICALM	32.2	Phosphatidylinositol-binding clathrin assembly protein (Fragment) OS	6	1	1.4900	0.7874
Q12996	CSTF3	82.9	Cleavage stimulation factor subunit 3 OS	16	7	1.4870	0.6639
P42677	RPS27	9.5	40S ribosomal protein S27 OS	40	3	1.4860	0.5512
O14907	TAX1BP3	13.7	Tax1-binding protein 3 OS	31	4	1.4840	0.3857
O95292	VAPB	27.2	Vesicle-associated membrane protein-associated protein B/C OS	45	8	1.4820	0.2742
P11047	LAMC1	177.5	Laminin subunit gamma-1 OS	30	35	1.4810	0.1590
Q03519	TAP2	75.6	Antigen peptide transporter 2 OS	6	1	1.4800	0.8398
P08047	SP1	80.6	Transcription factor Sp1 OS	4	1	1.4800	0.7484
O43707	ACTN4	104.8	Alpha-actinin-4 OS	84	85	1.4790	0.1625
F8VUA2	CHMP1A	19.5	Charged multivesicular body protein 1a OS	10	2	1.4790	0.7709
P02786	TFRC	84.8	Transferrin receptor protein 1 OS	40	30	1.4760	0.1658
P00492	HPRT1	24.6	Hypoxanthine-guanine phosphoribosyltransferase OS	71	17	1.4740	0.1692
O00193	C11orf58	20.3	Small acidic protein OS	27	5	1.4730	0.1713
P60604	UBE2G2	18.6	Ubiquitin-conjugating enzyme E2 G2 OS	9	1	1.4730	0.7735
P23284	PPIB	23.7	Peptidyl-prolyl cis-trans isomerase B OS	72	22	1.4720	0.1725
P62861	FAU	6.6	40S ribosomal protein S30 OS	17	1	1.4710	0.5848
J3KN16	KIAA0368	223.6	Proteasome adapter and scaffold protein ECM29 OS	10	15	1.4710	0.2852

O95486	SEC24A	119.7	Protein transport protein Sec24A OS	2	1	1.4710	0.8051
C9JKY3	EPCAM	20.9	Epithelial cell adhesion molecule (Fragment) OS	28	4	1.4700	0.4721
P18887	XRCC1	69.4	DNA repair protein XRCC1 OS	2	1	1.4670	0.5677
Q9BUR4	WRAP53	59.3	Telomerase Cajal body protein 1 OS	3	1	1.4670	0.8313
P31689	DNAJA1	44.8	DnaJ homolog subfamily A member 1 OS	5	2	1.4660	0.7494
P25789	PSMA4	29.5	Proteasome subunit alpha type-4 OS	75	22	1.4630	0.1849
A0A087WU76	HSD11B1	19.9	Corticosteroid 11-beta-dehydrogenase isozyme 1 (Fragment) OS	7	1	1.4600	0.4493
P62258	YWHAE	29.2	14-3-3 protein epsilon OS	81	32	1.4580	0.1929
P67936-2	TPM4	32.7	Isoform 2 of Tropomyosin alpha-4 chain OS	53	21	1.4580	0.2709
Q16222	UAP1	58.7	UDP-N-acetylhexosamine pyrophosphorylase OS	9	4	1.4570	0.8080
P42566	EPS15	98.6	Epidermal growth factor receptor substrate 15 OS	4	2	1.4550	0.7494
P42766	RPL35	14.5	60S ribosomal protein L35 OS	22	2	1.4540	0.7446
O00339	MATN2	106.8	Matrilin-2 OS	5	4	1.4510	0.6277
O15116	LSM1	15.2	U6 snRNA-associated Sm-like protein LSm1 OS	17	2	1.4510	0.7679
A0A1B0GW68	ASAH1	42.3	Acid ceramidase OS	23	6	1.4490	0.6106
P48163	ME1	64.1	NADP-dependent malic enzyme OS	47	14	1.4490	0.3900
P20618	PSMB1	26.5	Proteasome subunit beta type-1 OS	59	16	1.4490	0.2091
P25685	DNAJB1	38	DnaJ homolog subfamily B member 1 OS	13	3	1.4480	0.7670
O75874	IDH1	46.6	Isocitrate dehydrogenase [NADP] cytoplasmic OS	69	29	1.4480	0.2106
Q14677	CLINT1	68.2	Clathrin interactor 1 OS	3	1	1.4470	0.7838
A0A3B3ISQ4	EPS8L2	88	Epidermal growth factor receptor kinase substrate 8-like protein 2 OS	12	5	1.4470	0.6208
O95793	STAU1	63.1	Double-stranded RNA-binding protein Staufen homolog 1 OS	2	1	1.4450	0.7851
P55060	CSE1L	110.3	Exportin-2 OS	37	29	1.4440	0.2175
Q92878	RAD50	153.8	DNA repair protein RAD50 OS	1	1	1.4420	0.8344
P51991	HNRNPA3	39.6	Heterogeneous nuclear ribonucleoprotein A3 OS	37	9	1.4420	0.2218
P52735	VAV2	101.2	Guanine nucleotide exchange factor VAV2 OS	2	1	1.4400	0.8610
J3KMZ9	LDLR	104.6	Low-density lipoprotein receptor (Fragment) OS	12	9	1.4400	0.3055
G3V1R5	NRD1	125	Nardilysin OS	5	5	1.4400	0.6305
P62306	SNRPF	9.7	Small nuclear ribonucleoprotein F OS	24	3	1.4400	0.5897
Q9HB71	CACYBP	26.2	Calcyclin-binding protein OS	82	20	1.4390	0.2256
Q02809	PLOD1	83.5	Procollagen-lysine,2-oxoglutarate 5-dioxygenase 1 OS	22	13	1.4390	0.3566
Q96TA1	FAM129B	84.1	Protein Niban 2 OS	43	20	1.4380	0.2270
A0A0C4DG51	PNPLA8	71.8	Calcium-independent phospholipase A2-gamma (Fragment) OS	1	1	1.4370	0.8165
B0S8I6	FAM50A	30.7	Protein FAM50A (Fragment) OS	11	3	1.4360	0.7437
Q09028	RBBP4	47.6	Histone-binding protein RBBP4 OS	35	11	1.4350	0.2625
P15289	ARSA	53.6	Arylsulfatase A OS	9	3	1.4340	0.7725
P07942	LAMB1	197.9	Laminin subunit beta-1 OS	16	16	1.4340	0.3774
Q01085	TIAL1	41.6	Nucleolysin TIAR OS	3	1	1.4330	0.7811
Q96NY8	NECTIN4	55.4	Nectin-4 OS	5	2	1.4320	0.8211
P50479	PDLIM4	35.4	PDZ and LIM domain protein 4 OS	7	2	1.4320	0.7316
P07919	UQCRH	10.7	Cytochrome b-c1 complex subunit 6, mitochondrial OS	20	1	1.4310	0.8265
P02765	AHSG	39.3	Alpha-2-HS-glycoprotein OS	9	6	1.4290	0.2464
P01033	TIMP1	23.2	Metalloproteinase inhibitor 1 OS	57	13	1.4290	0.2464
P54709	ATP1B3	31.5	Sodium/potassium-transporting ATPase subunit beta-3 OS	18	3	1.4290	0.6490
Q14008	CKAP5	225.4	Cytoskeleton-associated protein 5 OS	3	5	1.4270	0.6414

Q92805	GOLGA1	88.1	Golgin subfamily A member 1 OS	2	1	1.4270	0.7735
O14745	SLC9A3R1	38.8	Na(+)/H(+) exchange regulatory cofactor NHE-RF1 OS	44	9	1.4270	0.3795
A0A024R 6I7	SERPINA1	46.7	Alpha-1-antitrypsin OS	2	1	1.4250	0.6335
Q9NX55	HYPK	14.7	Huntingtin-interacting protein K OS	49	4	1.4250	0.5849
P23229	ITGA6	126. 5	Integrin alpha-6 OS	11	9	1.4250	0.3944
Q15631	TSN	26.2	Translin OS	57	10	1.4250	0.3424
O00244	ATOX1	7.4	Copper transport protein ATOX1 OS	90	6	1.4240	0.3682
Q9H993	ARMT1	51.1	Damage-control phosphatase ARMT1 OS	10	4	1.4240	0.7494
Q9BS19	HPX	28.6	Epididymis secretory sperm binding protein OS	9	2	1.4240	0.6848
Q9P1F3	ABRACL	9.1	Costars family protein ABRACL OS	40	2	1.4230	0.7992
H0Y8C4	PPP2R5D	59.4	Serine/threonine-protein phosphatase 2A 56 kDa regulatory subunit delta isoform (Fragment) OS	4	2	1.4230	0.3883
E7EV45	SLC39A10	15.3	Zinc transporter ZIP10 (Fragment) OS	6	1	1.4230	0.8260
P15374	UCHL3	26.2	Ubiquitin carboxyl-terminal hydrolase isozyme L3 OS	46	9	1.4220	0.2584
O75717	WDHD1	125. 9	WD repeat and HMG-box DNA-binding protein 1 OS	5	4	1.4220	0.6392
Q8NBJ4	GOLM1	45.3	Golgi membrane protein 1 OS	16	6	1.4200	0.5307
Q12905	ILF2	43	Interleukin enhancer-binding factor 2 OS	67	13	1.4200	0.2622
A0A140T 902	TNXB	455. 9	Tenascin-X OS	0	1	1.4200	0.5414
Q96C23	GALM	37.7	Galactose mutarotase OS	23	4	1.4190	0.7133
A0A0J9Y YL3	PUF60	54.6	60 kDa poly(U)-binding-splicing factor (Fragment) OS	46	13	1.4170	0.4073
E7ETU9	PLOD2	81.1	Procollagen-lysine 5-dioxygenase OS	9	4	1.4170	0.7527
B4DF77	PACS1	54.4	Phosphofurin acidic cluster sorting protein 1 OS	2	1	1.4150	0.8441
P08253	MMP2	73.8	72 kDa type IV collagenase OS	9	5	1.4140	0.7290
Q13325	IFIT5	55.8	Interferon-induced protein with tetratricopeptide repeats 5 OS	4	1	1.4140	0.8313
A0A087X 1Z3	PSME2	29.1	Proteasome activator complex subunit 2 OS	77	18	1.4130	0.2762
A2A2V4	VEGFA	19	Vascular endothelial growth factor A OS	55	4	1.4130	0.6555
P53004	BLVRA	33.4	Biliverdin reductase A OS	40	9	1.4120	0.4327
O75663	TIPRL	31.4	TIP41-like protein OS	46	9	1.4120	0.5677
J3KRC4	NT5C	20.4	5'(3')-deoxyribonucleotidase, cytosolic type OS	10	1	1.4110	0.8779
Q86TI2	DPP9	98.2	Dipeptidyl peptidase 9 OS	5	3	1.4110	0.8111
P17931	LGALS3	26.1	Galectin-3 OS	40	10	1.4110	0.3785
P41250	GARS	83.1	Glycine--tRNA ligase OS	59	37	1.4100	0.2811
M0QYT0	N/A	36	RRM domain-containing protein (Fragment) OS	31	8	1.4100	0.8344
O14896	IRF6	53.1	Interferon regulatory factor 6 OS	32	12	1.4080	0.3900
P26885	FKBP2	15.6	Peptidyl-prolyl cis-trans isomerase FKBP2 OS	19	3	1.4080	0.6539
O60597	IDS	19.4	Iduronate 2-sulfatase OS	5	1	1.4070	0.8659
Q96B36	AKT1S1	27.4	Proline-rich AKT1 substrate 1 OS	5	1	1.4070	0.8398
P55010	EIF5	49.2	Eukaryotic translation initiation factor 5 OS	33	14	1.4060	0.2892
P50579	METAP2	52.9	Methionine aminopeptidase 2 OS	30	8	1.4060	0.4035
P62851	RPS25	13.7	40S ribosomal protein S25 OS	18	3	1.4050	0.3790
P20591	MX1	75.5	Interferon-induced GTP-binding protein Mx1 OS	14	6	1.4050	0.7359
F5GZ78	PXN	64.2	Paxillin OS	11	4	1.4050	0.7798
Q9NR50	EIF2B3	50.2	Translation initiation factor eIF-2B subunit gamma OS	2	1	1.4050	0.8373
P54652	HSPA2	70	Heat shock-related 70 kDa protein 2 OS	23	22	1.4020	0.7527
A0A0A0 MQU1	INF2	78.8	Inverted formin-2 (Fragment) OS	3	2	1.4010	0.7700
P46063	RECQL	73.4	ATP-dependent DNA helicase Q1 OS	6	3	1.3990	0.6784

O95433	AHSA1	38.3	Activator of 90 kDa heat shock protein ATPase homolog 1 OS	52	14	1.3980	0.3055
P07355	ANXA2	38.6	Annexin A2 OS	91	52	1.3980	0.3058
Q13951-2	CBFB	22	Isoform 2 of Core-binding factor subunit beta OS	39	5	1.3980	0.4310
Q12874	SF3A3	58.8	Splicing factor 3A subunit 3 OS	32	13	1.3970	0.4468
Q9Y3C4	TPRKB	19.6	EKC/KEOPS complex subunit TPRKB OS	14	2	1.3960	0.8610
P30040	ERP29	29	Endoplasmic reticulum resident protein 29 OS	41	10	1.3950	0.3980
Q9HCY8	S100A14	11.7	Protein S100-A14 OS	46	4	1.3930	0.4139
Q92820	GGH	35.9	Gamma-glutamyl hydrolase OS	30	7	1.3920	0.4212
P28062	PSMB8	30.3	Proteasome subunit beta type-8 OS	51	9	1.3920	0.3427
O43583	DENR	22.1	Density-regulated protein OS	44	8	1.3890	0.4661
Q04760	GLO1	20.8	Lactoylglutathione lyase OS	83	15	1.3890	0.3305
P41567	EIF1	12.7	Eukaryotic translation initiation factor 1 OS	86	6	1.3880	0.4097
P15531	NME1	17.1	Nucleoside diphosphate kinase A OS	95	18	1.3870	0.3330
P46783	RPS10	18.9	40S ribosomal protein S10 OS	32	6	1.3860	0.3471
P04179-3	SOD2	18.3	Isoform 3 of Superoxide dismutase [Mn], mitochondrial OS	70	7	1.3860	0.8536
B7ZC39	SH3GLB2	41.6	Endophilin-B2 OS	9	3	1.3850	0.7987
Q15056	EIF4H	27.4	Eukaryotic translation initiation factor 4H OS	23	4	1.3850	0.3405
Q5T5C7	SARS	61.3	Seryl-tRNA synthetase OS	24	11	1.3850	0.4834
V9GZ56	LSM4	25.7	U6 snRNA-associated Sm-like protein LSm4 (Fragment) OS	13	4	1.3850	0.7110
Q9NZP5	OR5AC2	35.3	Olfactory receptor 5AC2 OS	2	1	1.3840	0.7245
Q14192	FHL2	32.2	Four and a half LIM domains protein 2 OS	39	7	1.3830	0.6406
Q9UBQ7	GRHPR	35.6	Glyoxylate reductase/hydroxypyruvate reductase OS	14	2	1.3830	0.8058
Q9BQA1	WDR77	36.7	Methylosome protein 50 OS	10	3	1.3830	0.8051
P10145	IL8; CXCL8	11.1	Interleukin-8 OS	34	3	1.3820	0.4781
P28070	PSMB4	29.2	Proteasome subunit beta type-4 OS	52	12	1.3820	0.3470
A0A087W V26	SSNA1	9.2	Sjoegren syndrome nuclear autoantigen 1 OS	38	2	1.3820	0.7798
F5GYK0	TRIM46	69.5	Tripartite motif-containing protein 46 OS	2	2	1.3820	0.3560
E9PKN4	CSDE1	5.1	Cold shock domain-containing protein E1 (Fragment) OS	28	1	1.3810	0.8265
P02545-2	LMNA	65.1	Isoform C of Prelamin-A/C OS	85	81	1.3800	0.4703
P05388	RPLP0	34.3	60S acidic ribosomal protein P0 OS	70	21	1.3790	0.3555
Q5BKZ1	ZNF326	65.6	DBIRD complex subunit ZNF326 OS	3	1	1.3790	0.8265
O14672	ADAM10	84.1	Disintegrin and metalloproteinase domain-containing protein 10 OS	9	7	1.3790	0.4961
J3KN66	TOR1AIP1	67.8	Torsin-1A-interacting protein 1 OS	8	3	1.3790	0.6750
Q86SQ4	ADGRG6	136.6	Adhesion G-protein coupled receptor G6 OS	2	2	1.3750	0.7245
Q14103	HNRNPD	38.4	Heterogeneous nuclear ribonucleoprotein D0 OS	45	25	1.3750	0.3658
Q96IZ0	PAWR	36.5	PRKC apoptosis WT1 regulator protein OS	14	3	1.3750	0.7224
O60248	SOX15	25.2	Protein SOX-15 OS	14	2	1.3750	0.7494
Q6GMV3	PTRHD1	15.8	Putative peptidyl-tRNA hydrolase PTRHD1 OS	15	1	1.3750	0.8188
P42166	TMPO	75.4	Lamina-associated polypeptide 2, isoform alpha OS	27	14	1.3740	0.3681
Q14914	PTGR1	35.8	Prostaglandin reductase 1 OS	42	10	1.3740	0.3977
O00754	MAN2B1	113.7	Lysosomal alpha-mannosidase OS	7	5	1.3730	0.7872
G3V2U7	ACYP1	14.1	Acylphosphatase OS	5	1	1.3710	0.8332
A0A140T 9R1	FLOT1	39.8	Flotillin (Fragment) OS	6	1	1.3710	0.8344
O75312	ZPR1	50.9	Zinc finger protein ZPR1 OS	24	7	1.3700	0.5508
P08581	MET	155.4	Hepatocyte growth factor receptor OS	15	15	1.3690	0.4591

Q5SS57	HLA-B	40.3	HLA class I antigen OS	37	11	1.3690	0.3801
O00267	SUPT5H	120.9	Transcription elongation factor SPT5 OS	2	1	1.3690	0.8128
B4DR80	STK24	45.8	Serine/threonine-protein kinase 24 OS	33	10	1.3660	0.5021
P08243	ASNS	64.3	Asparagine synthetase [glutamine-hydrolyzing] OS	6	4	1.3640	0.8163
Q13287	NMI	35	N-myc-interactor OS	16	3	1.3630	0.8153
Q13617	CUL2	86.9	Cullin-2 OS	4	2	1.3620	0.8363
B7Z2U2	TOM1L2	42.9	TOM1-like protein 2 OS	6	1	1.3610	0.8593
P47813	EIF1AX	16.5	Eukaryotic translation initiation factor 1A, X-chromosomal OS	40	4	1.3600	0.6332
P61204	ARF3	20.6	ADP-ribosylation factor 3 OS	60	7	1.3590	0.7843
O43237	DYNC1LI2	54.1	Cytoplasmic dynein 1 light intermediate chain 2 OS	1	1	1.3580	0.8398
B7ZKJ8	ITIH4	103.8	ITIH4 protein OS	3	3	1.3580	0.7384
P27816	MAP4	120.9	Microtubule-associated protein 4 OS	21	16	1.3560	0.4781
Q9Y4L1	HYOU1	111.3	Hypoxia up-regulated protein 1 OS	16	9	1.3550	0.7446
C9J2X2	MAGEA11	32.8	Melanoma-associated antigen 11 (Fragment) OS	2	1	1.3540	0.8588
Q13098	GPS1	55.5	COP9 signalosome complex subunit 1 OS	9	3	1.3530	0.7811
P00387	CYB5R3	34.2	NADH-cytochrome b5 reductase 3 OS	11	2	1.3530	0.7679
I3L4C2	BAIAP2	61.3	Brain-specific angiogenesis inhibitor 1-associated protein 2 OS	8	4	1.3510	0.7798
P28838	LAP3	56.1	Cytosol aminopeptidase OS	60	28	1.3510	0.4207
K7ESQ2	SPC24	23.5	Kinetochore protein Spc24 (Fragment) OS	4	1	1.3510	0.8581
P06730	EIF4E	25.1	Eukaryotic translation initiation factor 4E OS	35	6	1.3500	0.5870
Q16543	CDC37	44.4	Hsp90 co-chaperone Cdc37 OS	40	12	1.3500	0.4267
Q9Y6W5	WASF2	54.3	Wiskott-Aldrich syndrome protein family member 2 OS	7	4	1.3500	0.7446
Q96C90	PPP1R14B	15.9	Protein phosphatase 1 regulatory subunit 14B OS	64	8	1.3490	0.4271
O60506	SYNCRIP	69.6	Heterogeneous nuclear ribonucleoprotein Q OS	51	29	1.3480	0.4295
P08758	ANXA5	35.9	Annexin A5 OS	56	16	1.3470	0.5077
Q9H0D6	XRN2	108.5	5'-3' exoribonuclease 2 OS	14	8	1.3460	0.5503
H0YHX9	NACA	25.3	Nascent polypeptide-associated complex subunit alpha OS	31	7	1.3460	0.4271
G3V220	TCERG1	116.1	Transcription elongation regulator 1 OS	4	3	1.3460	0.8313
O43395	PRPF3	77.5	U4/U6 small nuclear ribonucleoprotein Prp3 OS	5	3	1.3460	0.7874
F5GZS6	SLC3A2	64.8	4F2 cell-surface antigen heavy chain OS	42	28	1.3440	0.4416
O00410	IPO5	123.6	Importin-5 OS	46	35	1.3440	0.4416
Q9NZM1	MYOF	234.6	Myoferlin OS	5	7	1.3420	0.7725
Q58FF6	HSP90AB4P	58.2	Putative heat shock protein HSP 90-beta 4 OS	17	8	1.3420	0.8158
P35268	RPL22	14.8	60S ribosomal protein L22 OS	51	5	1.3410	0.5614
H0YB99	PTK2	35.9	Non-specific protein-tyrosine kinase (Fragment) OS	9	1	1.3410	0.9228
O14737	PDCD5	14.3	Programmed cell death protein 5 OS	52	8	1.3410	0.4908
P62857	RPS28	7.8	40S ribosomal protein S28 OS	80	9	1.3400	0.4537
Q8NCW5	NAXE	31.7	NAD(P)H-hydrate epimerase OS	41	10	1.3400	0.5393
P52298	NCBP2	18	Nuclear cap-binding protein subunit 2 OS	16	2	1.3400	0.7975
Q9Y383	LUC7L2	46.5	Putative RNA-binding protein Luc7-like 2 OS	15	4	1.3400	0.6979
O96019	ACTL6A	47.4	Actin-like protein 6A OS	40	10	1.3390	0.5677
Q12805	EFEMP1	54.6	EGF-containing fibulin-like extracellular matrix protein 1 OS	47	18	1.3380	0.4591
P12814	ACTN1	103	Alpha-actinin-1 OS	83	87	1.3370	0.4594
P26358	DNMT1	183.1	DNA (cytosine-5)-methyltransferase 1 OS	1	1	1.3370	0.8742
Q02818	NUCB1	53.8	Nucleobindin-1 OS	65	27	1.3370	0.4594

E9PQY2	PFDN4	15.6	Prefoldin subunit 4 OS	46	7	1.3370	0.6670
P31948	STIP1	62.6	Stress-induced-phosphoprotein 1 OS	65	46	1.3370	0.4591
Q08380	LGALS3BP	65.3	Galectin-3-binding protein OS	49	34	1.3360	0.4622
P06737	PYGL	97.1	Glycogen phosphorylase, liver form OS	74	72	1.3360	0.4615
Q9BXS6	NUSAP1	49.4	Nucleolar and spindle-associated protein 1 OS	2	1	1.3360	0.8361
Q14651	PLS1	70.2	Plastin-1 OS	16	7	1.3360	0.8080
P10619	CTSA	54.4	Lysosomal protective protein OS	28	9	1.3350	0.5503
A0A087X 0R6	SNX12	19.8	Sorting nexin-12 OS	43	6	1.3350	0.7021
Q9NPA8	ENY2	11.5	Transcription and mRNA export factor ENY2 OS	31	2	1.3350	0.8610
Q9GZX9	TWSG1	25	Twisted gastrulation protein homolog 1 OS	7	1	1.3330	0.8165
P30419	NMT1	56.8	Glycylpeptide N-tetradecanoyltransferase 1 OS	7	3	1.3320	0.7066
Q13404	UBE2V1	16.5	Ubiquitin-conjugating enzyme E2 variant 1 OS	51	10	1.3310	0.4769
O75882	ATRN	158. 4	Attractin OS	5	5	1.3300	0.6502
P30048	PRDX3	27.7	Thioredoxin-dependent peroxide reductase, mitochondrial OS	42	10	1.3300	0.5457
Q15369	ELOC	12.5	Elongin-C OS	71	8	1.3280	0.4859
H7BXQ8	ARMC10	20.9	Armadillo repeat-containing protein 10 OS	8	1	1.3270	0.8839
P55212	CASP6	33.3	Caspase-6 OS	3	1	1.3270	0.7110
Q7Z5H4	VN1R5	40.8	Vomeronasal type-1 receptor 5 OS	2	1	1.3270	0.7431
Q9Y315	DERA	35.2	Deoxyribose-phosphate aldolase OS	14	3	1.3260	0.8149
O00391	QSOX1	82.5	Sulfhydryl oxidase 1 OS	27	18	1.3260	0.4894
Q12860	CNTN1	113. 2	Contactin-1 OS	6	4	1.3250	0.8588
O75635	SERPINB7	42.9	Serpin B7 OS	14	4	1.3250	0.7729
Q15102	PAFAH1B3	25.7	Platelet-activating factor acetylhydrolase IB subunit gamma OS	52	6	1.3240	0.7181
Q86UP2	KTN1	156. 2	Kinectin OS	48	54	1.3220	0.4997
Q15046	KARS	68	Lysine--tRNA ligase OS	42	25	1.3220	0.4997
F5H442	TSG101	40.9	Tumor susceptibility gene 101 protein OS	3	1	1.3220	0.8523
Q5JPH6	EARS2	58.7	Probable glutamate--tRNA ligase, mitochondrial OS	2	1	1.3210	0.8819
C9JRL6	EML2	65.5	Echinoderm microtubule-associated protein-like 2 OS	15	6	1.3200	0.7969
E9PGC5	PTPRK	165. 9	Protein-tyrosine-phosphatase OS	14	13	1.3200	0.4920
Q96Q45	TMEM237	45.5	Transmembrane protein 237 OS	3	1	1.3200	0.8474
Q9GZM7	TINAGL1	52.4	Tubulointerstitial nephritis antigen-like OS	51	25	1.3190	0.5091
C9JSK5	PRKAR1B	33.6	cAMP-dependent protein kinase type I-beta regulatory subunit (Fragment) OS	7	2	1.3180	0.8949
Q9H0R4	HDHD2	28.5	Haloacid dehalogenase-like hydrolase domain-containing protein 2 OS	8	2	1.3170	0.8260
P28074	PSMB5	28.5	Proteasome subunit beta type-5 OS	54	17	1.3170	0.5151
P37802	TAGLN2	22.4	Transgelin-2 OS	97	34	1.3170	0.5150
Q52LJ0-1	FAM98B	37.2	Isoform 1 of Protein FAM98B OS	5	1	1.3160	0.8663
A0A5F9U P49	SDF4	40.9	45 kDa calcium-binding protein OS	55	14	1.3150	0.5208
Q9UNS2	COPS3	47.8	COP9 signalosome complex subunit 3 OS	28	6	1.3150	0.7798
P61158	ACTR3	47.3	Actin-related protein 3 OS	72	22	1.3140	0.5205
A0A087W WW9	BCL10	25.1	B-cell lymphoma/leukemia 10 OS	9	1	1.3140	0.8875
H0Y993	DEK hCG_36749	43.2	DEK oncogene (DNA binding), isoform CRA_b OS	18	5	1.3140	0.7444
P43490	NAMPT	55.5	Nicotinamide phosphoribosyltransferase OS	70	34	1.3140	0.5205
Q9HD47	RANGRF	20.4	Ran guanine nucleotide release factor OS	11	2	1.3140	0.8474
Q07021	C1QBP	31.3	Complement component 1 Q subcomponent-binding protein, mitochondrial OS	23	4	1.3130	0.5704
Q9UHD1	CHORDC1	37.5	Cysteine and histidine-rich domain-containing protein 1 OS	53	15	1.3130	0.5234

P49593	PPM1F	49.8	Protein phosphatase 1F OS	14	4	1.3130	0.6531
Q14118	DAG1	97.4	Dystroglycan OS	15	13	1.3120	0.5151
Q96FQ6	S100A16	11.8	Protein S100-A16 OS	78	7	1.3120	0.7290
P13497	BMP1	111.2	Bone morphogenetic protein 1 OS	3	3	1.3110	0.8593
Q9HCC0	MCCC2	61.3	Methylcrotonoyl-CoA carboxylase beta chain, mitochondrial OS	2	1	1.3110	0.6531
A0A0U1RRM4	PTBP1	62.4	Polypyrimidine tract-binding protein 1 OS	44	23	1.3110	0.5293
P20700	LMNB1	66.4	Lamin-B1 OS	56	33	1.3090	0.5348
O95373	IPO7	119.4	Importin-7 OS	10	8	1.3060	0.7431
P22059	OSBP	89.4	Oxysterol-binding protein 1 OS	9	6	1.3060	0.7784
P61106	RAB14	23.9	Ras-related protein Rab-14 OS	33	4	1.3060	0.7784
A0A087WY55	VTA1	31.1	Chromosome 6 open reading frame 55, isoform CRA_b OS	37	7	1.3040	0.6511
P09913	IFIT2	54.6	Interferon-induced protein with tetratricopeptide repeats 2 OS	15	4	1.3040	0.8165
P63279	UBE2I	18	SUMO-conjugating enzyme UBC9 OS	47	7	1.3030	0.6061
B0UXQ0	HLA-A	40.8	HLA class I histocompatibility antigen, A alpha chain OS	42	11	1.3020	0.5511
O14979-2	HNRPDL	33.6	Isoform 2 of Heterogeneous nuclear ribonucleoprotein D-like OS	50	14	1.3020	0.5522
Q7Z6Z7	HUWE1	481.6	E3 ubiquitin-protein ligase HUWE1 OS	2	4	1.3000	0.8958
Q53EL6	PDCD4	51.7	Programmed cell death protein 4 OS	5	1	1.3000	0.8592
Q96KM6	ZNF512B	97.2	Zinc finger protein 512B OS	2	1	1.3000	0.8752
Q12800	TFCP2	57.2	Alpha-globin transcription factor CP2 OS	2	1	1.2990	0.6597
C9JBI3	PSPH	20.7	O-phosphoserine phosphohydrolase (Fragment) OS	62	11	1.2990	0.5740
Q8WUA2	PPIL4	57.2	Peptidyl-prolyl cis-trans isomerase-like 4 OS	15	5	1.2990	0.7903
Q14019	COTL1	15.9	Coactosin-like protein OS	82	15	1.2980	0.5650
O15144	ARPC2	34.3	Actin-related protein 2/3 complex subunit 2 OS	50	14	1.2970	0.5887
P10644	PRKAR1A	43	cAMP-dependent protein kinase type I-alpha regulatory subunit OS	19	6	1.2970	0.7962
B4DXZ6	FXR1	68.3	Fragile X mental retardation syndrome-related protein 1 OS	4	2	1.2960	0.8924
O43390	HNRNPR	70.9	Heterogeneous nuclear ribonucleoprotein R OS	43	29	1.2960	0.5704
P07686	HEXB	63.1	Beta-hexosaminidase subunit beta OS	46	25	1.2940	0.5771
M0QXH4	HAS1	23.6	Hyaluronan synthase 1 OS	4	1	1.2940	0.6531
O14933	UBE2L6	17.8	Ubiquitin/ISG15-conjugating enzyme E2 L6 OS	71	8	1.2940	0.8361
O94760	DDAH1	31.1	N(G),N(G)-dimethylarginine dimethylaminohydrolase 1 OS	28	5	1.2920	0.7534
P20810-6	CAST	84.9	Isoform 6 of Calpastatin OS	40	22	1.2910	0.5881
Q08174	PCDH1	114.7	Protocadherin-1 OS	16	11	1.2890	0.6555
Q99471	PFDN5	17.3	Prefoldin subunit 5 OS	64	8	1.2880	0.6890
O14980	XPO1	123.3	Exportin-1 OS	25	19	1.2870	0.6267
Q8N335	GPD1L	38.4	Glycerol-3-phosphate dehydrogenase 1-like protein OS	2	1	1.2870	0.7107
O60547	GMDS	41.9	GDP-mannose 4,6 dehydratase OS	10	3	1.2860	0.8668
Q9BVG4	PBDC1	26	Protein PBDC1 OS	57	8	1.2860	0.6833
Q16181	SEPT7	50.6	Septin-7 OS	41	17	1.2860	0.6015
Q96FW1	OTUB1	31.3	Ubiquitin thioesterase OTUB1 OS	57	15	1.2860	0.5997
P26038	MSN	67.8	Moesin OS	84	69	1.2840	0.6081
Q13813	SPTAN1	284.4	Spectrin alpha chain, non-erythrocytic 1 OS	38	69	1.2830	0.6106
P05386	RPLP1	11.5	60S acidic ribosomal protein P1 OS	100	6	1.2820	0.6137
Q9UNN5	FAF1	73.9	FAS-associated factor 1 OS	3	1	1.2820	0.9209
A0A0A0MT47	MPHOSPH8	36.1	M-phase phosphoprotein 8 (Fragment) OS	2	1	1.2810	0.9083
Q00534	CDK6	36.9	Cyclin-dependent kinase 6 OS	11	2	1.2800	0.9366

P30041	PRDX6	25	Peroxiredoxin-6 OS	86	23	1.2800	0.6219
Q86V81	ALYREF	26.9	THO complex subunit 4 OS	45	8	1.2800	0.6016
O95359	TACC2	309.2	Transforming acidic coiled-coil-containing protein 2 OS	2	4	1.2800	0.8762
H0YHA7	RPL18	19	60S ribosomal protein L18 (Fragment) OS	20	4	1.2790	0.8051
O00400	SLC33A1	60.9	Acetyl-coenzyme A transporter 1 OS	1	1	1.2790	0.6230
Q13308	PTK7	118.3	Inactive tyrosine-protein kinase 7 OS	23	15	1.2790	0.6280
D6RB92	PSMG4	11.6	Proteasome assembly chaperone 4 OS	34	2	1.2780	0.8906
P05023	ATP1A1	112.8	Sodium/potassium-transporting ATPase subunit alpha-1 OS	20	15	1.2780	0.6310
K7EJL1	AP1M1	40.3	AP-1 complex subunit mu-1 OS	10	3	1.2770	0.8945
P21291	CSRP1	20.6	Cysteine and glycine-rich protein 1 OS	70	10	1.2770	0.6313
Q06481	APLP2	86.9	Amyloid-like protein 2 OS	28	17	1.2760	0.6332
Q9NYQ8	FAT2	479	Protocadherin Fat 2 OS	14	38	1.2760	0.6320
P20933	AGA	37.2	N(4)-(beta-N-acetylglucosaminyl)-L-asparaginase OS	3	1	1.2750	0.8529
Q16666	IFI16	88.2	Gamma-interferon-inducible protein 16 OS	13	7	1.2740	0.7798
B5MCZ3	IL6	21.5	Interleukin-6 OS	15	2	1.2740	0.8649
P12931	SRC	59.8	Proto-oncogene tyrosine-protein kinase Src OS	14	6	1.2740	0.8780
A8MUD9	RPL7	24.4	60S ribosomal protein L7 OS	31	5	1.2730	0.7049
P22061	PCMT1	24.6	Protein-L-isoaspartate(D-aspartate) O-methyltransferase OS	81	13	1.2730	0.6384
O95782	AP2A1	107.5	AP-2 complex subunit alpha-1 OS	15	12	1.2720	0.7056
Q99715	COL12A1	332.9	Collagen alpha-1(XII) chain OS	17	37	1.2720	0.6418
P67775	PPP2CA	35.6	Serine/threonine-protein phosphatase 2A catalytic subunit alpha isoform OS	59	13	1.2720	0.7224
P16152	CBR1	30.4	Carbonyl reductase [NADPH] 1 OS	84	23	1.2710	0.6438
Q9NUG6	PDRG1	15.5	p53 and DNA damage-regulated protein 1 OS	13	1	1.2700	0.9307
O60828	PQBP1	30.5	Polyglutamine-binding protein 1 OS	13	2	1.2700	0.8585
P62273	RPS29	6.7	40S ribosomal protein S29 OS	41	3	1.2690	0.7714
P05198	EIF2S1	36.1	Eukaryotic translation initiation factor 2 subunit 1 OS	70	20	1.2690	0.6500
P26639	TARS	83.4	Threonine--tRNA ligase 1, cytoplasmic OS	63	37	1.2690	0.6502
J3KRX5	RPL17	20.2	60S ribosomal protein L17 (Fragment) OS	27	4	1.2680	0.7868
O14561	NDUFAB1	17.4	Acyl carrier protein, mitochondrial OS	13	2	1.2680	0.9174
P54920	NAPA	33.2	Alpha-soluble NSF attachment protein OS	23	6	1.2680	0.7891
Q9Y508	RNF114	25.7	E3 ubiquitin-protein ligase RNF114 OS	7	2	1.2680	0.8663
H0YC67	RMDN1	17.6	Regulator of microtubule dynamics protein 1 (Fragment) OS	19	3	1.2680	0.8095
E9PLL6	RPL27A	12.2	60S ribosomal protein L27a OS	12	1	1.2670	0.7881
Q7Z406	MYH14	227.7	Myosin-14 OS	6	8	1.2670	0.8605
Q96CX2	KCTD12	35.7	BTB/POZ domain-containing protein KCTD12 OS	63	20	1.2660	0.6913
P51608	MECP2	52.4	Methyl-CpG-binding protein 2 OS	4	1	1.2660	0.8583
Q9UFN0	NIPSNAP3A	28.4	Protein NipSnap homolog 3A OS	10	3	1.2660	0.8938
Q9Y2Z0	SUGT1	41	Protein SGT1 homolog OS	28	7	1.2660	0.7528
H7BXY6	TSPAN14	23.9	Tetraspanin OS	4	1	1.2660	0.8203
E9PKF8	EIF4G2	20.7	Eukaryotic translation initiation factor 4 gamma 2 (Fragment) OS	8	1	1.2650	0.9228
P80217	IFI35	31.5	Interferon-induced 35 kDa protein OS	24	7	1.2650	0.7697
P48147	PREP	80.6	Prolyl endopeptidase OS	26	13	1.2650	0.7586
Q05639	EEF1A2	50.4	Elongation factor 1-alpha 2 OS	48	20	1.2630	0.8344
Q9NWU2	GID8	26.7	Glucose-induced degradation protein 8 homolog OS	11	1	1.2630	0.9343
J3QRU1	YES1	61.3	Tyrosine-protein kinase OS	5	3	1.2630	0.8585

M0QXM4	SLC1A5	39.4	Amino acid transporter OS	17	6	1.2620	0.7431
P32321	DCTD	20	Deoxycytidylate deaminase OS	38	5	1.2620	0.8546
Q9UI30	TRMT112	14.2	Multifunctional methyltransferase subunit TRM112-like protein OS	26	3	1.2620	0.8344
A0A0A0 MRH0	NLE1	48.5	Notchless protein homolog 1 OS	25	6	1.2620	0.8313
A0A494C 114	NRG1	22.7	Pro-neuregulin-1, membrane-bound isoform OS	19	5	1.2620	0.7245
P04083	ANXA1	38.7	Annexin A1 OS	84	42	1.2610	0.6680
Q9GZP8	C19orf33	10.9	Immortalization up-regulated protein OS	34	2	1.2610	0.7969
O94903	PLPBP	30.3	Pyridoxal phosphate homeostasis protein OS	25	6	1.2610	0.7870
Q9Y5X3	SNX5	46.8	Sorting nexin-5 OS	21	6	1.2610	0.8065
P13726	F3	33	Tissue factor OS	19	3	1.2610	0.8742
P41091	EIF2S3	51.1	Eukaryotic translation initiation factor 2 subunit 3 OS	50	22	1.2600	0.6721
A0A2R8Y E10	VPS45	61.1	Vacuolar protein sorting-associated protein 45 OS	3	1	1.2590	0.9240
Q8N684	CPSF7	52	Cleavage and polyadenylation specificity factor subunit 7 OS	7	3	1.2580	0.8398
A8MXP9	MATR3	99.9	Matrin-3 OS	7	5	1.2560	0.6848
Q6ZRP7	QSOX2	77.5	Sulfhydryl oxidase 2 OS	16	7	1.2560	0.7932
H0YBT8	TACC1	50.5	Transforming acidic coiled-coil-containing protein 1 (Fragment) OS	2	1	1.2560	0.9228
P47989	XDH	146. 3	Xanthine dehydrogenase/oxidase OS	1	1	1.2560	0.9403
Q8WVJ2	NUDCD2	17.7	NudC domain-containing protein 2 OS	20	2	1.2550	0.8761
P09211	GSTP1	23.3	Glutathione S-transferase P OS	75	26	1.2540	0.6913
P62633	CNBP	19.5	Cellular nucleic acid-binding protein OS	40	9	1.2530	0.6766
F8W8N3	SKP1	17.9	Cyclin-A/CDK2-associated protein p19 (Fragment) OS	58	6	1.2510	0.8051
P62879	GNB2	37.3	Guanine nucleotide-binding protein G(I)/G(S)/G(T) subunit beta-2 OS	20	5	1.2510	0.8421
Q16836	HADH	34.3	Hydroxyacyl-coenzyme A dehydrogenase, mitochondrial OS	21	5	1.2510	0.7811
P25398	RPS12	14.5	40S ribosomal protein S12 OS	89	16	1.2500	0.7066
K7ELC7	RPL27	16.5	60S ribosomal protein L27 (Fragment) OS	23	2	1.2500	0.8438
P04406	GAPDH	36	Glyceraldehyde-3-phosphate dehydrogenase OS	98	79	1.2500	0.7066
P31949	S100A11	11.7	Protein S100-A11 OS	92	20	1.2490	0.7091
Q13435	SF3B2	100. 2	Splicing factor 3B subunit 2 OS	27	17	1.2490	0.7079
F6T1Q0	PDE12	52.1	2',5'-phosphodiesterase 12 OS	4	1	1.2480	0.8955
Q5VWZ2	LYPLAL1	26.3	Lysophospholipase-like protein 1 OS	13	3	1.2480	0.8795
Q9P2B2	PTGFRN	98.5	Prostaglandin F2 receptor negative regulator OS	6	3	1.2480	0.8987
Q9UBE0	SAE1	38.4	SUMO-activating enzyme subunit 1 OS	32	7	1.2470	0.7521
P34932	HSPA4	94.3	Heat shock 70 kDa protein 4 OS	63	60	1.2460	0.7164
O00231	PSMD11	47.4	26S proteasome non-ATPase regulatory subunit 11 OS	30	11	1.2450	0.7565
O43861	ATP9B	129. 2	Probable phospholipid-transporting ATPase IIB OS	1	1	1.2450	0.9494
P00568	AK1	21.6	Adenylate kinase isoenzyme 1 OS	71	14	1.2430	0.7245
C9JK83	STAMBP	36.1	STAM-binding protein (Fragment) OS	15	3	1.2430	0.8585
O43252	PAPSS1	70.8	Bifunctional 3'-phosphoadenosine 5'-phosphosulfate synthase 1 OS	6	3	1.2420	0.8585
Q96BD5	PHF21A	74.8	PHD finger protein 21A OS	2	1	1.2420	0.8461
Q9HCB6	SPON1	90.9	Spondin-1 OS	1	1	1.2420	0.8260
Q16778	HIST2H2BE	13.9	Histone H2B type 2-E OS	83	20	1.2410	0.7735
P00491	PNP	32.1	Purine nucleoside phosphorylase OS	73	17	1.2410	0.7290
Q5QPQ1	LYPLA2	17.5	Acyl-protein thioesterase 2 (Fragment) OS	31	3	1.2400	0.8821
E9PK91	BCLAF1	100. 3	Bcl-2-associated transcription factor 1 OS	8	5	1.2390	0.8501
Q9Y266	NUDC	38.2	Nuclear migration protein nudC OS	41	19	1.2380	0.7384

Q9UKL0	RCOR1	53.3	REST corepressor 1 OS	8	3	1.2380	0.9221
Q8WVQ1	CANT1	44.8	Soluble calcium-activated nucleotidase 1 OS	7	3	1.2380	0.8906
O00505	KPNA3	57.8	Importin subunit alpha-4 OS	7	3	1.2370	0.8581
Q16204	CCDC6	53.3	Coiled-coil domain-containing protein 6 OS	37	10	1.2360	0.7699
Q15366	PCBP2	38.6	Poly(rC)-binding protein 2 OS	43	13	1.2350	0.7735
O15347	HMGB3	23	High mobility group protein B3 OS	43	7	1.2340	0.7851
P31153	MAT2A	43.6	S-adenosylmethionine synthase isoform type-2 OS	41	16	1.2330	0.7450
O00232	PSMD12	52.9	26S proteasome non-ATPase regulatory subunit 12 OS	14	5	1.2320	0.8979
O15145	ARPC3	20.5	Actin-related protein 2/3 complex subunit 3 OS	38	8	1.2320	0.7798
Q9BXV9	GON7	10.9	EKC/KEOPS complex subunit GON7 OS	18	2	1.2320	0.9126
P16989-2	YBX3	31.9	Isoform 2 of Y-box-binding protein 3 OS	72	12	1.2320	0.7936
A0A087X142	SEPTIN8	49.3	Septin-8 OS	36	11	1.2320	0.7725
P54136	RARS	75.3	Arginine--tRNA ligase, cytoplasmic OS	43	21	1.2310	0.7484
P08572	COL4A2	167.4	Collagen alpha-2(IV) chain OS	8	7	1.2300	0.7798
Q99627	COPS8	23.2	COP9 signalosome complex subunit 8 OS	56	6	1.2300	0.8663
P16930	FAH	46.3	Fumarylacetoacetase OS	46	13	1.2300	0.7798
P30086	PEBP1	21	Phosphatidylethanolamine-binding protein 1 OS	92	23	1.2290	0.7494
P09874	PARP1	113	Poly [ADP-ribose] polymerase 1 OS	55	44	1.2290	0.7494
Q15428	SF3A2	49.2	Splicing factor 3A subunit 2 OS	17	6	1.2290	0.8383
Q15582	TGFBI	74.6	Transforming growth factor-beta-induced protein ig-h3 OS	66	53	1.2290	0.7494
O60763	USO1	107.8	General vesicular transport factor p115 OS	19	11	1.2280	0.8456
Q9H0U4	RAB1B	22.2	Ras-related protein Rab-1B OS	49	9	1.2280	0.7843
P01024	C3	187	Complement C3 OS	67	122	1.2270	0.7551
P35052	GPC1	61.6	Glypican-1 OS	31	12	1.2270	0.7735
P05546	SERPIND1	57	Heparin cofactor 2 OS	1	1	1.2270	0.9001
A8MQB8	FMR1	65.8	Synaptic functional regulator FMR1 OS	2	1	1.2270	0.9484
O43488	AKR7A2	39.6	Aflatoxin B1 aldehyde reductase member 2 OS	27	6	1.2260	0.8663
O43242	PSMD3	60.9	26S proteasome non-ATPase regulatory subunit 3 OS	29	11	1.2250	0.8053
Q12792	TWF1	40.3	Twinfilin-1 OS	26	7	1.2250	0.7886
P13010	XRCC5	82.7	X-ray repair cross-complementing protein 5 OS	76	42	1.2250	0.7627
P13796	LCP1	70.2	Plastin-2 OS	55	32	1.2240	0.7679
A0A087WT45	GRIPAP1	90.8	GRIP1-associated protein 1 OS	12	5	1.2230	0.9083
Q8NDH3	NPEPL1	55.8	Probable aminopeptidase NPEPL1 OS	10	3	1.2230	0.9228
Q5T760	SRSF11	42.3	Serine/arginine-rich-splicing factor 11 (Fragment) OS	2	1	1.2230	0.9228
H0YK63	TRIP4	4.7	Activating signal cointegrator 1 OS	51	1	1.2220	0.9236
P55268	LAMB2	195.9	Laminin subunit beta-2 OS	8	9	1.2220	0.7826
P62195	PSMC5	45.6	26S proteasome regulatory subunit 8 OS	25	6	1.2210	0.8585
E9PLK3	NPEPPS	102.9	Aminopeptidase OS	48	41	1.2210	0.7724
P99999	CYCS	11.7	Cytochrome c OS	79	16	1.2210	0.7724
P60842	EIF4A1	46.1	Eukaryotic initiation factor 4A-I OS	82	30	1.2210	0.7724
C9JZR2	CTNND1	104.8	Catenin delta-1 OS	2	1	1.2200	0.9221
D6RF35	GC	53	Gc-globulin OS	13	10	1.2200	0.7727
P42785	PRCP	55.8	Lysosomal Pro-X carboxypeptidase OS	10	5	1.2200	0.8607
P39019	RPS19	16.1	40S ribosomal protein S19 OS	43	8	1.2190	0.7639
P26368	U2AF2	53.5	Splicing factor U2AF 65 kDa subunit OS	30	10	1.2190	0.8088

Q96PP9	GBP4	73.1	Guanylate-binding protein 4 OS	1	1	1.2180	0.9083
Q13283	G3BP1	52.1	Ras GTPase-activating protein-binding protein 1 OS	68	26	1.2170	0.7784
Q8NDD1	C1orf131	32.6	Uncharacterized protein C1orf131 OS	3	1	1.2170	0.9068
P01889	HLA-B	40.4	HLA class I histocompatibility antigen, B alpha chain OS	35	10	1.2160	0.8051
Q96AE4-2	FUBP1	68.6	Isoform 2 of Far upstream element-binding protein 1 OS	33	16	1.2160	0.7735
Q13442	PDAP1	20.6	28 kDa heat- and acid-stable phosphoprotein OS	54	14	1.2150	0.7798
O75828	CBR3	30.8	Carbonyl reductase [NADPH] 3 OS	35	9	1.2150	0.7969
E9PCY7	HNRNPH1	47.1	Heterogeneous nuclear ribonucleoprotein H OS	24	8	1.2150	0.8058
P49790	NUP153	153.8	Nuclear pore complex protein Nup153 OS	2	2	1.2150	0.9222
O15143	ARPC1B	40.9	Actin-related protein 2/3 complex subunit 1B OS	41	11	1.2130	0.8117
Q8N3L3	TXLNB	76.5	Beta-taxilin OS	1	1	1.2110	0.9436
P32970	CD70	21.1	CD70 antigen OS	20	3	1.2110	0.8260
P56537	EIF6	26.6	Eukaryotic translation initiation factor 6 OS	62	10	1.2110	0.7851
Q16774	GUK1	21.7	Guanylate kinase OS	9	1	1.2110	0.9273
P36952	SERPINB5	42.1	Serpin B5 OS	88	36	1.2110	0.7851
Q10570	CPSF1	160.8	Cleavage and polyadenylation specificity factor subunit 1 OS	2	2	1.2100	0.9449
P49247	RPIA	33.2	Ribose-5-phosphate isomerase OS	16	3	1.2100	0.8855
P00441	SOD1	15.9	Superoxide dismutase [Cu-Zn] OS	91	10	1.2100	0.7872
P61086	UBE2K	22.4	Ubiquitin-conjugating enzyme E2 K OS	30	5	1.2100	0.8588
P43487	RANBP1	23.3	Ran-specific GTPase-activating protein OS	64	12	1.2090	0.7917
Q24JP5	TMEM132A	110	Transmembrane protein 132A OS	18	9	1.2090	0.8219
Q99832	CCT7	59.3	T-complex protein 1 subunit eta OS	77	38	1.2080	0.7927
P00167	CYB5A	15.3	Cytochrome b5 OS	7	1	1.2070	0.9299
P17936	IGFBP3	31.7	Insulin-like growth factor-binding protein 3 OS	44	9	1.2070	0.7965
P13797	PLS3	70.8	Plastin-3 OS	58	36	1.2060	0.7987
Q06203	PPAT	57.4	Amidophosphoribosyltransferase OS	11	5	1.2050	0.9209
D6RAR4	HGFAC	71.4	Hepatocyte growth factor activator OS	2	1	1.2050	0.9299
D6RBZ0	HNRNPAB	35.7	Heterogeneous nuclear ribonucleoprotein A/B OS	45	22	1.2050	0.7992
P50502	ST13	41.3	Hsc70-interacting protein OS	39	16	1.2050	0.7992
A0A087WZF1	LPP	63.3	Lipoma-preferred partner OS	7	2	1.2050	0.9422
Q07960	ARHGAP1	50.4	Rho GTPase-activating protein 1 OS	28	9	1.2040	0.8875
Q9BV19	C1orf50	21.9	Uncharacterized protein C1orf50 OS	11	1	1.2030	0.9704
P62081	RPS7	22.1	40S ribosomal protein S7 OS	39	8	1.2020	0.8181
E9PDU6	CNN3	20.2	Calponin (Fragment) OS	15	2	1.2020	0.9228
F8W6I7	HNRNPA1	33.1	Helix-destabilizing protein OS	68	28	1.2020	0.8058
O14929	HAT1	49.5	Histone acetyltransferase type B catalytic subunit OS	20	7	1.2020	0.8312
P02795	MT2A	6	Metallothionein-2 OS	92	5	1.2020	0.8260
P52306	RAP1GDS1	66.3	Rap1 GTPase-GDP dissociation stimulator 1 OS	4	2	1.2020	0.9343
E9PC52	RBBP7	46.9	Histone-binding protein RBBP7 OS	48	14	1.2000	0.8111
P61326	MAGOH	17.2	Protein mago nashi homolog OS	48	9	1.2000	0.8344
P47755	CAPZA2	32.9	F-actin-capping protein subunit alpha-2 OS	47	10	1.1990	0.8474
P53609	PGGT1B	42.3	Geranylgeranyl transferase type-1 subunit beta OS	2	1	1.1990	0.9397
Q96IU4	ABHD14B	22.3	Protein ABHD14B OS	40	5	1.1990	0.8766
H7C5K4	CCDC80	30.5	Coiled-coil domain-containing protein 80 (Fragment) OS	5	1	1.1980	0.9567
Q96GQ7	DDX27	89.8	Probable ATP-dependent RNA helicase DDX27 OS	4	1	1.1980	0.9477

P43686	PSMC4	47.3	26S proteasome regulatory subunit 6B OS	38	12	1.1970	0.8344
Q4VC31	CCDC58	16.6	Coiled-coil domain-containing protein 58 OS	76	11	1.1970	0.8344
Q15370	ELOB	13.1	Elongin-B OS	98	9	1.1970	0.8407
Q96KB5	PBK	36.1	Lymphokine-activated killer T-cell-originated protein kinase OS	32	7	1.1970	0.8906
Q09161	NCBP1	91.8	Nuclear cap-binding protein subunit 1 OS	6	3	1.1970	0.9025
Q9Y237	PIN4	13.8	Peptidyl-prolyl cis-trans isomerase NIMA-interacting 4 OS	51	6	1.1970	0.8373
P35237	SERPINB6	42.6	Serpin B6 OS	71	27	1.1960	0.8165
O95881	TXNDC12	19.2	Thioredoxin domain-containing protein 12 OS	57	6	1.1960	0.8963
P08865	RPSA	32.8	40S ribosomal protein SA OS	69	21	1.1950	0.8190
P16562	CRISP2	27.2	Cysteine-rich secretory protein 2 OS	14	1	1.1950	0.9632
Q13751	LAMB3	129.5	Laminin subunit beta-3 OS	79	86	1.1950	0.8214
Q8WXC6	COPS9	6.2	COP9 signalosome complex subunit 9 OS	35	1	1.1940	0.9778
P33176	KIF5B	109.6	Kinesin-1 heavy chain OS	11	8	1.1940	0.8313
P49588	AARS	106.7	Alanine--tRNA ligase, cytoplasmic OS	19	13	1.1920	0.8476
A0A087WVQ6	CLTC	191.9	Clathrin heavy chain OS	39	57	1.1920	0.8265
P78417	GSTO1	27.5	Glutathione S-transferase omega-1 OS	67	18	1.1920	0.8265
P56192	MARS	101.1	Methionine--tRNA ligase, cytoplasmic OS	9	6	1.1920	0.8165
Q9BPZ3	PAIP2	15	Polyadenylate-binding protein-interacting protein 2 OS	20	2	1.1920	0.9352
F6TLX2	GLOD4	54.7	Glyoxalase domain-containing protein 4 OS	40	20	1.1900	0.8313
P04899	GNAI2	40.4	Guanine nucleotide-binding protein G(i) subunit alpha-2 OS	13	3	1.1900	0.9403
A0A0G2JIW1	HSPA1B	70.1	Heat shock 70 kDa protein 1B OS	71	65	1.1900	0.8317
Q9C005	DPY30	11.2	Protein dpy-30 homolog OS	77	4	1.1900	0.9240
H3BQZ5	C15orf40	7.5	UPF0235 protein C15orf40 OS	24	1	1.1890	0.9484
O00487	PSMD14	34.6	26S proteasome non-ATPase regulatory subunit 14 OS	33	7	1.1880	0.8914
Q92905	COPS5	37.6	COP9 signalosome complex subunit 5 OS	28	6	1.1880	0.8824
Q16394	EXT1	86.2	Exostosin-1 OS	7	4	1.1880	0.9236
Q96QU8	XPO6	128.8	Exportin-6 OS	1	1	1.1880	0.8585
P55327	TPD52	24.3	Tumor protein D52 OS	43	6	1.1880	0.8581
Q16531	DDB1	126.9	DNA damage-binding protein 1 OS	42	37	1.1870	0.8344
Q12931	TRAP1	80.1	Heat shock protein 75 kDa, mitochondrial OS	10	5	1.1870	0.9057
Q15181	PPA1	32.6	Inorganic pyrophosphatase OS	90	21	1.1870	0.8361
Q8TBC4	UBA3	51.8	NEDD8-activating enzyme E1 catalytic subunit OS	13	3	1.1870	0.9349
Q8NHP8	PLBD2	65.4	Putative phospholipase B-like 2 OS	4	2	1.1870	0.9299
Q5W0H4	TPT1	21.5	Translationally-controlled tumor protein OS	71	16	1.1870	0.8344
Q9Y281	CFL2	18.7	Cofilin-2 OS	57	16	1.1860	0.9165
Q9UNZ2	NSFL1C	40.5	NSFL1 cofactor p47 OS	62	24	1.1860	0.8363
P20290	BTF3	22.2	Transcription factor BTF3 OS	41	7	1.1850	0.8369
P50552	VASP	39.8	Vasodilator-stimulated phosphoprotein OS	32	9	1.1850	0.8632
P22234	PAICS	47	Multifunctional protein ADE2 OS	50	20	1.1840	0.8407
Q02790	FKBP4	51.8	Peptidyl-prolyl cis-trans isomerase FKBP4 OS	81	38	1.1840	0.8426
Q15029	EFTUD2	109.4	116 kDa U5 small nuclear ribonucleoprotein component OS	23	15	1.1830	0.8585
P06132	UROD	40.8	Uroporphyrinogen decarboxylase OS	43	9	1.1830	0.8983
Q9C0C2	TNKS1BP1	181.7	182 kDa tankyrase-1-binding protein OS	24	27	1.1820	0.8359
Q86XP3	DDX42	102.9	ATP-dependent RNA helicase DDX42 OS	7	5	1.1820	0.9221
Q9UNE7	STUB1	34.8	E3 ubiquitin-protein ligase CHIP OS	9	2	1.1820	0.9221

P41252	IARS	144.4	Isoleucine--tRNA ligase, cytoplasmic OS	22	22	1.1820	0.8461
A0A6I8PL42	LARS1	129.1	Leucyl-tRNA synthetase OS	14	10	1.1820	0.8607
P62942	FKBP1A	11.9	Peptidyl-prolyl cis-trans isomerase FKBP1A OS	93	13	1.1820	0.8461
P61247	RPS3A	29.9	40S ribosomal protein S3a OS	47	10	1.1810	0.8592
O75223	GGCT	21	Gamma-glutamylcyclotransferase OS	93	18	1.1810	0.8495
Q8N1G4	LRRC47	63.4	Leucine-rich repeat-containing protein 47 OS	22	9	1.1810	0.8670
Q96C19	EFHD2	26.7	EF-hand domain-containing protein D2 OS	40	11	1.1800	0.8501
H0YEW1	LRRK1	73	Leucine-rich repeat serine/threonine-protein kinase 1 (Fragment) OS	1	1	1.1800	0.8663
Q9NPF4	OSGEP	36.4	Probable tRNA N6-adenosine threonylcarbamoyltransferase OS	21	5	1.1800	0.9228
P21926	CD9	25.4	CD9 antigen OS	15	3	1.1790	0.9335
Q93077	HIST1H2AC	14.1	Histone H2A type 1-C OS	63	15	1.1790	0.9045
Q86WQ0	NR2C2AP	15.9	Nuclear receptor 2C2-associated protein OS	35	3	1.1790	0.9449
Q9H6Z4	RANBP3	60.2	Ran-binding protein 3 OS	23	8	1.1790	0.8752
Q16186	ADRM1	42.1	Proteasomal ubiquitin receptor ADRM1 OS	13	3	1.1780	0.9449
Q99584	S100A13	11.5	Protein S100-A13 OS	57	4	1.1780	0.8607
P78527	PRKDC	468.8	DNA-dependent protein kinase catalytic subunit OS	2	7	1.1760	0.8585
P08238	HSP90AB1	83.2	Heat shock protein HSP 90-beta OS	80	105	1.1760	0.8585
Q2L6G2	HLA-B	40.5	HLA class I histocompatibility antigen B alpha chain OS	33	11	1.1760	0.8632
Q96C86	DCPS	38.6	m7GpppX diphosphatase OS	20	6	1.1760	0.8764
P49189	ALDH9A1	53.8	4-trimethylaminobutyraldehyde dehydrogenase OS	26	9	1.1750	0.8777
Q96HQ2	CDKN2AIPNL	13.2	CDKN2AIP N-terminal-like protein OS	5	1	1.1750	0.9422
Q92896	GLG1	134.5	Golgi apparatus protein 1 OS	7	7	1.1750	0.8955
Q16787	LAMA3	366.4	Laminin subunit alpha-3 OS	28	88	1.1750	0.8585
P40763	STAT3	88	Signal transducer and activator of transcription 3 OS	14	7	1.1750	0.9383
P23396	RPS3	26.7	40S ribosomal protein S3 OS	56	8	1.1740	0.9136
F8WJN3	CPSF6	52.2	Cleavage and polyadenylation specificity factor subunit 6 OS	9	2	1.1740	0.9463
Q96HY6	DDRGK1	35.6	DDRGK domain-containing protein 1 OS	8	2	1.1740	0.9299
Q53H96	PYCR3	28.6	Pyrroline-5-carboxylate reductase 3 OS	9	2	1.1740	0.9463
Q9Y657	SPIN1	29.6	Spindlin-1 OS	5	1	1.1740	0.9403
P0DP25	CALM3	16.8	Calmodulin-3 OS	72	9	1.1730	0.8605
P52597	HNRNPF	45.6	Heterogeneous nuclear ribonucleoprotein F OS	55	16	1.1730	0.8605
O94826	TOMM70	67.4	Mitochondrial import receptor subunit TOM70 OS	13	7	1.1730	0.9343
O43143	DHX15	90.9	Pre-mRNA-splicing factor ATP-dependent RNA helicase DHX15 OS	19	12	1.1730	0.8780
B1ALA9	PRPS1	31.4	Ribose-phosphate pyrophosphokinase 1 OS	29	5	1.1730	0.9228
Q9NPR2	SEMA4B	92.7	Semaphorin-4B OS	16	8	1.1730	0.8893
Q03393	PTS	16.4	6-pyruvoyl tetrahydrobiopterin synthase OS	6	1	1.1720	0.9484
P47895	ALDH1A3	56.1	Aldehyde dehydrogenase family 1 member A3 OS	65	31	1.1720	0.8607
Q9H4M9	EHD1	60.6	EH domain-containing protein 1 OS	32	11	1.1720	0.8736
P62873	GNB1	37.4	Guanine nucleotide-binding protein G(I)/G(S)/G(T) subunit beta-1 OS	22	5	1.1720	0.8874
Q96CV9	OPTN	65.9	Optineurin OS	5	2	1.1720	0.9406
P62906	RPL10A	24.8	60S ribosomal protein L10a OS	42	12	1.1710	0.8634
A0A3B3IUC4	GLA	53.2	Alpha-galactosidase OS	15	8	1.1710	0.8681
M0R165	EPS15L1	83.4	Epidermal growth factor receptor substrate 15-like 1 OS	6	3	1.1710	0.9477
Q9Y5K6	CD2AP	71.4	CD2-associated protein OS	21	9	1.1700	0.9228
Q96AT9	RPE	24.9	Ribulose-phosphate 3-epimerase OS	14	3	1.1700	0.8765

P62314	SNRPD1	13.3	Small nuclear ribonucleoprotein Sm D1 OS	34	4	1.1700	0.9228
Q9NZV1	CRIM1	113.7	Cysteine-rich motor neuron 1 protein OS	7	5	1.1690	0.9069
P41227	NAA10	26.4	N-alpha-acetyltransferase 10 OS	29	5	1.1690	0.8860
O75629	CREG1	24.1	Protein CREG1 OS	15	2	1.1690	0.8874
Q49AN9	SNRPG	7.1	Small nuclear ribonucleoprotein G OS	31	2	1.1690	0.8906
U3KQB0	POLR2F	13.9	DNA-directed RNA polymerase II subunit F OS	15	2	1.1680	0.9484
P54105	CLNS1A	26.2	Methylosome subunit pICln OS	28	3	1.1680	0.9299
Q8WYA6	CTNNBL1	65.1	Beta-catenin-like protein 1 OS	9	3	1.1670	0.9412
P09960	LTA4H	69.2	Leukotriene A-4 hydrolase OS	68	36	1.1670	0.8762
P55290	CDH13	78.2	Cadherin-13 OS	14	8	1.1660	0.8903
Q9H773	DCTPP1	18.7	dCTP pyrophosphatase 1 OS	71	8	1.1660	0.8766
P02751	POLR2F	272.2	Fibronectin OS	33	64	1.1660	0.8766
Q96P70	IPO9	115.9	Importin-9 OS	10	6	1.1660	0.9494
Q9UKY7	CDV3	27.3	Protein CDV3 homolog OS	64	12	1.1660	0.8770
P23526	AHCY	47.7	Adenosylhomocysteinase OS	60	33	1.1650	0.8779
P20042	EIF2S2	38.4	Eukaryotic translation initiation factor 2 subunit 2 OS	29	10	1.1650	0.8960
Q13263	TRIM28	88.5	Transcription intermediary factor 1-beta OS	51	29	1.1650	0.8801
P30085	CMPK1	22.2	UMP-CMP kinase OS	73	14	1.1650	0.8821
O15031	PLXNB2	205	Plexin-B2 OS	7	9	1.1640	0.9221
O75326	SEMA7A	74.8	Semaphorin-7A OS	6	4	1.1640	0.9396
P05067	APP	86.9	Amyloid-beta precursor protein OS	22	11	1.1630	0.8824
Q9Y3C6	PPIL1	18.2	Peptidyl-prolyl cis-trans isomerase-like 1 OS	39	7	1.1630	0.8961
O15212	PFDN6	14.6	Prefoldin subunit 6 OS	83	11	1.1630	0.8839
A0A3B3ITT5	RPL29	18.5	60S ribosomal protein L29 OS	17	4	1.1620	0.9065
P22626	HNRNPA2B1	37.4	Heterogeneous nuclear ribonucleoproteins A2/B1 OS	82	48	1.1620	0.8864
Q460N5-1	PARP14	193.6	Isoform 1 of Protein mono-ADP-ribosyltransferase PARP14 OS	1	1	1.1620	0.9593
Q9NTK5	OLA1	44.7	Obg-like ATPase 1 OS	47	16	1.1620	0.8855
P46940	IQGAP1	189.1	Ras GTPase-activating-like protein IQGAP1 OS	39	55	1.1620	0.8875
Q01082	SPTBN1	274.4	Spectrin beta chain, non-erythrocytic 1 OS	31	62	1.1620	0.8867
Q9Y4E8	USP15	112.3	Ubiquitin carboxyl-terminal hydrolase 15 OS	8	6	1.1610	0.9240
O95400	CD2BP2	37.6	CD2 antigen cytoplasmic tail-binding protein 2 OS	14	4	1.1600	0.9449
P00533	EGFR	134.2	Epidermal growth factor receptor OS	30	24	1.1600	0.8903
F5GY03	SPARC	17.5	Osteonectin (Fragment) OS	11	1	1.1600	0.9449
B4DDF4	CNN2	32.6	Calponin OS	44	11	1.1590	0.8914
P31946-2	YWHAB	27.8	Isoform Short of 14-3-3 protein beta/alpha OS	85	30	1.1590	0.8914
Q04917	YWHAH	28.2	14-3-3 protein eta OS	72	21	1.1580	0.8944
Q9HC35	EML4	108.8	Echinoderm microtubule-associated protein-like 4 OS	3	2	1.1580	0.8945
P62491	RAB11A	24.4	Ras-related protein Rab-11A OS	40	8	1.1580	0.8893
P18206	VCL	123.7	Vinculin OS	64	65	1.1580	0.8932
Q08945	SSRP1	81	FACT complex subunit SSRP1 OS	28	17	1.1570	0.9017
O00233	PSMD9	24.7	26S proteasome non-ATPase regulatory subunit 9 OS	44	7	1.1560	0.9059
F8VRL4	NUDT4	11	Diphosphoinositol polyphosphate phosphohydrolase 2 (Fragment) OS	12	1	1.1560	0.9073
A0A3B3IUA7	TRIM25	51	E3 ubiquitin/ISG15 ligase TRIM25 OS	20	6	1.1560	0.9421
O43396	TXNL1	32.2	Thioredoxin-like protein 1 OS	61	13	1.1560	0.8987
P55036	PSMD4	40.7	26S proteasome non-ATPase regulatory subunit 4 OS	14	5	1.1550	0.9191

Q13185	CBX3	20.8	Chromobox protein homolog 3 OS	80	17	1.1550	0.9018
O60888	CUTA	19.1	Protein CutA OS	41	6	1.1550	0.9219
P11413	G6PD	59.2	Glucose-6-phosphate 1-dehydrogenase OS	70	35	1.1530	0.9059
O75369	FLNB	278	Filamin-B OS	48	103	1.1520	0.9070
Q92973-2	TNPO1	101.2	Isoform 2 of Transportin-1 OS	12	8	1.1520	0.9083
D6RH06	PDLIM7	30.5	PDZ and LIM domain protein 7 (Fragment) OS	20	4	1.1520	0.9403
Q9NQG5	RPRD1B	36.9	Regulation of nuclear pre-mRNA domain-containing protein 1B OS	50	10	1.1520	0.8977
P16144-3	ITGB4	200.6	Isoform Beta-4B of Integrin beta-4 OS	23	26	1.1510	0.8961
Q9NXA8	SIRT5	33.9	NAD-dependent protein deacylase sirtuin-5, mitochondrial OS	4	1	1.1510	0.9835
Q96HE7	ERO1L	54.4	ERO1-like protein alpha OS	12	4	1.1500	0.9075
O76003	GLRX3	37.4	Glutaredoxin-3 OS	64	18	1.1500	0.9083
Q12906	ILF3	95.3	Interleukin enhancer-binding factor 3 OS	35	22	1.1500	0.9134
Q03252	LMNB2	69.9	Lamin-B2 OS	45	31	1.1500	0.9143
P46926	GNPDA1	32.6	Glucosamine-6-phosphate isomerase 1 OS	63	16	1.1490	0.9174
P24592	IGFBP6	25.3	Insulin-like growth factor-binding protein 6 OS	56	11	1.1490	0.9161
E9PHY0	ACP2	41	Lysosomal acid phosphatase OS	6	2	1.1490	0.9640
Q14134	TRIM29	65.8	Tripartite motif-containing protein 29 OS	21	13	1.1490	0.9068
Q14011	CIRBP	18.6	Cold-inducible RNA-binding protein OS	58	5	1.1480	0.9422
P05556	ITGB1	88.4	Integrin beta-1 OS	17	11	1.1480	0.9228
P46013	MKI67	358.5	Proliferation marker protein Ki-67 OS	7	13	1.1480	0.9174
O76054	SEC14L2	46.1	SEC14-like protein 2 OS	30	7	1.1480	0.9075
O60841	EIF5B	138.7	Eukaryotic translation initiation factor 5B OS	20	16	1.1470	0.9202
P11216	PYGB	96.6	Glycogen phosphorylase, brain form OS	62	48	1.1470	0.9202
P50395	GDI2	50.6	Rab GDP dissociation inhibitor beta OS	77	42	1.1470	0.9202
Q13641	TPBG	46	Trophoblast glycoprotein OS	2	1	1.1470	0.9742
Q13561	DCTN2	44.2	Dynactin subunit 2 OS	51	16	1.1460	0.9209
Q9BY44	EIF2A	64.9	Eukaryotic translation initiation factor 2A OS	43	17	1.1460	0.9221
Q9Y3C8	UFC1	19.4	Ubiquitin-fold modifier-conjugating enzyme 1 OS	10	2	1.1460	0.9536
P12429	ANXA3	36.4	Annexin A3 OS	35	9	1.1450	0.9234
Q92499	DDX1	82.4	ATP-dependent RNA helicase DDX1 OS	36	22	1.1450	0.9221
P49736	MCM2	101.8	DNA replication licensing factor MCM2 OS	27	16	1.1450	0.9240
O00429	DNM1L	81.8	Dynamin-1-like protein OS	20	13	1.1450	0.9083
P12081	HARS	57.4	Histidine--tRNA ligase, cytoplasmic OS	34	15	1.1450	0.9221
P25788	PSMA3	28.4	Proteasome subunit alpha type-3 OS	66	17	1.1450	0.9222
Q9UQE7	SMC3	141.5	Structural maintenance of chromosomes protein 3 OS	8	9	1.1450	0.9365
P45974	USP5	95.7	Ubiquitin carboxyl-terminal hydrolase 5 OS	29	14	1.1440	0.9083
Q9UDY4	DNAJB4	37.8	DnaJ homolog subfamily B member 4 OS	8	3	1.1430	0.9282
P48739	PITPNB	31.5	Phosphatidylinositol transfer protein beta isoform OS	46	10	1.1420	0.9228
Q9Y316	MEMO1	33.7	Protein MEMO1 OS	7	1	1.1420	0.9739
Q9UK45	LSM7	11.6	U6 snRNA-associated Sm-like protein LSm7 OS	78	5	1.1420	0.9388
A0A1B0GV23	CTSD	43.8	Cathepsin D OS	65	40	1.1410	0.9236
A0A3B3IRQ9	ADAR	128.3	Double-stranded RNA-specific adenosine deaminase (Fragment) OS	5	5	1.1410	0.9551
Q99598	TSNAX	33.1	Translin-associated protein X OS	44	8	1.1410	0.9228
P04632	CAPNS1	28.3	Calpain small subunit 1 OS	42	10	1.1400	0.9240
O00625	PIR	32.1	Pirin OS	33	6	1.1400	0.9449

Q92734	TFG	43.4	Protein TFG OS	17	6	1.1400	0.9299
P62136	PPP1CA	37.5	Serine/threonine-protein phosphatase PP1-alpha catalytic subunit OS	47	14	1.1400	0.9240
P49327	FASN	273.3	Fatty acid synthase OS	42	81	1.1370	0.9299
Q9P258	RCC2	56	Protein RCC2 OS	58	26	1.1370	0.9299
D6RHI9	RNASET2	29.1	Ribonuclease T2 (Fragment) OS	30	6	1.1370	0.9371
P54578	USP14	56	Ubiquitin carboxyl-terminal hydrolase 14 OS	47	17	1.1370	0.9299
P39687	ANP32A	28.6	Acidic leucine-rich nuclear phosphoprotein 32 family member A OS	59	22	1.1360	0.9299
Q9UHY7	ENOPH1	28.9	Enolase-phosphatase E1 OS	18	4	1.1360	0.9536
P62310	LSM3	11.8	U6 snRNA-associated Sm-like protein LSm3 OS	43	5	1.1360	0.9299
A0A0G2JLB3	GBA	59.6	Glucosylceramidase OS	12	3	1.1350	0.9728
P52292	KPNA2	57.8	Importin subunit alpha-1 OS	21	9	1.1350	0.9449
Q8WXF1	PSPC1	58.7	Paraspeckle component 1 OS	16	4	1.1350	0.9678
Q14563	SEMA3A	88.8	Semaphorin-3A OS	9	4	1.1350	0.9406
P06865	HEXA	60.7	Beta-hexosaminidase subunit alpha OS	34	11	1.1340	0.9240
P22314-2	UBA1	113.7	Isoform 2 of Ubiquitin-like modifier-activating enzyme 1 OS	69	67	1.1340	0.9352
Q99538	LGMN	49.4	Legumain OS	24	7	1.1340	0.9299
P28065	PSMB9	23.3	Proteasome subunit beta type-9 OS	62	10	1.1340	0.9345
P63104	YWHAZ	27.7	14-3-3 protein zeta/delta OS	90	32	1.1330	0.9370
O00303	EIF3F	37.5	Eukaryotic translation initiation factor 3 subunit F OS	59	14	1.1330	0.9396
A6NHT5	HMX3	37.8	Homeobox protein HMX3 OS	4	1	1.1330	0.9626
P78406	RAE1	40.9	mRNA export factor OS	26	7	1.1330	0.9284
O14950	MYL12B	19.8	Myosin regulatory light chain 12B OS	34	4	1.1330	0.9449
P20340	RAB6A	23.6	Ras-related protein Rab-6A OS	13	2	1.1330	0.9774
P26641	EEF1G	50.1	Elongation factor 1-gamma OS	67	34	1.1320	0.9403
O43660	PLRG1	57.2	Pleiotropic regulator 1 OS	28	9	1.1320	0.9299
P63244	RACK1	35.1	Receptor of activated protein C kinase 1 OS	86	27	1.1320	0.9400
A0A087WV05	N/A	12.7	Uncharacterized protein OS	13	1	1.1320	0.9491
Q2TAY7	SMU1	57.5	WD40 repeat-containing protein SMU1 OS	2	1	1.1320	0.9594
P27695	APEX1	35.5	DNA-(apurinic or apyrimidinic site) endonuclease OS	75	21	1.1310	0.9412
P15586	GNS	62	N-acetylglucosamine-6-sulfatase OS	26	11	1.1310	0.9422
F5GZ28	LIG1	93.9	DNA ligase OS	2	1	1.1300	0.9803
A0A087WXM8	BCAM	63.7	Basal cell adhesion molecule OS	45	19	1.1290	0.9449
P68104	EEF1A1	50.1	Elongation factor 1-alpha 1 OS	78	51	1.1290	0.9450
Q9Y5S9	RBM8A	19.9	RNA-binding protein 8A OS	51	3	1.1290	0.9449
P53634	CTSC	51.8	Dipeptidyl peptidase 1 OS	44	17	1.1280	0.9470
P09104	ENO2	47.2	Gamma-enolase OS	36	14	1.1280	0.9462
O14818	PSMA7	27.9	Proteasome subunit alpha type-7 OS	77	26	1.1280	0.9463
H0YDU8	PPP5C	55.2	Serine/threonine-protein phosphatase (Fragment) OS	33	12	1.1280	0.9494
G3XAL9	SLC12A2	124.5	Solute carrier family 12 (Sodium/potassium/chloride transporters), member 2, isoform CRA_a OS	1	1	1.1280	0.9741
Q9BPX5	ARPC5L	16.9	Actin-related protein 2/3 complex subunit 5-like protein OS	8	1	1.1270	0.9660
P01008	SERPINC1	52.6	Antithrombin-III OS	19	10	1.1270	0.9470
Q13740	ALCAM	65.1	CD166 antigen OS	53	25	1.1270	0.9470
P07305	H1F0	20.9	Histone H1.0 OS	20	3	1.1270	0.9299
P35613-2	BSG	29.2	Isoform 2 of Basigin OS	17	3	1.1270	0.9517
Q00796	SORD	38.3	Sorbitol dehydrogenase OS	75	22	1.1270	0.9476

Q86TU7	SETD3	67.2	Actin-histidine N-methyltransferase OS	2	1	1.1260	0.9835
P02649	APOE	36.1	Apolipoprotein E OS	5	2	1.1260	0.9645
P00367	GLUD1	61.4	Glutamate dehydrogenase 1, mitochondrial OS	42	18	1.1260	0.9477
E9PKE3	HSPA8	68.8	Heat shock cognate 71 kDa protein OS	68	71	1.1260	0.9479
A0A3B3ITJ4	HNRNPL	59.2	Heterogeneous nuclear ribonucleoprotein L (Fragment) OS	43	14	1.1260	0.9477
P08476	INHBA	47.4	Inhibin beta A chain OS	51	25	1.1250	0.9484
Q13753	LAMC2	130.9	Laminin subunit gamma-2 OS	66	80	1.1250	0.9484
P12270	TPR	267.1	Nucleoprotein TPR OS	28	46	1.1250	0.9484
Q8TD16	BICD2	93.5	Protein bicaudal D homolog 2 OS	8	4	1.1250	0.9758
P21583	KITLG	30.9	Kit ligand OS	3	1	1.1240	0.9798
O60610	DIAPH1	141.3	Protein diaphanous homolog 1 OS	16	12	1.1240	0.9449
Q96QK1	VPS35	91.6	Vacuolar protein sorting-associated protein 35 OS	21	14	1.1240	0.9498
P10155	TROVE2	60.6	60 kDa SS-A/Ro ribonucleoprotein OS	16	6	1.1230	0.9547
P35221	CTNNA1	100	Catenin alpha-1 OS	54	37	1.1230	0.9511
P12259	F5	251.5	Coagulation factor V OS	1	1	1.1230	0.9976
Q9NVS9	PNPO	30	Pyridoxine-5'-phosphate oxidase OS	44	6	1.1230	0.9536
P37108	SRP14	14.6	Signal recognition particle 14 kDa protein OS	50	6	1.1230	0.9494
Q5XPI4	RNF123	148.4	E3 ubiquitin-protein ligase RNF123 OS	1	2	1.1220	0.9803
Q9BX68	HINT2	17.2	Histidine triad nucleotide-binding protein 2, mitochondrial OS	29	3	1.1220	0.9749
Q92841-1	DDX17	72.3	Isoform 2 of Probable ATP-dependent RNA helicase DDX17 OS	32	15	1.1220	0.9463
Q15691	MAPRE1	30	Microtubule-associated protein RP/EB family member 1 OS	88	23	1.1220	0.9534
Q99497	PARK7	19.9	Parkinson disease protein 7 OS	89	16	1.1220	0.9526
C9JH92	CRYZ	21.9	Quinone oxidoreductase (Fragment) OS	34	5	1.1220	0.9480
P17987	TCP1	60.3	T-complex protein 1 subunit alpha OS	79	43	1.1220	0.9536
P07237	P4HB	57.1	Protein disulfide-isomerase OS	75	35	1.1210	0.9536
O00299	CLIC1	26.9	Chloride intracellular channel protein 1 OS	96	34	1.1200	0.9536
P25205	MCM3	90.9	DNA replication licensing factor MCM3 OS	19	11	1.1200	0.9592
E9PGM1	EIF4G1	166.6	Eukaryotic translation initiation factor 4 gamma 1 OS	25	26	1.1200	0.9536
P50897	PPT1	34.2	Palmitoyl-protein thioesterase 1 OS	39	7	1.1200	0.9422
O76061	STC2	33.2	Stanniocalcin-2 OS	57	15	1.1200	0.9536
G5EA09	SDCBP	34.8	Syndecan binding protein (Syntenin), isoform CRA_a OS	11	2	1.1200	0.9765
P06493	CDK1	34.1	Cyclin-dependent kinase 1 OS	29	6	1.1190	0.9751
F8VSC5	SCYL2	77	SCY1-like protein 2 (Fragment) OS	3	1	1.1190	0.9835
Q9Y2H0-1	DLGAP4	107.5	Isoform 2 of Disks large-associated protein 4 OS	1	1	1.1180	0.9822
P30050	RPL12	17.8	60S ribosomal protein L12 OS	61	8	1.1170	0.9593
O43633	CHMP2A	25.1	Charged multivesicular body protein 2a OS	7	2	1.1170	0.9976
A0A1W2PPZ5	TCEA1	33.9	Transcription elongation factor A protein 1 OS	58	14	1.1170	0.9536
Q5TFE4	NT5DC1	51.8	5'-nucleotidase domain-containing protein 1 OS	43	12	1.1160	0.9637
P60981	DSTN	18.5	Destrin OS	66	17	1.1160	0.9633
P48723	HSPA13	51.9	Heat shock 70 kDa protein 13 OS	5	2	1.1160	0.9835
Q2TB90	HKDC1	102.5	Hexokinase HKDC1 OS	3	4	1.1160	0.9626
P55058	PLTP	54.7	Phospholipid transfer protein OS	16	5	1.1160	0.9477
Q92520	FAM3C	24.7	Protein FAM3C OS	39	6	1.1160	0.9640
A0A182DWF2	TXNRD2	53.5	Thioredoxin-disulfide reductase OS	14	3	1.1160	0.9835
P15121	AKR1B1	35.8	Aldo-keto reductase family 1 member B1 OS	76	21	1.1150	0.9644

Q14974	KPNB1	97.1	Importin subunit beta-1 OS	39	29	1.1150	0.9640
P55263	ADK	40.5	Adenosine kinase OS	45	14	1.1140	0.9660
P52907	CAPZA1	32.9	F-actin-capping protein subunit alpha-1 OS	51	12	1.1140	0.9645
Q9Y285	FARSA	57.5	Phenylalanine--tRNA ligase alpha subunit OS	23	6	1.1140	0.9536
Q15365	PCBP1	37.5	Poly(rC)-binding protein 1 OS	79	22	1.1140	0.9663
P31947	SFN	27.8	14-3-3 protein sigma OS	94	29	1.1130	0.9678
Q9H3K6	BOLA2B	10.1	BolA-like protein 2 OS	85	7	1.1130	0.9567
Q16777	HIST2H2AC	14	Histone H2A type 2-C OS	71	16	1.1130	0.9687
P49006	MARCKSL1	19.5	MARCKS-related protein OS	4	1	1.1130	0.9835
P09758	TACSTD2	35.7	Tumor-associated calcium signal transducer 2 OS	29	10	1.1130	0.9687
Q96KP4	CNDP2	52.8	Cytosolic non-specific dipeptidase OS	59	30	1.1120	0.9708
P49321	NASP	85.2	Nuclear autoantigenic sperm protein OS	33	17	1.1120	0.9697
P07996	THBS1	129.3	Thrombospondin-1 OS	54	76	1.1120	0.9720
P23528	CFL1	18.5	Cofilin-1 OS	99	30	1.1110	0.9729
Q06830	PRDX1	22.1	Peroxiredoxin-1 OS	95	33	1.1110	0.9734
Q01813	PFKP	85.5	ATP-dependent 6-phosphofructokinase, platelet type OS	33	23	1.1100	0.9728
Q9NSD9	FARSB	66.1	Phenylalanine--tRNA ligase beta subunit OS	33	16	1.1100	0.9749
P49368	CCT3	60.5	T-complex protein 1 subunit gamma OS	70	43	1.1100	0.9739
B8ZZT4	VAMP8	11.2	Vesicle-associated membrane protein 8 OS	19	1	1.1100	0.9835
O75348	ATP6V1G1	13.7	V-type proton ATPase subunit G 1 OS	56	4	1.1100	0.9749
P00918	CA2	29.2	Carbonic anhydrase 2 OS	68	19	1.1090	0.9749
Q9H488	POFUT1	43.9	GDP-fucose protein O-fucosyltransferase 1 OS	20	5	1.1090	0.9739
P26583	HMGB2	24	High mobility group protein B2 OS	46	13	1.1090	0.9749
Q9Y617	PSAT1	40.4	Phosphoserine aminotransferase OS	86	37	1.1090	0.9749
P13667	PDIA4	72.9	Protein disulfide-isomerase A4 OS	37	22	1.1090	0.9749
G3V5L1	MAX	14	Protein max OS	12	1	1.1090	0.9835
Q96HC4	PDLIM5	63.9	PDZ and LIM domain protein 5 OS	18	7	1.1080	0.9749
P27694	RPA1	68.1	Replication protein A 70 kDa DNA-binding subunit OS	28	9	1.1080	0.9835
P19784	CSNK2A2	41.2	Casein kinase II subunit alpha' OS	11	4	1.1070	0.9835
Q99436	PSMB7	29.9	Proteasome subunit beta type-7 OS	44	11	1.1070	0.9803
Q04637	EIF4G1	175.4	Eukaryotic translation initiation factor 4 gamma 1 OS	25	26	1.1060	0.9835
Q9NZT2	OGFR	73.3	Opioid growth factor receptor OS	5	2	1.1060	0.9835
P36551	CPOX	50.1	Oxygen-dependent coproporphyrinogen-III oxidase, mitochondrial OS	36	13	1.1060	0.9822
O75934	BCAS2	26.1	Pre-mRNA-splicing factor SPF27 OS	46	7	1.1060	0.9728
P61769	B2M	13.7	Beta-2-microglobulin OS	81	22	1.1050	0.9835
Q9Y337	KLK5	32	Kallikrein-5 OS	18	4	1.1050	0.9823
Q9BZQ8	FAM129A	103.1	Protein Niban 1 OS	18	10	1.1050	0.9657
Q09666	AHNAK	628.7	Neuroblast differentiation-associated protein AHNAK OS	41	116	1.1040	0.9835
P48444	ARCN1	57.2	Coatomer subunit delta OS	40	22	1.1030	0.9835
E5RFF9	GINS4	22.6	DNA replication complex GINS protein SLD5 OS	36	4	1.1030	0.9835
O00151	PDLIM1	36	PDZ and LIM domain protein 1 OS	95	28	1.1030	0.9835
P19883	FST	38	Follistatin OS	54	17	1.1020	0.9835
P07900	HSP90AA1	84.6	Heat shock protein HSP 90-alpha OS	79	105	1.1020	0.9835
Q15233	NONO	54.2	Non-POU domain-containing octamer-binding protein OS	50	19	1.1020	0.9835
P19338	NCL	76.6	Nucleolin OS	49	49	1.1020	0.9835

Q13332	PTPRS	216.9	Receptor-type tyrosine-protein phosphatase S OS	3	3	1.1010	0.9835
Q9Y3F4	STRAP	38.4	Serine-threonine kinase receptor-associated protein OS	61	16	1.1010	0.9835
A0A0A0MSK5	TOR1AIP1	52.4	Torsin-1A-interacting protein 1 OS	11	3	1.1010	0.9835
P62750	RPL23A	17.7	60S ribosomal protein L23a OS	43	6	1.1000	0.9640
O00115	DNASE2	39.6	Deoxyribonuclease-2-alpha OS	29	9	1.1000	0.9853
P09668	CTSH	37.4	Pro-cathepsin H OS	35	9	1.1000	0.9835
O95831	AIFM1	66.9	Apoptosis-inducing factor 1, mitochondrial OS	33	16	1.0990	0.9835
Q8NEZ5	FBXO22	44.5	F-box only protein 22 OS	8	2	1.0990	0.9835
Q86U42	PABPN1	32.7	Polyadenylate-binding protein 2 OS	31	5	1.0990	0.9888
Q9UJA5	TRMT6	55.8	tRNA (adenine(58)-N(1))-methyltransferase non-catalytic subunit TRM6 OS	8	2	1.0990	0.9871
M0QY97	ZC3H4	95.5	Zinc finger CCCH domain-containing protein 4 (Fragment) OS	12	6	1.0990	0.9900
Q9P287	BCCIP	36	BRCA2 and CDKN1A-interacting protein OS	20	3	1.0980	0.9835
P61201	COPS2	51.6	COP9 signalosome complex subunit 2 OS	25	7	1.0980	0.9835
P21333	FLNA	280.6	Filamin-A OS	60	144	1.0980	0.9900
Q8IZ73	RPUSD2	61.3	RNA pseudouridylate synthase domain-containing protein 2 OS	20	7	1.0980	0.9835
J3KS05	CBX1	20	Chromobox protein homolog 1 (Fragment) OS	35	4	1.0970	0.9835
P28066	PSMA5	26.4	Proteasome subunit alpha type-5 OS	66	18	1.0970	0.9923
Q9NR45	NANS	40.3	Sialic acid synthase OS	55	18	1.0970	0.9918
P61978-3	HNRNPK	48.5	Isoform 3 of Heterogeneous nuclear ribonucleoprotein K OS	74	36	1.0960	0.9942
Q86V48	LUZP1	120.2	Leucine zipper protein 1 OS	3	2	1.0960	0.9888
Q15019	SEPT2	41.5	Septin-2 OS	77	28	1.0960	0.9942
P29144	TPP2	138.3	Tripeptidyl-peptidase 2 OS	14	13	1.0960	0.9918
Q14694	USP10	87.1	Ubiquitin carboxyl-terminal hydrolase 10 OS	13	5	1.0960	0.9900
Q01518	CAP1	51.9	Adenylyl cyclase-associated protein 1 OS	68	39	1.0950	0.9949
O75390	CS	51.7	Citrate synthase, mitochondrial OS	43	14	1.0950	0.9959
P52943	CRIP2	22.5	Cysteine-rich protein 2 OS	36	5	1.0950	0.9942
Q02539	HIST1H1A	21.8	Histone H1.1 OS	32	10	1.0950	0.9904
P78371	CCT2	57.5	T-complex protein 1 subunit beta OS	86	54	1.0950	0.9959
P50991	CCT4	57.9	T-complex protein 1 subunit delta OS	75	39	1.0940	0.9968
H0YKU1	TMOD3	20.9	Tropomodulin-3 (Fragment) OS	57	10	1.0940	0.9942
Q13867	BLMH	52.5	Bleomycin hydrolase OS	22	8	1.0930	0.9973
Q04941	PLP2	16.7	Proteolipid protein 2 OS	9	1	1.0930	0.9973
G3V2T6	N/A	22.6	Uncharacterized protein (Fragment) OS	5	1	1.0930	0.9973
Q92598	HSPH1	96.8	Heat shock protein 105 kDa OS	55	48	1.0920	0.9976
P09496-2	CLTA	23.6	Isoform Non-brain of Clathrin light chain A OS	29	7	1.0920	0.9973
P14678	SNRPB	24.6	Small nuclear ribonucleoprotein-associated proteins B and B' OS	26	7	1.0920	0.9973
E9PFG7	OGDH	99	2-oxoglutarate dehydrogenase, mitochondrial OS	2	1	1.0910	0.9904
A6NKB8	RNPEP	68.1	Aminopeptidase B OS	49	22	1.0910	0.9976
E9PJA2	CSF1	31.2	Macrophage colony-stimulating factor 1 (Fragment) OS	9	2	1.0910	0.9942
P78504	JAG1	133.7	Protein jagged-1 OS	14	10	1.0910	0.9835
P63010	AP2B1	104.5	AP-2 complex subunit beta OS	24	20	1.0900	0.9976
P39748	FEN1	42.6	Flap endonuclease 1 OS	19	5	1.0900	0.9971
P30044	PRDX5	22.1	Peroxiredoxin-5, mitochondrial OS	60	16	1.0900	0.9976
P26006	ITGA3	116.5	Integrin alpha-3 OS	3	3	1.0890	0.9918
Q9UDY2-3	TJP2	131.3	Isoform C1 of Tight junction protein ZO-2 OS	18	12	1.0890	0.9888

K7EKI8	PPL	204.4	Periplakin OS	23	25	1.0890	0.9976
P61604	HSPE1	10.9	10 kDa heat shock protein, mitochondrial OS	100	21	1.0880	0.9976
Q8WU90	ZC3H15	48.6	Zinc finger CCCH domain-containing protein 15 OS	2	1	1.0880	0.9962
Q13347	EIF3I	36.5	Eukaryotic translation initiation factor 3 subunit I OS	63	17	1.0870	0.9980
Q5D862	FLG2	247.9	Filaggrin-2 OS	0	1	1.0870	0.9942
H0Y4R1	IMPDH2	51	Inosine-5'-monophosphate dehydrogenase 2 (Fragment) OS	16	6	1.0870	0.9968
P62304	SNRPE	10.8	Small nuclear ribonucleoprotein E OS	29	2	1.0870	0.9976
O14579	COPE	34.5	Coatomer subunit epsilon OS	68	13	1.0850	0.9992
P11766	ADH5	39.7	Alcohol dehydrogenase class-3 OS	43	14	1.0840	0.9999
Q9NUQ9	FAM49B	36.7	CYFIP-related Rac1 interactor B OS	29	6	1.0840	0.9992
Q9UNN8	PROCR	26.7	Endothelial protein C receptor OS	26	5	1.0840	0.9986
O94985-2	CLSTN1	108.6	Isoform 2 of Calsyntenin-1 OS	39	27	1.0840	0.9999
P40121	CAPG	38.5	Macrophage-capping protein OS	70	17	1.0840	0.9999
P40926	MDH2	35.5	Malate dehydrogenase, mitochondrial OS	77	34	1.0840	0.9999
P61981	YWHAG	28.3	14-3-3 protein gamma OS	97	35	1.0830	0.9999
P31937	HIBADH	35.3	3-hydroxyisobutyrate dehydrogenase, mitochondrial OS	5	1	1.0830	0.9976
Q9Y3U8	RPL36	12.2	60S ribosomal protein L36 OS	33	3	1.0830	0.9942
Q05048	CSTF1	48.3	Cleavage stimulation factor subunit 1 OS	28	8	1.0830	0.9853
P19474	TRIM21	54.1	E3 ubiquitin-protein ligase TRIM21 OS	7	2	1.0830	0.9973
Q8NBS9	TXNDC5	47.6	Thioredoxin domain-containing protein 5 OS	38	15	1.0830	0.9992
A0A669KB35	MAPK8IP3	148.4	C-Jun-amino-terminal kinase-interacting protein 3 OS	2	3	1.0820	0.9976
Q8WXX5	DNAJC9	29.9	DnaJ homolog subfamily C member 9 OS	56	12	1.0820	0.9991
O60313	OPA1	111.6	Dynamin-like 120 kDa protein, mitochondrial OS	4	2	1.0820	0.9980
A0A140T9H5	HLA-C	41.3	HLA class I histocompatibility antigen, C alpha chain OS	25	7	1.0820	0.9973
H0YMJ0	MORF4L1	28.2	Mortality factor 4-like protein 1 (Fragment) OS	14	2	1.0820	0.9942
Q9Y265	RUVBL1	50.2	RuvB-like 1 OS	49	16	1.0820	0.9987
P30153	PPP2R1A	65.3	Serine/threonine-protein phosphatase 2A 65 kDa regulatory subunit A alpha isoform OS	58	27	1.0820	0.9985
P40227	CCT6A	58	T-complex protein 1 subunit zeta OS	61	34	1.0820	0.9987
Q14444	CAPRIN1	78.3	Caprin-1 OS	24	13	1.0810	0.9986
Q969H8	MYDGF	18.8	Myeloid-derived growth factor OS	26	5	1.0810	0.9998
Q9H2H8	PPIL3	18.1	Peptidyl-prolyl cis-trans isomerase-like 3 OS	35	4	1.0810	0.9976
Q9UMS4	PRPF19	55.1	Pre-mRNA-processing factor 19 OS	67	26	1.0810	0.9984
Q9Y570	PPME1	42.3	Protein phosphatase methylesterase 1 OS	41	12	1.0810	0.9990
Q02487	DSC2	99.9	Desmocollin-2 OS	9	7	1.0800	0.9986
P28482	MAPK1	41.4	Mitogen-activated protein kinase 1 OS	55	12	1.0800	0.9976
Q9UMX0	UBQLN1	62.5	Ubiquilin-1 OS	3	1	1.0800	0.9973
Q96CT7	CCDC124	25.8	Coiled-coil domain-containing protein 124 OS	34	6	1.0790	0.9976
O43776	NARS	62.9	Asparagine--tRNA ligase, cytoplasmic OS	39	25	1.0780	0.9976
Q13126	MTAP	31.2	S-methyl-5'-thioadenosine phosphorylase OS	53	14	1.0780	0.9976
P37837	TALDO1	37.5	Transaldolase OS	58	26	1.0780	0.9976
O14773	TPP1	61.2	Tripeptidyl-peptidase 1 OS	41	14	1.0780	0.9976
P07814	EPRS	170.5	Bifunctional glutamate/proline--tRNA ligase OS	34	38	1.0770	0.9976
P09429	HMGB1	24.9	High mobility group protein B1 OS	65	27	1.0770	0.9976
E7EQB2	LTF	76.6	Lactotransferrin (Fragment) OS	13	7	1.0770	0.9976
P23381	WARS	53.1	Tryptophan--tRNA ligase, cytoplasmic OS	70	29	1.0770	0.9976

Q01970	PLCB3	138.7	1-phosphatidylinositol 4,5-bisphosphate phosphodiesterase beta-3 OS	17	13	1.0760	0.9956
P10768	ESD	31.4	S-formylglutathione hydrolase OS	83	17	1.0760	0.9976
P18754	RCC1	44.9	Regulator of chromosome condensation OS	62	14	1.0750	0.9976
F5H5D3	TUBA1C	57.7	Tubulin alpha chain OS	56	39	1.0750	0.9974
A6NFX8	NUDT5	25.9	ADP-sugar pyrophosphatase OS	42	13	1.0740	0.9968
Q92522	H1FX	22.5	Histone H1.10 OS	15	2	1.0740	0.9976
P16035	TIMP2	24.4	Metalloproteinase inhibitor 2 OS	21	3	1.0740	0.9986
P11233	RALA	23.6	Ras-related protein Ral-A OS	14	4	1.0740	0.9999
P27348	YWHAQ	27.7	14-3-3 protein theta OS	80	20	1.0730	0.9953
P52799	EFNB2	36.9	Ephrin-B2 OS	11	2	1.0730	0.9976
Q01469	FABP5	15.2	Fatty acid-binding protein 5 OS	91	15	1.0730	0.9965
O95633	FSTL3	27.6	Follistatin-related protein 3 OS	22	3	1.0730	0.9976
Q5MIZ7	SMEK2	97.4	Serine/threonine-protein phosphatase 4 regulatory subunit 3B OS	1	1	1.0730	0.9976
Q9NP84	TNFRSF12A	13.9	Tumor necrosis factor receptor superfamily member 12A OS	38	4	1.0730	0.9953
O95336	PGLS	27.5	6-phosphogluconolactonase OS	97	22	1.0720	0.9942
P46108	CRK	33.8	Adapter molecule crk OS	33	7	1.0720	0.9999
Q16555	DPYSL2	62.3	Dihydropyrimidinase-related protein 2 OS	27	10	1.0720	0.9976
P17900	GM2A	20.8	Ganglioside GM2 activator OS	43	7	1.0720	0.9976
Q9H074	PAIP1	53.5	Polyadenylate-binding protein-interacting protein 1 OS	10	3	1.0720	0.9976
P52888	THOP1	78.8	Thimet oligopeptidase OS	36	21	1.0720	0.9942
Q93063	EXT2	82.2	Exostosin-2 OS	2	1	1.0710	0.9980
P25786	PSMA1	29.5	Proteasome subunit alpha type-1 OS	82	31	1.0710	0.9916
P15311	EZR	69.4	Ezrin OS	76	65	1.0700	0.9900
P19823	ITIH2	106.4	Inter-alpha-trypsin inhibitor heavy chain H2 OS	10	9	1.0700	0.9904
P61289	PSME3	29.5	Proteasome activator complex subunit 3 OS	50	12	1.0700	0.9900
Q15257	PPP2R4	40.6	Serine/threonine-protein phosphatase 2A activator OS	43	10	1.0700	0.9976
P23246	SFPQ	76.1	Splicing factor, proline- and glutamine-rich OS	37	22	1.0700	0.9897
P09012	SNRPA	31.3	U1 small nuclear ribonucleoprotein A OS	25	5	1.0700	0.9999
H3BNC9	N/A	64.5	40S ribosomal protein S17 OS	14	7	1.0690	0.9976
Q86UE4	MTDH	63.8	Protein LYRIC OS	17	6	1.0680	0.9976
Q9BRA2	TXNDC17	13.9	Thioredoxin domain-containing protein 17 OS	100	15	1.0680	0.9853
E9PNS3	TOLLIP	8.7	Toll-interacting protein OS	18	1	1.0680	0.9976
Q14847	LASP1	29.7	LIM and SH3 domain protein 1 OS	64	25	1.0670	0.9835
O60568	PLOD3	84.7	Multifunctional procollagen lysine hydroxylase and glycosyltransferase LH3 OS	43	24	1.0670	0.9835
O14744	PRMT5	72.6	Protein arginine N-methyltransferase 5 OS	23	9	1.0670	0.9993
Q12765	SCRN1	46.4	Secernin-1 OS	21	8	1.0670	0.9976
Q96FX7	TRMT61A	31.4	tRNA (adenine(58)-N(1))-methyltransferase catalytic subunit TRMT61A OS	25	4	1.0670	0.9991
H7C3A1	SRRT	56.7	Arsenite-resistance protein 2 (Fragment) OS	6	3	1.0660	0.9999
P24666	ACP1	18	Low molecular weight phosphotyrosine protein phosphatase OS	51	4	1.0660	0.9984
A0A590UJS9	DLG1	93.5	Disks large homolog 1 OS	8	5	1.0650	0.9976
P06744	GPI	63.1	Glucose-6-phosphate isomerase OS	77	47	1.0650	0.9835
P13489	RNH1	49.9	Ribonuclease inhibitor OS	72	24	1.0650	0.9835
Q9UJ70	NAGK	37.4	N-acetyl-D-glucosamine kinase OS	20	4	1.0640	0.9999
Q7RTV0	PHF5A	12.4	PHD finger-like domain-containing protein 5A OS	47	5	1.0640	0.9999
E9PK08	PPP6R3	16.3	Serine/threonine-protein phosphatase 6 regulatory subunit 3 (Fragment) OS	11	2	1.0640	0.9976

H0YAP1	ZNF706	5.8	Zinc finger protein 706 (Fragment) OS	22	1	1.0640	0.9968
Q9Y696	CLIC4	28.8	Chloride intracellular channel protein 4 OS	62	13	1.0630	0.9822
O43809	NUDT21	26.2	Cleavage and polyadenylation specificity factor subunit 5 OS	46	9	1.0630	0.9835
P62495	ETF1	49	Eukaryotic peptide chain release factor subunit 1 OS	18	7	1.0630	0.9882
P08582	MFI2	80.2	Melanotransferrin OS	7	4	1.0630	0.9991
B4DLR8	NQO1	22.8	NAD(P)H dehydrogenase [quinone] 1 OS	48	13	1.0630	0.9835
Q16822	PCK2	70.7	Phosphoenolpyruvate carboxykinase [GTP], mitochondrial OS	12	5	1.0630	0.9999
A0A0A0 MSS8	AKR1C3	36.8	Aldo-keto reductase family 1 member C3 OS	65	23	1.0620	0.9798
P00505	GOT2	47.5	Aspartate aminotransferase, mitochondrial OS	61	27	1.0620	0.9799
P55884	EIF3B	92.4	Eukaryotic translation initiation factor 3 subunit B OS	30	21	1.0620	0.9798
P14618-2	PKM	58	Isoform M1 of Pyruvate kinase PKM OS	96	88	1.0620	0.9847
P12004	PCNA	28.8	Proliferating cell nuclear antigen OS	87	27	1.0620	0.9817
Q9UNH7	SNX6	46.6	Sorting nexin-6 OS	12	3	1.0620	0.9980
P60174	TPI1	26.7	Triosephosphate isomerase OS	96	49	1.0620	0.9805
P31946	YWHAB	28.1	14-3-3 protein beta/alpha OS	85	30	1.0610	0.9835
P20827	EFNA1	23.8	Ephrin-A1 OS	13	4	1.0610	0.9974
Q13526	PIN1	18.2	Peptidyl-prolyl cis-trans isomerase NIMA-interacting 1 OS	58	8	1.0610	0.9835
P05121	SERPINE1	45	Plasminogen activator inhibitor 1 OS	33	10	1.0610	0.9835
P28072	PSMB6	25.3	Proteasome subunit beta type-6 OS	51	11	1.0610	0.9780
Q8WVM8	SCFD1	72.3	Sec1 family domain-containing protein 1 OS	16	7	1.0610	0.9976
P52788	SMS	41.2	Spermine synthase OS	72	16	1.0610	0.9835
P50990	CCT8	59.6	T-complex protein 1 subunit theta OS	70	40	1.0610	0.9774
P12956	XRCC6	69.8	X-ray repair cross-complementing protein 6 OS	47	33	1.0610	0.9798
H3BNX8	COX5A	17.2	Cytochrome c oxidase polypeptide Va OS	27	3	1.0600	0.9980
Q99613	EIF3C	105.3	Eukaryotic translation initiation factor 3 subunit C OS	29	18	1.0600	0.9765
A0A2R8Y 7R2	HBB	12.2	Hemoglobin subunit beta OS	25	3	1.0600	0.9973
Q9NSE4	IARS2	113.7	Isoleucine--tRNA ligase, mitochondrial OS	6	4	1.0600	0.9992
J3KTL2	SRSF1	28.3	Serine/arginine-rich splicing factor 1 OS	57	14	1.0600	0.9765
Q13177	PAK2	58	Serine/threonine-protein kinase PAK 2 OS	32	10	1.0600	0.9835
O60493	SNX3	18.8	Sorting nexin-3 OS	30	4	1.0600	0.9999
O75821	EIF3G	35.6	Eukaryotic translation initiation factor 3 subunit G OS	31	4	1.0590	0.9980
Q9NQ88	TIGAR	30	Fructose-2,6-bisphosphatase TIGAR OS	33	7	1.0590	0.9980
P00966	ASS1	46.5	Argininosuccinate synthase OS	76	28	1.0580	0.9739
Q9UHB6-4	LIMA1	85.2	Isoform 4 of LIM domain and actin-binding protein 1 OS	37	16	1.0580	0.9735
D6RIH3	TCEAL4	8.6	Transcription elongation factor A protein-like 4 (Fragment) OS	9	1	1.0580	0.9992
Q9UK76	JPT1	16	Jupiter microtubule associated homolog 1 OS	52	8	1.0570	0.9728
O43447	PPIH	19.2	Peptidyl-prolyl cis-trans isomerase H OS	75	9	1.0570	0.9918
P14618	PKM	57.9	Pyruvate kinase PKM OS	96	92	1.0570	0.9722
Q13596	SNX1	59	Sorting nexin-1 OS	14	5	1.0570	0.9986
A0A1B0G TJ7	ADSL	54.4	Adenylosuccinate lyase OS	40	12	1.0560	0.9722
Q6FI81	CIAPIN1	33.6	Anamorsin OS	42	8	1.0560	0.9751
Q5JTJ3	COA6	14.1	Cytochrome c oxidase assembly factor 6 homolog OS	22	2	1.0560	0.9976
A0A3B3I UC0	ITM2B	24.2	Integral membrane protein 2 OS	15	3	1.0560	0.9942
Q9H910	JPT2	20.1	Jupiter microtubule associated homolog 2 OS	92	15	1.0560	0.9709
Q16513	PKN2	112	Serine/threonine-protein kinase N2 OS	1	1	1.0560	0.9976

O43765	SGTA	34	Small glutamine-rich tetratricopeptide repeat-containing protein alpha OS	30	10	1.0560	0.9796
Q9NVG8	TBC1D13	46.5	TBC1 domain family member 13 OS	3	1	1.0560	0.9980
P48643	CCT5	59.6	T-complex protein 1 subunit epsilon OS	89	40	1.0560	0.9690
P53396	ACLY	120.8	ATP-citrate synthase OS	49	45	1.0550	0.9679
Q8IZP0	ABI1	55	Abl interactor 1 OS	13	4	1.0540	0.9976
P13164	IFITM1	14	Interferon-induced transmembrane protein 1 OS	17	1	1.0540	0.9976
Q9BZZ5-2	API5	56.7	Isoform 2 of Apoptosis inhibitor 5 OS	26	10	1.0540	0.9645
F8W809	TXNRD1	54.6	Thioredoxin-disulfide reductase OS	59	20	1.0540	0.9640
A0A0C4DGN6	GIT1	83.1	ARF GTPase-activating protein GIT1 OS	2	2	1.0530	0.9976
Q96CN7	ISOC1	32.2	Isochorismatase domain-containing protein 1 OS	22	5	1.0530	0.9966
Q8NC51	SERBP1	44.9	Plasminogen activator inhibitor 1 RNA-binding protein OS	39	22	1.0520	0.9593
P36873	PPP1CC	37	Serine/threonine-protein phosphatase PP1-gamma catalytic subunit OS	25	9	1.0520	0.9976
H0YJA2	ZC3H14	73.3	Zinc finger CCCH domain-containing protein 14 (Fragment) OS	8	3	1.0520	0.9976
O00571	DDX3X	73.2	ATP-dependent RNA helicase DDX3X OS	4	2	1.0500	0.9976
Q9BXJ9	NAA15	101.2	N-alpha-acetyltransferase 15, NatA auxiliary subunit OS	20	11	1.0500	0.9801
G3V2I9	NDRG2	28.2	N-myc downstream-regulated gene 2 protein (Fragment) OS	9	1	1.0500	0.9984
P55072	VCP	89.3	Transitional endoplasmic reticulum ATPase OS	87	82	1.0500	0.9547
J3KT73	RPL38	7.6	60S ribosomal protein L38 OS	20	2	1.0490	0.9976
J3KP58	CLIP1	148	CAP-Gly domain-containing linker protein 1 OS	4	4	1.0490	0.9976
P60983	GMFB	16.7	Glia maturation factor beta OS	65	11	1.0490	0.9663
P09237	MMP7	29.7	Matrilysin OS	6	1	1.0490	0.9976
P43034	PAFAH1B1	46.6	Platelet-activating factor acetylhydrolase IB subunit alpha OS	49	18	1.0490	0.9536
Q8N398	VWA5B2	131.6	von Willebrand factor A domain-containing protein 5B2 OS	2	1	1.0490	0.9714
B4DEE8	MGMT	25	6-O-methylguanine-DNA methyltransferase OS	8	1	1.0480	0.9976
P05141	SLC25A5	32.8	ADP/ATP translocase 2 OS	8	1	1.0480	0.9971
P09497	CLTB	25.2	Clathrin light chain B OS	27	6	1.0480	0.9625
P84090	ERH	12.3	Enhancer of rudimentary homolog OS	63	9	1.0480	0.9536
E7EX17	EIF4B	69.7	Eukaryotic translation initiation factor 4B OS	39	19	1.0480	0.9536
P30043	BLVRB	22.1	Flavin reductase (NADPH) OS	22	4	1.0480	0.9871
F5H345	HMBS	35.7	Hydroxymethylbilane synthase OS	25	6	1.0480	0.9976
Q9Y520	PRRC2C	316.7	Protein PRRC2C OS	2	4	1.0480	0.9956
Q9NTI5	PDS5B	164.6	Sister chromatid cohesion protein PDS5 homolog B OS	0	1	1.0480	0.9976
P62269	RPS18	17.7	40S ribosomal protein S18 OS	30	3	1.0470	0.9976
P14550	AKR1A1	36.6	Aldo-keto reductase family 1 member A1 OS	84	19	1.0470	0.9525
Q03154	ACY1	45.9	Aminoacylase-1 OS	39	9	1.0470	0.9835
P09525	ANXA4	35.9	Annexin A4 OS	25	7	1.0470	0.9626
P29692	EEF1D	31.1	Elongation factor 1-delta OS	60	23	1.0470	0.9525
P00338	LDHA	36.7	L-lactate dehydrogenase A chain OS	95	54	1.0470	0.9530
Q8NBP7	PCSK9	74.2	Proprotein convertase subtilisin/kexin type 9 OS	28	13	1.0470	0.9525
A0A3B3IUA2	SNU13	30.1	Ribonucleoprotein OS	12	3	1.0470	0.9976
H9KV45	UBE2D3	15.9	Ubiquitin-conjugating enzyme E2 D3 OS	33	3	1.0470	0.9735
Q15437	SEC23B	86.4	Protein transport protein Sec23B OS	16	7	1.0460	0.9835
Q9Y230	RUVBL2	51.1	RuvB-like 2 OS	38	17	1.0460	0.9536
Q14126	DSG2	122.2	Desmoglein-2 OS	39	37	1.0450	0.9484
P05120	SERPINB2	46.6	Plasminogen activator inhibitor 2 OS	88	33	1.0450	0.9490

Q8WUF5	PPP1R13L	89	RelA-associated inhibitor OS	4	2	1.0450	0.9976
P59998	ARPC4	19.7	Actin-related protein 2/3 complex subunit 4 OS	51	9	1.0440	0.9484
A0A2U3TZL5	CD59	13.3	CD59 glycoprotein (Fragment) OS	33	4	1.0440	0.9837
P48506	GCLC	72.7	Glutamate--cysteine ligase catalytic subunit OS	39	19	1.0440	0.9484
P51970	NDUFA8	20.1	NADH dehydrogenase [ubiquinone] 1 alpha subcomplex subunit 8 OS	3	1	1.0440	0.9976
P04066	FUCA1	53.7	Tissue alpha-L-fucosidase OS	4	1	1.0440	0.9976
P07108	DBI	10	Acyl-CoA-binding protein OS	74	6	1.0430	0.9536
Q9UJU6	DBNL	48.2	Drebrin-like protein OS	45	17	1.0430	0.9477
P98179	RBM3	17.2	RNA-binding protein 3 OS	67	8	1.0430	0.9477
E7EUC7	UGP2	57.8	UTP--glucose-1-phosphate uridylyltransferase OS	40	17	1.0430	0.9525
P00390	GSR	56.2	Glutathione reductase, mitochondrial OS	28	13	1.0420	0.9463
Q10567-3	AP1B1	103.5	Isoform C of AP-1 complex subunit beta-1 OS	16	16	1.0420	0.9536
P35579	MYH9	226.4	Myosin-9 OS	55	114	1.0420	0.9463
Q15459	SF3A1	88.8	Splicing factor 3A subunit 1 OS	24	17	1.0420	0.9534
P36543	ATP6V1E1	26.1	V-type proton ATPase subunit E 1 OS	18	4	1.0420	0.9835
P12830	CDH1	97.4	Cadherin-1 OS	24	19	1.0400	0.9449
P60953	CDC42	21.2	Cell division control protein 42 homolog OS	41	7	1.0400	0.9835
Q9UMD9	COL17A1	150.3	Collagen alpha-1(XVII) chain OS	12	12	1.0400	0.9449
Q5TEC6	HIST2H3PS2	15.4	Histone H3 OS	48	13	1.0400	0.9536
H0YL72	IDH3A	35.8	Isocitrate dehydrogenase [NAD] subunit alpha, mitochondrial OS	9	3	1.0400	0.9942
Q58FG1	HSP90AA4P	47.7	Putative heat shock protein HSP 90-alpha A4 OS	12	7	1.0400	0.9953
Q13838	DDX39B	49	Spliceosome RNA helicase DDX39B OS	68	28	1.0400	0.9449
H0YKV4	FAM96A	14.9	Cytosolic iron-sulfur assembly component 2A OS	7	1	1.0380	0.9976
J3KTA4	DDX5	69	DEAD box protein 5 OS	9	4	1.0380	0.9976
Q16270	IGFBP7	29.1	Insulin-like growth factor-binding protein 7 OS	56	16	1.0380	0.9405
P14324-2	FDPS	40.5	Isoform 2 of Farnesyl pyrophosphate synthase OS	35	10	1.0380	0.9403
Q9BUL8	PDCD10	24.7	Programmed cell death protein 10 OS	33	5	1.0380	0.9536
P46777	RPL5	34.3	60S ribosomal protein L5 OS	46	12	1.0370	0.9372
O75131	CPNE3	60.1	Copine-3 OS	7	2	1.0370	0.9976
Q9H7D0	DOCK5	215.2	Dedicator of cytokinesis protein 5 OS	1	2	1.0370	0.9371
P42126	ECI1	32.8	Enoyl-CoA delta isomerase 1, mitochondrial OS	22	4	1.0370	0.9536
Q96G03	PGM2	68.2	Phosphoglucomutase-2 OS	36	15	1.0370	0.9375
Q9Y2B0	CNPY2	20.6	Protein canopy homolog 2 OS	59	8	1.0370	0.9494
Q9P2R3	ANKFY1	128.3	Rabankyrin-5 OS	3	2	1.0370	0.9971
O95861	BPNT1	33.4	3'(2'),5'-bisphosphate nucleotidase 1 OS	44	11	1.0360	0.9463
B4DPQ0	C1R	81.8	Complement subcomponent C1r OS	65	47	1.0360	0.9352
B4DY08	HNRNPC	32	Heterogeneous nuclear ribonucleoproteins C1/C2 OS	53	20	1.0360	0.9343
P07711	CTSL	37.5	Procathepsin L OS	20	8	1.0360	0.9355
Q9NUP9	LIN7C	21.8	Protein lin-7 homolog C OS	36	5	1.0360	0.9835
Q8NDN9	RCBTB1	58.2	RCC1 and BTB domain-containing protein 1 OS	2	1	1.0360	0.9817
B1AKD8	CROCC	149.1	Rootletin (Fragment) OS	2	2	1.0360	0.9359
Q9Y490	TLN1	269.6	Talin-1 OS	33	53	1.0360	0.9352
A0A494C0X0	TBCB	31.1	Tubulin-folding cofactor B OS	21	4	1.0360	0.9965
X6R390	AP2S1	18.4	AP complex subunit sigma OS	6	1	1.0350	0.9976
P09417	QDPR	25.8	Dihydropteridine reductase OS	34	6	1.0350	0.9765

Q15075	EEA1	162.4	Early endosome antigen 1 OS	11	11	1.0350	0.9403
Q92890-1	UFD1L	38.7	Isoform Long of Ubiquitin recognition factor in ER-associated degradation protein 1 OS	15	5	1.0350	0.9817
P31939	ATIC	64.6	Bifunctional purine biosynthesis protein ATIC OS	59	29	1.0340	0.9299
P09972	ALDOC	39.4	Fructose-bisphosphate aldolase C OS	43	20	1.0340	0.9299
Q6DKJ4	NXN	48.4	Nucleoredoxin OS	8	2	1.0340	0.9976
P53618	COPB1	107.1	Coatomer subunit beta OS	15	8	1.0330	0.9737
Q9UBT2	UBA2	71.2	SUMO-activating enzyme subunit 2 OS	39	14	1.0330	0.9371
P22102	GART	107.7	Trifunctional purine biosynthetic protein adenosine-3 OS	36	22	1.0330	0.9352
P61964	WDR5	36.6	WD repeat-containing protein 5 OS	28	7	1.0330	0.9470
P49792	RANBP2	358	E3 SUMO-protein ligase RanBP2 OS	3	7	1.0320	0.9900
P35573	AGL	174.7	Glycogen debranching enzyme OS	4	4	1.0320	0.9853
Q9UHY1	NRBP1	59.8	Nuclear receptor-binding protein OS	5	2	1.0320	0.9917
P63000	RAC1	21.4	Ras-related C3 botulinum toxin substrate 1 OS	35	7	1.0320	0.9403
Q7KZF4	SND1	101.9	Staphylococcal nuclease domain-containing protein 1 OS	51	39	1.0320	0.9273
Q03135	CAV1	20.5	Caveolin-1 OS	32	5	1.0310	0.9728
P11021	HSPA5	72.3	Endoplasmic reticulum chaperone BiP OS	64	54	1.0310	0.9240
Q8WUM4	PDCD6IP	96	Programmed cell death 6-interacting protein OS	32	21	1.0310	0.9299
Q5JR08	RHOC	21.5	Rho-related GTP-binding protein RhoC (Fragment) OS	14	2	1.0310	0.9973
E5RIA4	DNPEP hCG_15581	52	Aspartyl aminopeptidase OS	25	7	1.0300	0.9728
P24928	POLR2A	217	DNA-directed RNA polymerase II subunit RPB1 OS	0	1	1.0300	0.9835
Q9UHV9	PFDN2	16.6	Prefoldin subunit 2 OS	84	11	1.0300	0.9240
P63241	EIF5A	16.8	Eukaryotic translation initiation factor 5A-1 OS	84	26	1.0290	0.9228
P31151	S100A7	11.5	Protein S100-A7 OS	58	5	1.0290	0.9281
P52566	ARHGDIB	23	Rho GDP-dissociation inhibitor 2 OS	31	4	1.0290	0.9547
Q15847	ADIRF	7.9	Adipogenesis regulatory factor OS	84	6	1.0280	0.9228
P98172	EFNB1	38	Ephrin-B1 OS	27	6	1.0280	0.9403
Q14240	EIF4A2	46.4	Eukaryotic initiation factor 4A-II OS	35	14	1.0280	0.9835
P55209	NAP1L1	45.3	Nucleosome assembly protein 1-like 1 OS	70	21	1.0280	0.9228
Q10471	GALNT2	64.7	Polypeptide N-acetylgalactosaminyltransferase 2 OS	18	7	1.0280	0.9728
P62318	SNRPD3	13.9	Small nuclear ribonucleoprotein Sm D3 OS	44	7	1.0280	0.9228
H3BMD8	ARPP19	14.5	cAMP-regulated phosphoprotein 19 OS	21	3	1.0270	0.9463
P49441	INPP1	44	Inositol polyphosphate 1-phosphatase OS	23	7	1.0270	0.9750
O75083	WDR1	66.2	WD repeat-containing protein 1 OS	61	31	1.0270	0.9228
Q13619	CUL4A	87.6	Cullin-4A OS	8	4	1.0260	0.9888
P14625	HSP90B1	92.4	Endoplasmin OS	50	38	1.0260	0.9221
Q9UL25	RAB21	24.3	Ras-related protein Rab-21 OS	5	1	1.0250	0.9973
P43251	BTD	61.1	Biotinidase OS	2	1	1.0240	0.9970
Q86X76	NIT1	35.9	Deaminated glutathione amidase OS	27	6	1.0240	0.9567
A8MXV4	NUDT19	42.2	Nucleoside diphosphate-linked moiety X motif 19 OS	7	1	1.0240	0.9973
O15355	PPM1G	59.2	Protein phosphatase 1G OS	26	9	1.0240	0.9257
Q96EK4	THAP11	34.4	THAP domain-containing protein 11 OS	3	1	1.0240	0.9942
P07437	TUBB	49.6	Tubulin beta chain OS	76	37	1.0240	0.9191
P30084	ECHS1	31.4	Enoyl-CoA hydratase, mitochondrial OS	36	7	1.0230	0.9611
Q9P2E9	RRBP1	152.4	Ribosome-binding protein 1 OS	34	35	1.0230	0.9166
O15126	SCAMP1	37.9	Secretory carrier-associated membrane protein 1 OS	8	1	1.0230	0.9884

Q9UEU0	VTI1B	26.7	Vesicle transport through interaction with t-SNAREs homolog 1B OS	16	2	1.0230	0.9835
P49915	GMPS	76.7	GMP synthase [glutamine-hydrolyzing] OS	46	24	1.0210	0.9094
Q13409-2	DYNC1I2	70.6	Isoform 2B of Cytoplasmic dynein 1 intermediate chain 2 OS	16	6	1.0210	0.9228
H3BV22	PPP4C	23.1	Serine/threonine-protein phosphatase (Fragment) OS	19	4	1.0210	0.9798
P61221	ABCE1	67.3	ATP-binding cassette sub-family E member 1 OS	20	7	1.0200	0.9470
Q02750	MAP2K1	43.4	Dual specificity mitogen-activated protein kinase kinase 1 OS	20	6	1.0200	0.9640
P42704	LRPPRC	157.8	Leucine-rich PPR motif-containing protein, mitochondrial OS	1	1	1.0200	0.9463
P02545	LMNA	74.1	Prelamin-A/C OS	83	84	1.0200	0.9075
P19623	SRM	33.8	Spermidine synthase OS	11	4	1.0200	0.9645
J3KS31	ZNF207	35.1	BUB3-interacting and GLEBS motif-containing protein ZNF207 (Fragment) OS	4	2	1.0190	0.9749
P14174	MIF	12.5	Macrophage migration inhibitory factor OS	100	13	1.0190	0.9059
E9PNJ4	STIM1	57.6	Stromal interaction molecule 1 OS	4	1	1.0190	0.9942
A0A494C165	PEPD	57.7	Xaa-Pro dipeptidase (Fragment) OS	33	11	1.0190	0.9057
P35606	COPB2	102.4	Coatomer subunit beta' OS	19	12	1.0180	0.9299
P48637	GSS	52.4	Glutathione synthetase OS	74	33	1.0180	0.9045
Q86X29	LSR	71.4	Lipolysis-stimulated lipoprotein receptor OS	17	12	1.0180	0.9240
Q8N4Q1	CHCHD4	16	Mitochondrial intermembrane space import and assembly protein 40 OS	16	1	1.0180	0.9835
C9JUN5	CCDC12	10.5	Coiled-coil domain-containing protein 12 (Fragment) OS	33	2	1.0170	0.9536
Q9NQR4	NIT2	30.6	Omega-amidase NIT2 OS	84	17	1.0170	0.8996
Q9H444	CHMP4B	24.9	Charged multivesicular body protein 4b OS	32	7	1.0160	0.9536
Q8TAQ9	SUN3	40.5	SUN domain-containing protein 3 OS	2	2	1.0160	0.9498
H0YN65	CHST14	39.9	Carbohydrate sulfotransferase OS	3	1	1.0140	0.9904
G5E971	MMP13	55.8	Collagenase 3 OS	31	8	1.0140	0.9059
O75940	SMNDC1	26.7	Survival of motor neuron-related-splicing factor 30 OS	16	2	1.0140	0.9567
A0A087X2I1	PSMC6	45.8	26S proteasome regulatory subunit 10B OS	42	13	1.0130	0.9228
O60216	RAD21	71.6	Double-strand-break repair protein rad21 homolog OS	6	4	1.0130	0.9536
O60869	EDF1	16.4	Endothelial differentiation-related factor 1 OS	37	6	1.0130	0.8979
Q9C0B5	ZDHHC5	77.5	Palmitoyltransferase ZDHHC5 OS	2	1	1.0130	0.9766
Q9P0V9	SEPT10	52.6	Septin-10 OS	25	9	1.0130	0.9728
M0R3D6	RPL18A	16.7	60S ribosomal protein L18a (Fragment) OS	11	1	1.0120	0.9059
O00764	PDXK	35.1	Pyridoxal kinase OS	48	11	1.0120	0.9083
Q6NZY4	ZCCHC8	78.5	Zinc finger CCHC domain-containing protein 8 OS	3	1	1.0120	0.9835
O43324	EEF1E1	19.8	Eukaryotic translation elongation factor 1 epsilon-1 OS	14	2	1.0110	0.9835
P47756-2	CAPZB	30.6	Isoform 2 of F-actin-capping protein subunit beta OS	72	17	1.0110	0.8858
Q9BT73	PSMG3	13.1	Proteasome assembly chaperone 3 OS	12	1	1.0110	0.9855
D6REW4	LIN54	6.7	Protein lin-54 homolog OS	16	1	1.0110	0.9471
P50454	SERPINH1	46.4	Serpin H1 OS	51	19	1.0110	0.8866
P49458	SRP9	10.1	Signal recognition particle 9 kDa protein OS	51	7	1.0110	0.8858
Q15833	STXBP2	66.4	Syntaxin-binding protein 2 OS	18	6	1.0110	0.9765
E9PCW1	GOSR1	28.3	Golgi SNAP receptor complex member 1 OS	13	3	1.0100	0.9687
P31150	GDI1	50.6	Rab GDP dissociation inhibitor alpha OS	60	27	1.0100	0.8841
Q9ULV4	CORO1C	53.2	Coronin-1C OS	44	18	1.0090	0.8821
X6RLR1	DCTN3	19.9	Dynactin subunit 3 OS	25	3	1.0090	0.9860
P13639	EEF2	95.3	Elongation factor 2 OS	80	85	1.0090	0.8821
P32119	PRDX2	21.9	Peroxiredoxin-2 OS	85	21	1.0090	0.8821

A0A494C0G1	PGM3	55.4	Phosphoacetylglucosamine mutase OS	16	5	1.0090	0.9284
P38646	HSPA9	73.6	Stress-70 protein, mitochondrial OS	32	18	1.0090	0.8818
P06733	ENO1	47.1	Alpha-enolase OS	100	102	1.0080	0.8777
P11387	TOP1	90.7	DNA topoisomerase 1 OS	23	14	1.0080	0.8983
P07954	FH	54.6	Fumarate hydratase, mitochondrial OS	62	26	1.0080	0.8770
Q9H1E3	NUCKS1	27.3	Nuclear ubiquitous casein and cyclin-dependent kinase substrate 1 OS	52	9	1.0080	0.8766
P62937	PPIA	18	Peptidyl-prolyl cis-trans isomerase A OS	98	42	1.0080	0.8780
Q9Y446	PKP3	87	Plakophilin-3 OS	23	15	1.0080	0.9063
Q15819	UBE2V2	16.4	Ubiquitin-conjugating enzyme E2 variant 2 OS	54	8	1.0080	0.8780
Q9UBR2	CTSZ	33.8	Cathepsin Z OS	31	9	1.0070	0.8752
Q86VP6	CAND1	136.3	Cullin-associated NEDD8-dissociated protein 1 OS	34	31	1.0070	0.8766
Q15084	PDIA6	48.1	Protein disulfide-isomerase A6 OS	38	15	1.0070	0.8945
Q8WZ42	TTN	3813.7	Titin OS	0	4	1.0070	0.8914
Q14166	TTLL12	74.4	Tubulin--tyrosine ligase-like protein 12 OS	25	9	1.0070	0.9463
D3YTB1	RPL32	15.6	60S ribosomal protein L32 (Fragment) OS	23	2	1.0060	0.9548
B4DLN1	N/A	48.1	cDNA FLJ60124, highly similar to Mitochondrial dicarboxylate carrier OS	2	1	1.0050	0.8696
Q5T4U8	RABGGTB	17.3	Geranylgeranyl transferase type-2 subunit beta OS	30	3	1.0050	0.9730
P61758	VBP1	22.6	Prefoldin subunit 3 OS	55	9	1.0050	0.8702
F8W7C6	RPL10	18.6	60S ribosomal protein L10 OS	35	5	1.0040	0.9484
P60660-2	MYL6	17	Isoform Smooth muscle of Myosin light polypeptide 6 OS	86	16	1.0040	0.8653
Q9UGI8	TES	48	Testin OS	54	18	1.0030	0.8632
Q9Y678	COPG1	97.7	Coatomer subunit gamma-1 OS	23	13	1.0020	0.8980
Q9BV20	MRI1	39.1	Methylthioribose-1-phosphate isomerase OS	14	4	1.0010	0.9722
P60900	PSMA6	27.4	Proteasome subunit alpha type-6 OS	81	28	1.0010	0.8591
O75391	SPAG7	26	Sperm-associated antigen 7 OS	18	3	1.0010	0.9835
P62979	RPS27A	18	Ubiquitin-40S ribosomal protein S27a OS	60	10	1.0010	0.8593
P04004	VTN	54.3	Vitronectin OS	6	4	1.0010	0.8824
Q9NP97	DYNLRB1	10.9	Dynein light chain roadblock-type 1 OS	77	6	1.0000	0.8610
P30740	SERPINB1	42.7	Leukocyte elastase inhibitor OS	75	34	1.0000	0.8585
J3QR64	EIF4A1	23.8	RNA helicase (Fragment) OS	85	18	1.0000	0.9793
Q9UHD8	SEPT9	65.4	Septin-9 OS	39	15	1.0000	0.8585
P84077	ARF1	20.7	ADP-ribosylation factor 1 OS	60	7	0.9990	0.9825
P13645	KRT10	58.8	Keratin, type I cytoskeletal 10 OS	55	38	0.9990	0.8585
Q9Y5P6	GMPPB	39.8	Mannose-1-phosphate guanyltransferase beta OS	4	1	0.9990	0.9835
A0A0A0MSA9	PVR	42.9	Poliovirus receptor OS	16	6	0.9990	0.8824
P61586	RHOA	21.8	Transforming protein RhoA OS	30	5	0.9990	0.9221
P18077	RPL35A	12.5	60S ribosomal protein L35a OS	7	1	0.9980	0.9463
Q9NPD3	EXOSC4	26.4	Exosome complex component RRP41 OS	13	2	0.9980	0.9751
Q92692	NECTIN2	57.7	Nectin-2 OS	4	2	0.9980	0.9765
Q99536	VAT1	41.9	Synaptic vesicle membrane protein VAT-1 homolog OS	18	4	0.9980	0.9228
P61160	ACTR2	44.7	Actin-related protein 2 OS	55	17	0.9970	0.8549
Q99959	PKP2	97.4	Plakophilin-2 OS	4	3	0.9970	0.9648
H3BND8	USP7	105.5	Ubiquitin carboxyl-terminal hydrolase (Fragment) OS	8	6	0.9970	0.9449
P62241	RPS8	24.2	40S ribosomal protein S8 OS	52	11	0.9960	0.8536
B1AHB1	MCM5	77.5	DNA helicase OS	17	9	0.9960	0.9228

P40222	TXLNA	61.9	Alpha-taxilin OS	28	9	0.9950	0.8839
P16403	HIST1H1C	21.4	Histone H1.2 OS	40	15	0.9950	0.8766
H0YAR1	LOXL2	22.4	Lysyl oxidase homolog 2 (Fragment) OS	10	1	0.9950	0.9440
P61457	PCBD1	12	Pterin-4-alpha-carbinolamine dehydratase OS	51	5	0.9950	0.8842
P46939	UTRN	394.<br>2	Utrophin OS	0	2	0.9950	0.8581
Q8TEA8	DTD1	23.4	D-aminoacyl-tRNA deacylase 1 OS	15	2	0.9940	0.9228
A0A087X<br>2D0	SRSF3	10.3	Serine/arginine-rich-splicing factor 3 OS	37	3	0.9940	0.9494
A0A0C4D<br>GW6	C5orf51	17	UPF0600 protein C5orf51 OS	9	1	0.9940	0.9578
Q8NE71	ABCF1	95.9	ATP-binding cassette sub-family F member 1 OS	4	2	0.9930	0.9672
O60512	B4GALT3	43.9	Beta-1,4-galactosyltransferase 3 OS	2	1	0.9930	0.9739
F5GZ97	CCDC53	21	Coiled-coil domain-containing protein 53 OS	13	2	0.9920	0.9536
P24534	EEF1B2	24.7	Elongation factor 1-beta OS	88	14	0.9920	0.8398
P40925	MDH1	36.4	Malate dehydrogenase, cytoplasmic OS	57	26	0.9920	0.8411
P30530	AXL	98.3	Tyrosine-protein kinase receptor UFO OS	3	2	0.9920	0.9209
P21709	EPHA1	108.<br>1	Ephrin type-A receptor 1 OS	2	1	0.9910	0.9626
P00558	PGK1	44.6	Phosphoglycerate kinase 1 OS	99	68	0.9910	0.8363
P11717	IGF2R	274.<br>2	Cation-independent mannose-6-phosphate receptor OS	7	12	0.9900	0.8696
Q9H2U2	PPA2	37.9	Inorganic pyrophosphatase 2, mitochondrial OS	13	3	0.9900	0.9202
Q01650	SLC7A5	55	Large neutral amino acids transporter small subunit 1 OS	8	4	0.9900	0.8405
Q9BXT2	CACNG6	28.1	Voltage-dependent calcium channel gamma-6 subunit OS	3	1	0.9900	0.9728
Q8TF74	WIPF2	46.3	WAS/WASL-interacting protein family member 2 OS	11	4	0.9900	0.9083
O43681	ASNA1	38.8	ATPase GET3 OS	11	3	0.9890	0.9728
O60232	SSSCA1	21.5	Protein ZNRD2 OS	43	4	0.9890	0.9240
Q6NVY1	HIBCH	43.5	3-hydroxyisobutyryl-CoA hydrolase, mitochondrial OS	11	4	0.9880	0.9311
A0A3B3I<br>SH4	PPP1R12A	40.9	Protein phosphatase 1 regulatory subunit 12A (Fragment) OS	12	3	0.9880	0.9299
Q08211	DHX9	140.<br>9	ATP-dependent RNA helicase A OS	27	33	0.9870	0.8308
Q13630	TSTA3	35.9	GDP-L-fucose synthase OS	29	8	0.9870	0.8585
Q15149-4	PLEC	515.<br>9	Isoform 4 of Plectin OS	40	174	0.9870	0.8312
Q9GZT8	NIF3L1	41.9	NIF3-like protein 1 OS	25	7	0.9870	0.8585
A0A0A0<br>MQW3	SERPINB13	45.3	Serpin B13 OS	67	30	0.9870	0.8313
P00749	PLAU	48.5	Urokinase-type plasminogen activator OS	44	17	0.9870	0.8313
B5MDF5	RAN	26.2	GTP-binding nuclear protein Ran OS	49	19	0.9860	0.8272
O94776	MTA2	75	Metastasis-associated protein MTA2 OS	6	3	0.9860	0.9536
Q969S9	GFM2	86.5	Ribosome-releasing factor 2, mitochondrial OS	2	1	0.9860	0.9202
O60271	SPAG9	146.<br>1	C-Jun-amino-terminal kinase-interacting protein 4 OS	4	4	0.9850	0.9470
O43175	PHGDH	56.6	D-3-phosphoglycerate dehydrogenase OS	41	16	0.9850	0.8383
Q9NY33	DPP3	82.5	Dipeptidyl peptidase 3 OS	36	18	0.9850	0.8260
K7ELL7	PRKCSH	60.2	Glucosidase 2 subunit beta OS	37	19	0.9850	0.8260
P07737	PFN1	15	Profilin-1 OS	98	34	0.9850	0.8260
Q9UQ35	SRRM2	299.<br>4	Serine/arginine repetitive matrix protein 2 OS	2	3	0.9850	0.9679
Q15393	SF3B3	135.<br>5	Splicing factor 3B subunit 3 OS	30	24	0.9850	0.8265
O00622	CYR61	42	CCN family member 1 OS	22	6	0.9840	0.8344
Q16658	FSCN1	54.5	Fascin OS	81	49	0.9840	0.8244
P04075	ALDOA	39.4	Fructose-bisphosphate aldolase A OS	90	57	0.9840	0.8237
P08729	KRT7	51.4	Keratin, type II cytoskeletal 7 OS	40	18	0.9840	0.8506

Q9NZL9	MAT2B	37.5	Methionine adenosyltransferase 2 subunit beta OS	41	11	0.9840	0.8225
J3QQU6	SECTM1	15.9	Secreted and transmembrane protein 1 OS	23	2	0.9840	0.9274
P15924	DSP	331.6	Desmoplakin OS	11	22	0.9830	0.8344
P60228	EIF3E	52.2	Eukaryotic translation initiation factor 3 subunit E OS	41	16	0.9830	0.8470
P49903	SEPHS1	42.9	Selenide, water dikinase 1 OS	43	10	0.9830	0.8585
O75347	TBCA	12.8	Tubulin-specific chaperone A OS	64	10	0.9830	0.8187
P53999	SUB1	14.4	Activated RNA polymerase II transcriptional coactivator p15 OS	67	9	0.9820	0.8165
A0A0G2JJD3	ABHD16A	67.5	Alpha/beta hydrolase domain-containing protein 16A OS	1	1	0.9820	0.8932
A0A590UJ50	CUL1	87.6	Cullin-1 OS	13	6	0.9820	0.9449
Q14257	RCN2	36.9	Reticulocalbin-2 OS	23	4	0.9820	0.9335
Q9NW13	RBM28	85.7	RNA-binding protein 28 OS	2	2	0.9820	0.9418
Q8NBJ7	SUMF2	33.8	Inactive C-alpha-formylglycine-generating enzyme 2 OS	12	3	0.9810	0.9240
D6REK3	CWC27	43	Spliceosome-associated protein CWC27 homolog OS	13	2	0.9810	0.9494
P16949	STMN1	17.3	Stathmin OS	62	11	0.9810	0.8158
Q4KMP7	TBC1D10B	87.1	TBC1 domain family member 10B OS	4	2	0.9810	0.9477
H3BUD2	DCTN5	6	Dynactin subunit 5 OS	45	1	0.9800	0.9728
O60814	HIST1H2BK	13.9	Histone H2B type 1-K OS	83	23	0.9800	0.8124
Q9UQ80	PA2G4	43.8	Proliferation-associated protein 2G4 OS	72	30	0.9800	0.8138
P01034	CST3	15.8	Cystatin-C OS	67	15	0.9790	0.8095
O43278	SPINT1	58.4	Kunitz-type protease inhibitor 1 OS	43	23	0.9790	0.8095
Q01105	SET	33.5	Protein SET OS	55	30	0.9790	0.8103
Q08209	PPP3CA	58.7	Serine/threonine-protein phosphatase 2B catalytic subunit alpha isoform OS	6	3	0.9790	0.9240
P38606	ATP6V1A	68.3	V-type proton ATPase catalytic subunit A OS	39	19	0.9790	0.8095
E5RFH6	EPHX2	42.1	Bifunctional epoxide hydrolase 2 OS	2	1	0.9780	0.9536
P11586	MTHFD1	101.5	C-1-tetrahydrofolate synthase, cytoplasmic OS	34	27	0.9780	0.8087
B1AHL2	FBLN1	78.3	Fibulin-1 OS	28	12	0.9780	0.8058
Q9HAV7	GRPEL1	24.3	GrpE protein homolog 1, mitochondrial OS	18	3	0.9780	0.9222
P13928	ANXA8	36.9	Annexin A8 OS	16	4	0.9770	0.8914
P08603	CFH	139	Complement factor H OS	16	14	0.9770	0.8051
Q15293	RCN1	38.9	Reticulocalbin-1 OS	33	8	0.9770	0.8340
Q06124	PTPN11	68	Tyrosine-protein phosphatase non-receptor type 11 OS	9	3	0.9770	0.9484
H7BXE3	SLTM	48.8	SAFB-like transcription modulator (Fragment) OS	11	6	0.9760	0.9001
P17096	HMGA1	11.7	High mobility group protein HMG-I/HMG-Y OS	73	5	0.9750	0.7987
O94888	UBXN7	54.8	UBX domain-containing protein 7 OS	9	4	0.9750	0.9484
Q15785	TOMM34	34.5	Mitochondrial import receptor subunit TOM34 OS	4	1	0.9740	0.9696
P49721	PSMB2	22.8	Proteasome subunit beta type-2 OS	75	15	0.9740	0.7977
A2A2L5	RTFDC1	31.1	Replication termination factor 2 (Fragment) OS	13	2	0.9740	0.9547
E9PN89	HCFC1	34.8	Heat shock cognate 71 kDa protein (Fragment) OS	73	51	0.9730	0.7962
P51610	HCFC1	208.6	Host cell factor 1 OS	11	15	0.9730	0.8938
Q9Y6K5	OAS3	121.1	2'-5'-oligoadenylate synthase 3 OS	5	3	0.9720	0.9573
A6NDG6	PGP	34	Glycerol-3-phosphate phosphatase OS	23	5	0.9720	0.8657
Q16775	HAGH	33.8	Hydroxyacylglutathione hydrolase, mitochondrial OS	24	5	0.9720	0.8885
O60749	SNX2	58.4	Sorting nexin-2 OS	18	8	0.9720	0.8586
Q9BWD1	ACAT2	41.3	Acetyl-CoA acetyltransferase, cytosolic OS	72	15	0.9710	0.7978
P54819	AK2	26.5	Adenylate kinase 2, mitochondrial OS	90	26	0.9710	0.7868

A0A6I8P U73	DNAJC7	56.7	DnaJ homolog subfamily C member 7 OS	15	5	0.9710	0.8875
Q13564	NAE1	60.2	NEDD8-activating enzyme E1 regulatory subunit OS	14	4	0.9710	0.9477
M0R299	FBL	25.3	rRNA 2'-O-methyltransferase fibrillarin (Fragment) OS	11	2	0.9710	0.9536
Q15637	SF1	68.3	Splicing factor 1 OS	18	7	0.9710	0.8588
P68371	TUBB4B	49.8	Tubulin beta-4B chain OS	72	36	0.9710	0.7868
Q92945	KHSRP	73.1	Far upstream element-binding protein 2 OS	16	7	0.9700	0.8736
P14923	JUP	81.7	Junction plakoglobin OS	56	30	0.9700	0.7844
Q14697	GANAB	106. 8	Neutral alpha-glucosidase AB OS	39	28	0.9700	0.7851
Q99733	NAP1L4	42.8	Nucleosome assembly protein 1-like 4 OS	51	18	0.9700	0.7935
Q8TCT8	SPPL2A	58.1	Signal peptide peptidase-like 2A OS	3	1	0.9700	0.9209
P17980	PSMC3	49.2	26S proteasome regulatory subunit 6A OS	30	10	0.9690	0.8501
P18669	PGAM1	28.8	Phosphoglycerate mutase 1 OS	96	35	0.9690	0.7837
A0A2R8Y CH5	CTNNB1	85.2	Catenin beta-1 (Fragment) OS	29	16	0.9680	0.8128
P53621	COPA	138. 3	Coatomer subunit alpha OS	32	27	0.9680	0.7808
Q14566	MCM6	92.8	DNA replication licensing factor MCM6 OS	27	21	0.9680	0.7799
Q8TCS8	PNPT1	85.9	Polyribonucleotide nucleotidyltransferase 1, mitochondrial OS	2	1	0.9680	0.9632
Q99848	EBNA1BP2	34.8	Probable rRNA-processing protein EBP2 OS	5	1	0.9680	0.9687
A0A3B3I S67	TFPI2	27.7	Tissue factor pathway inhibitor OS	27	5	0.9680	0.8839
P08237	PFKM	85.1	ATP-dependent 6-phosphofructokinase, muscle type OS	7	4	0.9670	0.9299
P10909	CLU	52.5	Clusterin OS	32	15	0.9670	0.7798
P49589-3	CARS	94.6	Isoform 3 of Cysteine--tRNA ligase, cytoplasmic OS	37	23	0.9670	0.7798
P50452	SERPINB8	42.7	Serpin B8 OS	29	12	0.9670	0.8344
P05543	SERPINA7	46.3	Thyroxine-binding globulin OS	6	3	0.9670	0.8803
P33240	CSTF2	60.9	Cleavage stimulation factor subunit 2 OS	22	9	0.9660	0.8058
Q9UBQ5	EIF3K	25	Eukaryotic translation initiation factor 3 subunit K OS	39	6	0.9660	0.8124
Q6IA69	NADSYN1	79.2	Glutamine-dependent NAD(+) synthetase OS	1	1	0.9660	0.9631
Q9Y624	F11R	32.6	Junctional adhesion molecule A OS	39	7	0.9660	0.8151
P17174	GOT1	46.2	Aspartate aminotransferase, cytoplasmic OS	88	39	0.9650	0.7760
P62805	HIST1H4A	11.4	Histone H4 OS	68	13	0.9650	0.7787
P02787	TF	77	Serotransferrin OS	9	6	0.9650	0.8704
P22223	CDH3	91.4	Cadherin-3 OS	22	12	0.9640	0.8109
P11388	TOP2A	174. 3	DNA topoisomerase 2-alpha OS	3	2	0.9640	0.8169
P29508	SERPINB3	44.5	Serpin B3 OS	79	42	0.9640	0.7742
K7ERJ6	ZSWIM4	99.6	Zinc finger SWIM domain-containing protein 4 (Fragment) OS	1	1	0.9640	0.7735
Q9UHL4	DPP7	54.3	Dipeptidyl peptidase 2 OS	7	2	0.9630	0.9634
Q7Z422	SZRD1	17	SUZ domain-containing protein 1 OS	57	4	0.9630	0.8465
A0A0G2J QH2	RPS18	3.8	40S ribosomal protein S18 OS	39	1	0.9620	0.9498
P50238	CRIP1	8.5	Cysteine-rich protein 1 OS	88	6	0.9620	0.7713
C9JRZ2	GIGYF2	12.5	GRB10-interacting GYF protein 2 (Fragment) OS	30	2	0.9620	0.9536
P29218	IMPA1	30.2	Inositol monophosphatase 1 OS	47	10	0.9620	0.8051
P35527	KRT9	62	Keratin, type I cytoskeletal 9 OS	79	61	0.9620	0.7724
B7Z1I7	NRK	18.8	Nik-related protein kinase OS	4	1	0.9620	0.9228
Q00169	PITPNA	31.8	Phosphatidylinositol transfer protein alpha isoform OS	11	4	0.9620	0.8585
O14641	DVL2	78.9	Segment polarity protein dishevelled homolog DVL-2 OS	1	1	0.9620	0.9477
Q9Y5K5	UCHL5	37.6	Ubiquitin carboxyl-terminal hydrolase isozyme L5 OS	29	6	0.9610	0.9221

Q04323	UBXN1	33.3	UBX domain-containing protein 1 OS	22	3	0.9610	0.9405
P17655	CAPN2	79.9	Calpain-2 catalytic subunit OS	22	10	0.9600	0.8585
P04264	KRT1	66	Keratin, type II cytoskeletal 1 OS	74	71	0.9600	0.7648
Q14435	GALNT3	72.6	Polypeptide N-acetylgalactosaminyltransferase 3 OS	4	2	0.9600	0.9593
P14868	DARS	57.1	Aspartate--tRNA ligase, cytoplasmic OS	51	19	0.9590	0.7646
Q9BRX5	GINS3	24.5	DNA replication complex GINS protein PSF3 OS	22	3	0.9590	0.8981
Q13868	EXOSC2	32.8	Exosome complex component RRP4 OS	10	2	0.9590	0.9299
P12532	CKMT1B	47	Creatine kinase U-type, mitochondrial OS	71	32	0.9580	0.7565
P40189	IL6ST	103.<br>5	Interleukin-6 receptor subunit beta OS	2	1	0.9580	0.9477
Q9NQ48	LZTFL1	34.6	Leucine zipper transcription factor-like protein 1 OS	13	2	0.9580	0.8987
A0A087W<br>YT3	PTGES3	19.1	Prostaglandin E synthase 3 OS	32	7	0.9580	0.7798
A0A087X<br>0K9	TJP1	187.<br>7	Tight junction protein ZO-1 OS	6	6	0.9580	0.7903
P25787	PSMA2	25.9	Proteasome subunit alpha type-2 OS	82	19	0.9570	0.7556
P34897	SHMT2	56	Serine hydroxymethyltransferase, mitochondrial OS	33	12	0.9570	0.8058
Q7KZ85	SUPT6H	198.<br>9	Transcription elongation factor SPT6 OS	6	5	0.9570	0.9416
P30520	ADSS	50.1	Adenylosuccinate synthetase isozyme 2 OS	39	16	0.9560	0.7494
P51858	HDGF	26.8	Hepatoma-derived growth factor OS	70	20	0.9560	0.7510
P84243	H3F3A	15.3	Histone H3.3 OS	66	22	0.9560	0.9551
J3KNC0	GTF2A1	6.7	Transcription initiation factor IIA subunit 1 OS	25	1	0.9560	0.9525
P54577	YARS	59.1	Tyrosine--tRNA ligase, cytoplasmic OS	21	8	0.9560	0.8552
Q969E8	TSR2	20.9	Pre-rRNA-processing protein TSR2 homolog OS	12	3	0.9550	0.9365
Q15435	PPP1R7	41.5	Protein phosphatase 1 regulatory subunit 7 OS	17	5	0.9550	0.8585
K7EQJ5	RPS15	16.6	40S ribosomal protein S15 OS	29	2	0.9540	0.9536
P52209	PGD	53.1	6-phosphogluconate dehydrogenase, decarboxylating OS	66	33	0.9540	0.7490
P55145	MANF	20.7	Mesencephalic astrocyte-derived neurotrophic factor OS	41	4	0.9540	0.8051
Q9Y3I0	RTCB	55.2	RNA-splicing ligase RtcB homolog OS	47	20	0.9540	0.7494
P60866	RPS20	13.4	40S ribosomal protein S20 OS	42	4	0.9530	0.7450
Q99961	SH3GL1	41.5	Endophilin-A2 OS	26	5	0.9530	0.9228
P52926	HMGA2	11.8	High mobility group protein HMGI-C OS	39	2	0.9530	0.9299
P49773	HINT1	13.8	Histidine triad nucleotide-binding protein 1 OS	91	11	0.9530	0.7453
P06753-2	TPM3	29	Isoform 2 of Tropomyosin alpha-3 chain OS	90	38	0.9530	0.7446
P02750	LRG1	38.2	Leucine-rich alpha-2-glycoprotein OS	20	4	0.9530	0.8727
Q04837	SSBP1	17.2	Single-stranded DNA-binding protein, mitochondrial OS	70	9	0.9530	0.7464
A0A2R8Y<br>849	RPS24	15.2	40S ribosomal protein S24 OS	35	4	0.9520	0.9228
C9JD32	RPL23	9.7	60S ribosomal protein L23 (Fragment) OS	52	3	0.9520	0.9228
Q00839	HNRNPU	90.5	Heterogeneous nuclear ribonucleoprotein U OS	36	23	0.9520	0.7445
P05787	KRT8	53.7	Keratin, type II cytoskeletal 8 OS	65	30	0.9520	0.7445
O00214	LGALS8	35.8	Galectin-8 OS	6	2	0.9510	0.9449
O60925	PFDN1	14.2	Prefoldin subunit 1 OS	53	6	0.9510	0.7798
Q9Y5X1	SNX9	66.6	Sorting nexin-9 OS	5	2	0.9510	0.9299
P05783	KRT18	48	Keratin, type I cytoskeletal 18 OS	38	16	0.9500	0.7798
O00443	PIK3C2A	190.<br>6	Phosphatidylinositol 4-phosphate 3-kinase C2 domain-containing subunit alpha OS	1	1	0.9500	0.7481
P06703	S100A6	10.2	Protein S100-A6 OS	26	5	0.9500	0.8438
P62820	RAB1A	22.7	Ras-related protein Rab-1A OS	43	8	0.9500	0.9311
E9PPH5	ANP32E	13.1	Acidic leucine-rich nuclear phosphoprotein 32 family member E (Fragment) OS	18	2	0.9490	0.8766

Q04828	AKR1C1	36.8	Aldo-keto reductase family 1 member C1 OS	86	34	0.9490	0.7384
Q86YZ3	HRNR	282.2	Hornerin OS	12	5	0.9490	0.9299
P48594	SERPINB4	44.8	Serpin B4 OS	79	43	0.9490	0.7379
P54687	BCAT1	42.9	Branched-chain-amino-acid aminotransferase, cytosolic OS	4	1	0.9480	0.9525
Q9H0W9	C11orf54	35.1	Ester hydrolase C11orf54 OS	24	5	0.9480	0.9449
M0R043	GINS2	8.2	GINS complex subunit 2 (Fragment) OS	14	1	0.9480	0.9432
Q8WZ82	OVCA2	24.4	Esterase OVCA2 OS	4	1	0.9470	0.9240
A0A590UJG2	MACF1	614.5	Microtubule-actin cross-linking factor 1, isoforms 1/2/3/5 OS	1	5	0.9470	0.9228
O43684	BUB3	37.1	Mitotic checkpoint protein BUB3 OS	34	10	0.9470	0.7727
P04179	SOD2	24.7	Superoxide dismutase [Mn], mitochondrial OS	74	9	0.9470	0.7290
Q9Y262	EIF3L	66.7	Eukaryotic translation initiation factor 3 subunit L OS	42	18	0.9460	0.7245
Q4J6C6	PREPL	83.9	Prolyl endopeptidase-like OS	3	1	0.9460	0.9525
Q9Y5Y6	ST14	94.7	Suppressor of tumorigenicity 14 protein OS	15	7	0.9460	0.7795
Q8N3U4	STAG2	141.2	Cohesin subunit SA-2 OS	3	2	0.9450	0.9484
P12111	COL6A3	343.5	Collagen alpha-3(VI) chain OS	1	2	0.9450	0.9240
B0QYK0	EWSR1	64.9	RNA-binding protein EWS OS	8	3	0.9450	0.9240
Q6NY19	KANK3	88.4	KN motif and ankyrin repeat domain-containing protein 3 OS	1	1	0.9440	0.9536
Q14980	NUMA1	238.1	Nuclear mitotic apparatus protein 1 OS	31	49	0.9440	0.7213
P29401	TKT	67.8	Transketolase OS	90	80	0.9440	0.7196
B8ZZY2	AGFG1	56.4	Arf-GAP domain and FG repeat-containing protein 1 OS	11	3	0.9430	0.9228
O75976	CPD	152.8	Carboxypeptidase D OS	3	4	0.9430	0.9242
O75367	H2AFY	39.6	Core histone macro-H2A.1 OS	26	10	0.9430	0.7514
O60656	UGT1A9	59.9	UDP-glucuronosyltransferase 1A9 OS	1	1	0.9430	0.9174
P62877	RBX1	12.3	E3 ubiquitin-protein ligase RBX1 OS	16	1	0.9420	0.8974
P35244	RPA3	13.6	Replication protein A 14 kDa subunit OS	40	3	0.9420	0.8165
D6RER5	SEPTIN11	49.8	Septin-11 OS	36	12	0.9420	0.7494
Q14683	SMC1A	143.1	Structural maintenance of chromosomes protein 1A OS	8	7	0.9420	0.8585
Q13509	TUBB3	50.4	Tubulin beta-3 chain OS	57	25	0.9420	0.8020
P13798	APEH	81.2	Acylamino-acid-releasing enzyme OS	34	18	0.9410	0.7110
Q8N163	CCAR2	102.8	Cell cycle and apoptosis regulator protein 2 OS	20	11	0.9410	0.8185
Q07954	LRP1	504.3	Prolow-density lipoprotein receptor-related protein 1 OS	1	4	0.9410	0.7735
Q9Y3B8-3	REXO2	23.7	Isoform 3 of Oligoribonuclease, mitochondrial OS	42	10	0.9400	0.7290
P63313	TMSB10	5	Thymosin beta-10 OS	70	3	0.9400	0.7048
P04085	PDGFA	24	Platelet-derived growth factor subunit A OS	9	2	0.9390	0.9059
P46060	RANGAP1	63.5	Ran GTPase-activating protein 1 OS	48	24	0.9390	0.7021
E7ESD2	WASHC2A	140.1	WASH complex subunit 2A OS	5	4	0.9390	0.9352
P07384	CAPN1	81.8	Calpain-1 catalytic subunit OS	34	23	0.9380	0.6936
A0A0C4DFP4	NGLY1	72	N-glycanase 1 (Fragment) OS	3	2	0.9380	0.9477
Q7Z2Z2	EFTUD1	125.4	Elongation factor-like GTPase 1 OS	3	3	0.9370	0.9299
Q9UKD2	MRTO4	27.5	mRNA turnover protein 4 homolog OS	24	3	0.9370	0.9228
Q9Y6Q5	AP1M2	48.1	AP-1 complex subunit mu-2 OS	12	4	0.9360	0.8605
A0A087X0X3	HNRNPM	77.5	Heterogeneous nuclear ribonucleoprotein M OS	11	6	0.9360	0.8632
O43399	TPD52L2	22.2	Tumor protein D54 OS	72	11	0.9360	0.6848
P07858	CTSB	37.8	Cathepsin B OS	63	23	0.9350	0.6831
F8W651	COPZ1	13.4	Coatomer subunit zeta OS	51	5	0.9350	0.9153

Q6XQN6	NAPRT1	57.5	Nicotinate phosphoribosyltransferase OS	34	12	0.9350	0.7437
A8MWX3	WASH4P	51.6	Putative WAS protein family homolog 4 OS	3	1	0.9350	0.9484
P62191	PSMC1	49.2	26S proteasome regulatory subunit 4 OS	17	5	0.9340	0.8438
E9PNC7	DRAP1	16.6	Dr1-associated corepressor (Fragment) OS	21	2	0.9340	0.9299
O00154-4	ACOT7	37.4	Isoform 4 of Cytosolic acyl coenzyme A thioester hydrolase OS	41	12	0.9340	0.6793
Q9UNP9	PPIE	33.4	Peptidyl-prolyl cis-trans isomerase E OS	13	5	0.9340	0.7724
Q8WUW1	BRK1	8.7	Protein BRICK1 OS	47	4	0.9340	0.9004
Q92541	RTF1	80.3	RNA polymerase-associated protein RTF1 homolog OS	1	1	0.9340	0.9477
Q99714	HSD17B10	26.9	3-hydroxyacyl-CoA dehydrogenase type-2 OS	13	2	0.9330	0.9228
P07602	PSAP	58.1	Prosaposin OS	59	25	0.9330	0.6750
Q8NHU6	TDRD7	123.5	Tudor domain-containing protein 7 OS	1	1	0.9330	0.8344
O60462	NRP2	104.8	Neuropilin-2 OS	7	4	0.9320	0.9018
P30101	PDIA3	56.7	Protein disulfide-isomerase A3 OS	82	55	0.9320	0.6702
P46937	YAP1	54.4	Transcriptional coactivator YAP1 OS	24	6	0.9320	0.7494
H0YBI8	VEGFA	12.8	Vascular endothelial growth factor A (Fragment) OS	50	3	0.9320	0.8344
E7EU96	CSNK2A1	45.3	Casein kinase II subunit alpha OS	27	7	0.9310	0.8361
H0YDZ7	GDA	19	Guanine deaminase (Fragment) OS	10	1	0.9310	0.9477
P15170-3	GSPT1	68.7	Isoform 3 of Eukaryotic peptide chain release factor GTP-binding subunit ERF3A OS	20	8	0.9310	0.7679
A0A2R8Y5V9	TPM4	28.6	Tropomyosin alpha-4 chain OS	73	26	0.9310	0.6667
M0R210	RPS16	14.4	40S ribosomal protein S16 OS	40	6	0.9300	0.7798
P15104	GLUL	42	Glutamine synthetase OS	17	5	0.9300	0.8585
B4DJV5	PWP1	49	Periodic tryptophan protein 1 homolog OS	3	1	0.9300	0.9275
Q8NBJ5	COLGALT1	71.6	Procollagen galactosyltransferase 1 OS	4	2	0.9300	0.9299
P63220	RPS21	9.1	40S ribosomal protein S21 OS	93	13	0.9290	0.6555
P63261	ACTG1	41.8	Actin, cytoplasmic 2 OS	97	94	0.9290	0.6567
Q9NX46	ADPRHL2	38.9	ADP-ribose glycohydrolase ARH3 OS	29	8	0.9290	0.7942
P27797	CALR	48.1	Calreticulin OS	54	19	0.9290	0.6561
E9PIE3	CAVIN3	31.1	Caveolae-associated protein 3 OS	5	1	0.9290	0.9449
Q9BR76	CORO1B	54.2	Coronin-1B OS	37	13	0.9290	0.6566
Q15223	NECTIN1	57.1	Nectin-1 OS	13	6	0.9290	0.6555
P61163	ACTR1A	42.6	Alpha-centractin OS	61	13	0.9280	0.7245
A0A494C0R8	CLUH	150.4	Clustered mitochondria protein homolog OS	2	4	0.9280	0.8864
P78318	IGBP1	39.2	Immunoglobulin-binding protein 1 OS	22	4	0.9280	0.8823
Q9Y2E5	MAN2B2	113.9	Epididymis-specific alpha-mannosidase OS	2	2	0.9270	0.9236
P51532	SMARCA4	184.5	Transcription activator BRG1 OS	0	1	0.9270	0.8265
P10809	HSPD1	61	60 kDa heat shock protein, mitochondrial OS	82	63	0.9260	0.6502
O15305	PMM2	28.1	Phosphomannomutase 2 OS	39	6	0.9260	0.8528
P62140	PPP1CB	37.2	Serine/threonine-protein phosphatase PP1-beta catalytic subunit OS	30	10	0.9260	0.8128
Q9UNF0	PACSIN2	55.7	Protein kinase C and casein kinase substrate in neurons protein 2 OS	35	13	0.9250	0.6597
Q96D71	REPS1	86.6	RalBP1-associated Eps domain-containing protein 1 OS	1	1	0.9250	0.9299
O15400	STX7	29.8	Syntaxin-7 OS	34	7	0.9250	0.7936
Q5T6V5	C9orf64	39	Queuosine salvage protein OS	16	5	0.9240	0.8120
Q3LXA3	DAK; TKFC	58.9	Triokinase/FMN cyclase OS	16	8	0.9240	0.8053
Q99459	CDC5L	92.2	Cell division cycle 5-like protein OS	10	6	0.9230	0.7798
A0AVT1	UBA6	117.9	Ubiquitin-like modifier-activating enzyme 6 OS	4	4	0.9230	0.8945

O75531	BANF1	10.1	Barrier-to-autointegration factor OS	52	4	0.9220	0.8260
H0Y2Y8	ZYX	57.6	Zyxin (Fragment) OS	29	11	0.9220	0.7591
Q9BS26	ERP44	46.9	Endoplasmic reticulum resident protein 44 OS	40	15	0.9210	0.6332
A2A274	ACO2	87.8	Aconitate hydratase, mitochondrial OS	28	13	0.9200	0.7798
Q7L2H7	EIF3M	42.5	Eukaryotic translation initiation factor 3 subunit M OS	19	4	0.9200	0.9228
P17096-2	HMGA1	10.7	Isoform HMG-Y of High mobility group protein HMG-I/HMG-Y OS	70	5	0.9200	0.6294
P61970	NUTF2	14.5	Nuclear transport factor 2 OS	61	6	0.9190	0.6267
P62753	RPS6	28.7	40S ribosomal protein S6 OS	39	8	0.9180	0.6774
E9PB90	HK2	98.9	Hexokinase OS	4	4	0.9180	0.9403
A0A087W UE9	SYMPK	117. 4	Symplekin OS	1	1	0.9180	0.8721
P62701	RPS4X	29.6	40S ribosomal protein S4, X isoform OS	33	8	0.9170	0.7720
P02768	ALB	69.3	Albumin OS	26	22	0.9170	0.6128
Q15008	PSMD6	45.5	26S proteasome non-ATPase regulatory subunit 6 OS	19	5	0.9160	0.7724
A0A087W ZK9	EIF3H	39.6	Eukaryotic translation initiation factor 3 subunit H OS	45	9	0.9160	0.7528
P05204	HMGN2	9.4	Non-histone chromosomal protein HMG-17 OS	34	4	0.9160	0.6113
Q9UKX7	NUP50	50.1	Nuclear pore complex protein Nup50 OS	8	2	0.9160	0.8446
Q13243	SRSF5	31.2	Serine/arginine-rich splicing factor 5 OS	7	2	0.9160	0.9240
O75533	SF3B1	145. 7	Splicing factor 3B subunit 1 OS	13	10	0.9160	0.7888
E9PHK0	CLEC3B	17.8	Tetranectin OS	21	3	0.9150	0.8120
X6R700	CHTOP	23.6	Chromatin target of PRMT1 protein OS	6	1	0.9120	0.9228
P50583	NUDT2	16.8	Bis(5'-nucleosyl)-tetraphosphatase [asymmetrical] OS	24	3	0.9100	0.7729
Q9GZP4	PITHD1	24.2	PITH domain-containing protein 1 OS	33	5	0.9100	0.7724
Q9Y4Y9	LSM5	9.9	U6 snRNA-associated Sm-like protein LSm5 OS	27	1	0.9100	0.8344
Q86WR0	CCDC25	24.5	Coiled-coil domain-containing protein 25 OS	14	2	0.9080	0.8296
Q15170	TCEAL1	18.3	Transcription elongation factor A protein-like 1 OS	12	1	0.9080	0.9307
G8JLB3	PUS1	42.9	tRNA pseudouridine synthase A (Fragment) OS	7	2	0.9080	0.5915
Q9H8S9	MOB1A	25.1	MOB kinase activator 1A OS	7	2	0.9070	0.9228
P82094	TMF1	122. 8	TATA element modulatory factor OS	1	1	0.9070	0.9221
Q96CW1	AP2M1	49.6	AP-2 complex subunit mu OS	20	7	0.9060	0.7874
A0A0B4J 2A0	SHPK	51.5	FGGY_N domain-containing protein OS	10	3	0.9060	0.9171
Q9BWJ5	SF3B5	10.1	Splicing factor 3B subunit 5 OS	65	3	0.9060	0.7066
P09871	C1S	76.6	Complement C1s subcomponent OS	50	30	0.9050	0.5642
P00751	CFB	85.5	Complement factor B OS	41	24	0.9050	0.5660
O14879	IFIT3	56	Interferon-induced protein with tetratricopeptide repeats 3 OS	29	6	0.9050	0.7619
Q9GZL7	WDR12	47.7	Ribosome biogenesis protein WDR12 OS	13	3	0.9050	0.8581
J3KNF4	CCS	27.1	Copper chaperone for superoxide dismutase OS	11	2	0.9040	0.8501
Q7L576	CYFIP1	145. 1	Cytoplasmic FMR1-interacting protein 1 OS	4	5	0.9040	0.7437
H3BQZ7	HNRNPUL2-BSCL2 hCG_2044799	84.6	HCG2044799 OS	26	11	0.9040	0.6555
C9IZD3	ARHGAP17	24	Rho GTPase-activating protein 17 (Fragment) OS	4	1	0.9040	0.9228
P54725	RAD23A	39.6	UV excision repair protein RAD23 homolog A OS	12	2	0.9040	0.9123
P43652	AFM	69	Afamin OS	1	1	0.9030	0.7477
Q13162	PRDX4	30.5	Peroxiredoxin-4 OS	32	11	0.9030	0.7181
Q8IVF2	AHNAK2	616. 2	Protein AHNAK2 OS	4	4	0.9030	0.8260
P29590	PML	97.5	Protein PML OS	17	13	0.9030	0.5546
Q99470	SDF2	23	Stromal cell-derived factor 2 OS	6	1	0.9030	0.9043

Q96P16	RPRD1A	35.7	Regulation of nuclear pre-mRNA domain-containing protein 1A OS	2	1	0.9020	0.9259
P63165	SUMO1	11.6	Small ubiquitin-related modifier 1 OS	52	6	0.9020	0.5512
Q9Y2D5	AKAP2	94.6	A-kinase anchor protein 2 OS	4	2	0.9010	0.9018
Q14574	DSC3	99.9	Desmocollin-3 OS	27	26	0.9010	0.5458
P47897	QARS	87.7	Glutamine--tRNA ligase OS	35	16	0.9010	0.5677
P17301	ITGA2	129.2	Integrin alpha-2 OS	2	2	0.9010	0.9228
P33316-2	DUT	17.7	Isoform 2 of Deoxyuridine 5'-triphosphate nucleotidohydrolase, mitochondrial OS	87	16	0.9010	0.5457
P18583	SON	263.7	Protein SON OS	0	1	0.9010	0.5457
P21283	ATP6V1C1	43.9	V-type proton ATPase subunit C 1 OS	14	4	0.9010	0.8824
P24752	ACAT1	45.2	Acetyl-CoA acetyltransferase, mitochondrial OS	24	7	0.9000	0.9059
Q96DX5	ASB9	31.8	Ankyrin repeat and SOCS box protein 9 OS	13	2	0.9000	0.8963
Q14764	MVP	99.3	Major vault protein OS	35	19	0.9000	0.5578
Q8WVC0	LEO1	75.4	RNA polymerase-associated protein LEO1 OS	4	1	0.9000	0.9239
M0R050	EXOSC5	21.2	Exosome complex component RRP46 OS	6	1	0.8990	0.9238
O15371	EIF3D	63.9	Eukaryotic translation initiation factor 3 subunit D OS	39	13	0.8980	0.5417
Q5T1M5	FKBP15	133.5	FK506-binding protein 15 OS	1	1	0.8980	0.8474
Q96LW7	CARD19	25.6	Caspase recruitment domain-containing protein 19 OS	14	2	0.8970	0.8987
P02792	FTL	20	Ferritin light chain OS	51	7	0.8970	0.7557
Q7Z434	MAVS	56.5	Mitochondrial antiviral-signaling protein OS	16	4	0.8970	0.7977
H0YLH9	IL16	64.6	Pro-interleukin-16 (Fragment) OS	5	2	0.8970	0.5642
P10586	PTPRF	212.7	Receptor-type tyrosine-protein phosphatase F OS	5	7	0.8970	0.7648
A0A3B3IT92	MCM4	100.7	DNA helicase OS	22	13	0.8960	0.6539
Q9BQG0	MYBBP1A	148.8	Myb-binding protein 1A OS	1	1	0.8960	0.9107
P21281	ATP6V1B2	56.5	V-type proton ATPase subunit B, brain isoform OS	36	13	0.8960	0.5414
Q14204	DYNC1H1	532.1	Cytoplasmic dynein 1 heavy chain 1 OS	7	23	0.8950	0.6178
O43240	KLK10	30.2	Kallikrein-10 OS	32	10	0.8950	0.5227
Q32MZ4	LRRFIP1	89.2	Leucine-rich repeat flightless-interacting protein 1 OS	18	10	0.8950	0.6952
P83731	RPL24	17.8	60S ribosomal protein L24 OS	30	5	0.8940	0.6865
O15446	CD3EAP	55	DNA-directed RNA polymerase I subunit RPA34 OS	7	2	0.8940	0.7407
E7EUH7	PUS7	50.5	Pseudouridylate synthase 7 homolog OS	3	1	0.8930	0.8909
E5RGS9	CHRAC1	6.3	Chromatin accessibility complex protein 1 OS	73	3	0.8920	0.8006
P09382	LGALS1	14.7	Galectin-1 OS	56	7	0.8920	0.5897
H7C2W9	RPL31	12.8	60S ribosomal protein L31 (Fragment) OS	8	1	0.8910	0.8398
Q6P9B6	KIAA1609	51	MTOR-associated protein MEAK7 OS	16	4	0.8910	0.8265
Q9NS23	RASSF1	39.2	Ras association domain-containing protein 1 OS	2	1	0.8910	0.5356
Q8TD19	NEK9	107.1	Serine/threonine-protein kinase Nek9 OS	1	1	0.8910	0.8924
Q13428	TCOF1	152	Treacle protein OS	20	18	0.8910	0.5108
E9PQD7	RPS2	25.2	40S ribosomal protein S2 OS	28	7	0.8900	0.5870
M0R0R2	RPS5	25.3	40S ribosomal protein S5 OS	31	6	0.8900	0.6681
H7C3V9	SCLY	26.2	Selenocysteine lyase (Fragment) OS	18	3	0.8900	0.8585
O75822	EIF3J	29	Eukaryotic translation initiation factor 3 subunit J OS	46	10	0.8890	0.4997
A0A0G2JNZ7	DDR1	85.4	Receptor protein-tyrosine kinase OS	7	4	0.8890	0.7431
P36578	RPL4	47.7	60S ribosomal protein L4 OS	27	9	0.8880	0.7107
Q15424	SAFB	102.6	Scaffold attachment factor B1 OS	21	15	0.8880	0.4985
O00515	LAD1	57.1	Ladinin-1 OS	35	16	0.8870	0.4928

Q9H299	SH3BGRL3	10.4	SH3 domain-binding glutamic acid-rich-like protein 3 OS	92	10	0.8870	0.4925
A0A494C1Q1	CCL5	17	C-C motif chemokine 5 OS	8	1	0.8860	0.8398
A0A087X0S5	COL6A1	108.3	Collagen alpha-1(VI) chain OS	2	1	0.8860	0.8914
Q5SSJ5	HP1BP3	61.2	Heterochromatin protein 1-binding protein 3 OS	7	2	0.8860	0.7555
A0A0C4DGG1	PACSIN3	46.5	Protein kinase C and casein kinase substrate in neurons protein 3 (Fragment) OS	15	3	0.8860	0.9161
Q96J01	THOC3	38.7	THO complex subunit 3 OS	10	3	0.8860	0.9076
Q00341	HDLBP	141.4	Vigilin OS	4	4	0.8860	0.8777
A0A0U1RQM3	MTHFS	26.1	5-formyltetrahydrofolate cyclo-ligase OS	8	2	0.8850	0.9191
Q6NZI2	CAVIN1	43.5	Caveolae-associated protein 1 OS	10	4	0.8850	0.7443
P32320	CDA	16.2	Cytidine deaminase OS	28	2	0.8850	0.5808
P36957	DLST	48.7	Dihydrolipoyllysine-residue succinyltransferase component of 2-oxoglutarate dehydrogenase complex, mitochondrial OS	4	2	0.8850	0.8605
Q8IY67	RAVER1	63.8	Ribonucleoprotein PTB-binding 1 OS	3	1	0.8850	0.9191
A0A087X260	TARDBP	34.2	TAR DNA-binding protein 43 OS	27	6	0.8850	0.5740
Q5T4S7	UBR4	573.5	E3 ubiquitin-protein ligase UBR4 OS	1	2	0.8840	0.8605
R4GMQ1	KDM1A	93.5	Lysine-specific histone demethylase OS	2	1	0.8840	0.8607
Q04446	GBE1	80.4	1,4-alpha-glucan-branching enzyme OS	12	6	0.8830	0.7431
Q68E01	INTS3	118	Integrator complex subunit 3 OS	1	1	0.8830	0.8165
Q9UMR2	DDX19B	53.9	ATP-dependent RNA helicase DDX19B OS	7	2	0.8820	0.8664
J3KR23	IST1	31.2	IST1 homolog (Fragment) OS	2	1	0.8820	0.8585
Q9NZ08	ERAP1	107.2	Endoplasmic reticulum aminopeptidase 1 OS	1	1	0.8810	0.8607
P68366	TUBA4A	49.9	Tubulin alpha-4A chain OS	56	31	0.8810	0.4711
Q9NQW7	XPNPEP1	69.9	Xaa-Pro aminopeptidase 1 OS	23	8	0.8810	0.8121
Q6P587	FAHD1	24.8	Acylpyruvase FAHD1, mitochondrial OS	30	5	0.8800	0.7527
Q8N6M0	OTUD6B	33.8	Deubiquitinase OTUD6B OS	6	1	0.8800	0.8987
Q3MHD2	LSM12	21.7	Protein LSM12 homolog OS	14	2	0.8800	0.8585
Q96K17	BTF3L4	17.3	Transcription factor BTF3 homolog 4 OS	45	4	0.8800	0.6848
Q14152	EIF3A	166.5	Eukaryotic translation initiation factor 3 subunit A OS	30	39	0.8790	0.4619
P34096	RNASE4	16.8	Ribonuclease 4 OS	16	2	0.8790	0.9018
E9PKZ0	RPL8	22.4	60S ribosomal protein L8 (Fragment) OS	39	6	0.8760	0.6340
P13500	CCL2	11	C-C motif chemokine 2 OS	46	4	0.8760	0.5443
P42285	SKIV2L2	117.7	Exosome RNA helicase MTR4 OS	10	9	0.8760	0.7080
U3KQ69	MTG1	32.6	Mitochondrial GTPase 1 OS	2	1	0.8760	0.8185
Q9Y2W1	THRAP3	108.6	Thyroid hormone receptor-associated protein 3 OS	14	8	0.8760	0.6294
P35998	PSMC2	48.6	26S proteasome regulatory subunit 7 OS	40	12	0.8750	0.6239
O75116	ROCK2	160.8	Rho-associated protein kinase 2 OS	1	1	0.8750	0.8545
A0A590UK80	N/A	121	Uncharacterized protein (Fragment) OS	2	2	0.8750	0.7224
P62424	RPL7A	30	60S ribosomal protein L7a OS	45	13	0.8730	0.5102
P13861	PRKAR2A	45.5	cAMP-dependent protein kinase type II-alpha regulatory subunit OS	13	4	0.8730	0.8383
Q5RKV6	EXOSC6	28.2	Exosome complex component MTR3 OS	7	2	0.8720	0.8795
Q12904	AIMP1	34.3	Aminoacyl tRNA synthase complex-interacting multifunctional protein 1 OS	44	10	0.8710	0.4271
Q9H3P7	ACBD3	60.6	Golgi resident protein GCP60 OS	16	6	0.8700	0.7798
Q9BUF5	TUBB6	49.8	Tubulin beta-6 chain OS	27	13	0.8690	0.7682
O43172	PRPF4	58.4	U4/U6 small nuclear ribonucleoprotein Prp4 OS	10	3	0.8690	0.8546
Q13541	EIF4EBP1	12.6	Eukaryotic translation initiation factor 4E-binding protein 1 OS	79	6	0.8680	0.4468

O75607	NPM3	19.3	Nucleoplasmin-3 OS	30	3	0.8680	0.7245
P48507	GCLM	30.7	Glutamate--cysteine ligase regulatory subunit OS	21	5	0.8670	0.5278
A2BFX2	HLA-DRB1	30.2	HLA class II histocompatibility antigen DR beta chain OS	9	1	0.8670	0.8438
Q9HCD5	NCOA5	65.5	Nuclear receptor coactivator 5 OS	9	3	0.8670	0.7851
E9PCM6	ARHGAP4	85.3	Rho GTPase-activating protein 4 OS	1	1	0.8670	0.8051
A0A3B3I TS5	N/A	64.5	DNA polymerase alpha subunit B OS	2	1	0.8660	0.8696
Q9BY32	ITPA	21.4	Inosine triphosphate pyrophosphatase OS	58	9	0.8660	0.6328
O60220	TIMM8A	11	Mitochondrial import inner membrane translocase subunit Tim8 A OS	65	5	0.8660	0.6277
Q14498	RBM39	59.3	RNA-binding protein 39 OS	11	3	0.8660	0.8450
Q9UII2	ATPIF1	12.2	ATPase inhibitor, mitochondrial OS	33	4	0.8650	0.6384
P03956	MMP1	54	Interstitial collagenase OS	43	19	0.8650	0.4027
Q5JWF2	GNAS	111	Guanine nucleotide-binding protein G(s) subunit alpha isoforms XLas OS	4	3	0.8640	0.7579
A0A2R8Y 793	ACTB	34.1	Actin, cytoplasmic 1 (Fragment) OS	99	77	0.8630	0.3957
Q16719	KYNU	52.3	Kynureninase OS	19	5	0.8630	0.7956
Q86SF2	GALNT7	75.3	N-acetylgalactosaminyltransferase 7 OS	2	1	0.8630	0.8987
B1AJY7	PSMD10	20.8	26S proteasome non-ATPase regulatory subunit 10 OS	20	4	0.8620	0.7679
P02749	APOH	38.3	Beta-2-glycoprotein 1 OS	5	2	0.8620	0.5234
Q5TH30	NDRG3	42.8	N-myc downstream-regulated gene 3 protein OS	11	4	0.8620	0.3919
Q9BUH6	PAXX	21.6	Protein PAXX OS	16	3	0.8620	0.7128
H3BNW0	THUMPD1	15.8	THUMP domain-containing protein 1 OS	10	1	0.8620	0.8474
O43852	CALU	37.1	Calumenin OS	19	5	0.8610	0.6499
Q9Y5B9	SUPT16H	119. 8	FACT complex subunit SPT16 OS	15	13	0.8610	0.3900
Q14517	FAT1	506	Protocadherin Fat 1 OS	1	2	0.8610	0.8613
A0A0J9Y X34	TRIM2	58.4	Tripartite motif-containing protein 2 OS	2	1	0.8610	0.6521
P19388	POLR2E	24.5	DNA-directed RNA polymerases I, II, and III subunit RPABC1 OS	17	3	0.8600	0.8244
Q16831	UPP1	33.9	Uridine phosphorylase 1 OS	18	5	0.8600	0.4854
A0A140T A32	C4A	187. 6	C4a anaphylatoxin OS	3	4	0.8590	0.7290
B7WPJ3	CASTOR1	32.2	Cytosolic arginine sensor for mTORC1 subunit 1 OS	2	1	0.8590	0.7798
Q9Y295	DRG1	40.5	Developmentally-regulated GTP-binding protein 1 OS	10	3	0.8590	0.8344
A0A087W W43	ITIH3	75	Inter-alpha-trypsin inhibitor heavy chain H3 OS	10	7	0.8590	0.4776
P30405	PPIF	22	Peptidyl-prolyl cis-trans isomerase F, mitochondrial OS	60	8	0.8590	0.3806
Q7Z6M1	RABEPK	40.5	Rab9 effector protein with kelch motifs OS	22	5	0.8590	0.7049
Q9BW04	C1orf116	63.9	Specifically androgen-regulated gene protein OS	37	12	0.8590	0.5119
Q14247	CTTN	61.5	Src substrate cortactin OS	33	13	0.8580	0.3768
Q9H4G0-2	EPB41L1	87.6	Isoform 2 of Band 4.1-like protein 1 OS	8	5	0.8570	0.7437
Q9HB07	MYG1	42.5	MYG1 exonuclease OS	25	8	0.8570	0.6573
O43815	STRN	86.1	Striatin OS	4	2	0.8570	0.8632
Q9BTC0	DIDO1	243. 7	Death-inducer obliterator 1 OS	1	2	0.8560	0.6340
Q15276	RABEP1	99.2	Rab GTPase-binding effector protein 1 OS	4	3	0.8560	0.8332
P19022	CDH2	99.7	Cadherin-2 OS	17	8	0.8550	0.6061
Q9H6F5	CCDC86	40.2	Coiled-coil domain-containing protein 86 OS	13	2	0.8550	0.8356
Q7Z417	NUFIP2	76.1	Nuclear fragile X mental retardation-interacting protein 2 OS	3	2	0.8540	0.7446
Q14232	EIF2B1	33.7	Translation initiation factor eIF-2B subunit alpha OS	5	1	0.8540	0.7975
K7EQY5	MATK	56.3	Tyrosine-protein kinase OS	2	1	0.8540	0.8742
P29692-3	EEF1D	28.5	Isoform 3 of Elongation factor 1-delta OS	60	22	0.8530	0.7335

P50281	MMP14	65.9	Matrix metalloproteinase-14 OS	14	6	0.8530	0.6277
O43617	TRAPPC3	20.3	Trafficking protein particle complex subunit 3 OS	23	4	0.8530	0.7730
C9JF17	APOD	24.1	Apolipoprotein D (Fragment) OS	3	1	0.8520	0.6531
Q13151	HNRNPA0	30.8	Heterogeneous nuclear ribonucleoprotein A0 OS	6	2	0.8520	0.7339
Q99460	PSMD1	105.8	26S proteasome non-ATPase regulatory subunit 1 OS	9	6	0.8510	0.5838
Q13200	PSMD2	100.1	26S proteasome non-ATPase regulatory subunit 2 OS	12	6	0.8510	0.5433
Q9H223	EHD4	61.1	EH domain-containing protein 4 OS	9	4	0.8510	0.8058
Q6P2Q9	PRPF8	273.4	Pre-mRNA-processing-splicing factor 8 OS	9	14	0.8510	0.7407
F5H6N1	MAGOHB	10.7	Protein mago nashi homolog 2 OS	80	9	0.8510	0.8219
P04350	TUBB4A	49.6	Tubulin beta-4A chain OS	61	29	0.8510	0.4594
Q15018	FAM175B	46.9	BRISC complex subunit Abraxas 2 OS	5	2	0.8500	0.3470
P24941	CDK2	33.9	Cyclin-dependent kinase 2 OS	38	7	0.8490	0.6495
P32455	GBP1	67.9	Guanylate-binding protein 1 OS	9	5	0.8480	0.6500
I3L1P5	HGS	30	Hepatocyte growth factor-regulated tyrosine kinase substrate (Fragment) OS	4	1	0.8470	0.8681
C9JTS3	AAMP	22	Angio-associated migratory cell protein (Fragment) OS	6	1	0.8460	0.8065
A0A0B4J2D5	C21orf33	28.1	Glutamine amidotransferase-like class 1 domain-containing protein 3B, mitochondrial OS	13	2	0.8460	0.8344
P06702	S100A9	13.2	Protein S100-A9 OS	66	8	0.8460	0.3286
P30626	SRI	21.7	Sorcin OS	30	6	0.8460	0.4556
P42574	CASP3	31.6	Caspase-3 OS	8	2	0.8450	0.6613
A0A075B7B1	SYNM	140.1	Desmuslin, isoform CRA_a OS	1	1	0.8440	0.4591
O60832	DKC1	57.6	H/ACA ribonucleoprotein complex subunit DKC1 OS	5	2	0.8440	0.8470
E7EWE1	UBA5	38.7	UFM1-activating enzyme OS	8	2	0.8440	0.8474
A0A1W2PNV4	N/A	75.8	Uncharacterized protein OS	21	7	0.8440	0.8344
I3L3I9	ALDH3A1	41.8	Aldehyde dehydrogenase, dimeric NADP-preferring OS	3	1	0.8430	0.8821
Q9NRF9	POLE3	16.8	DNA polymerase epsilon subunit 3 OS	45	5	0.8430	0.3173
Q9BSH5	HDHD3	28	Haloacid dehalogenase-like hydrolase domain-containing protein 3 OS	5	1	0.8430	0.8852
Q5T8P6	RBM26	113.5	RNA-binding protein 26 OS	1	1	0.8430	0.8659
Q02878	RPL6	32.7	60S ribosomal protein L6 OS	21	7	0.8410	0.4098
P46087	NOP2	89.2	Probable 28S rRNA (cytosine(4447)-C(5))-methyltransferase OS	4	3	0.8410	0.7181
P51665	PSMD7	37	26S proteasome non-ATPase regulatory subunit 7 OS	17	3	0.8400	0.6993
P62854	RPS26	13	40S ribosomal protein S26 OS	34	3	0.8400	0.5871
Q13045	FLII	144.7	Protein flightless-1 homolog OS	4	5	0.8400	0.8363
O43505	B3GNT1	47.1	Beta-1,4-glucuronyltransferase 1 OS	16	3	0.8380	0.7431
P22626-2	HNRNPA2B1	36	Isoform A2 of Heterogeneous nuclear ribonucleoproteins A2/B1 OS	84	46	0.8380	0.3005
Q9UBL3	ASH2L	68.7	Set1/Ash2 histone methyltransferase complex subunit ASH2 OS	9	3	0.8380	0.8607
O95848	NUDT14	24.1	Uridine diphosphate glucose pyrophosphatase NUDT14 OS	12	2	0.8380	0.8111
P39023	RPL3	46.1	60S ribosomal protein L3 OS	15	5	0.8370	0.4786
P34059	GALNS	58	N-acetylgalactosamine-6-sulfatase OS	3	1	0.8370	0.8734
P06396	GSN	85.6	Gelsolin OS	52	29	0.8360	0.2920
A0A1B0GVH5	FTO	64.1	Alpha-ketoglutarate-dependent dioxygenase FTO OS	12	4	0.8350	0.7527
Q9NUQ3	TXLNG	60.5	Gamma-taxilin OS	2	1	0.8350	0.5387
P14854	COX6B1	10.2	Cytochrome c oxidase subunit 6B1 OS	97	7	0.8340	0.2829
E5RI33	LY6E	7.1	Lymphocyte antigen 6E OS	15	1	0.8340	0.8051
O75643	SNRNP200	244.4	U5 small nuclear ribonucleoprotein 200 kDa helicase OS	11	15	0.8340	0.4740
P50914	RPL14	23.4	60S ribosomal protein L14 OS	6	2	0.8330	0.6308

Q9BRP8	WIBG	22.6	Partner of Y14 and mago OS	50	6	0.8320	0.5228
F8VZQ9	SARNP	24.1	SAP domain-containing ribonucleoprotein OS	15	4	0.8320	0.4193
Q6YP21	KYAT3	51.4	Kynurenine--oxoglutarate transaminase 3 OS	12	4	0.8280	0.7760
Q9UKF6	CPSF3	77.4	Cleavage and polyadenylation specificity factor subunit 3 OS	1	1	0.8270	0.8588
Q8IVM0	CCDC50	35.8	Coiled-coil domain-containing protein 50 OS	4	1	0.8260	0.8271
E7EQ69	NAA50	19.3	N-alpha-acetyltransferase 50 OS	39	5	0.8260	0.4591
P61956	SUMO2	10.9	Small ubiquitin-related modifier 2 OS	82	5	0.8260	0.2549
O75096	LRP4	211.9	Low-density lipoprotein receptor-related protein 4 OS	1	1	0.8250	0.3055
H0YKD8	RPL28	19.1	60S ribosomal protein L28 OS	6	1	0.8240	0.7148
P40429	RPL13A	23.6	60S ribosomal protein L13a OS	14	3	0.8230	0.7798
P19367	HK1	102.4	Hexokinase-1 OS	10	9	0.8230	0.3912
Q9NTM9	CUTC	29.3	Copper homeostasis protein cutC homolog OS	11	2	0.8220	0.7616
Q16610	ECM1	60.6	Extracellular matrix protein 1 OS	51	19	0.8220	0.2436
Q9H074-3	PAIP1	42	Isoform 3 of Polyadenylate-binding protein-interacting protein 1 OS	13	3	0.8220	0.7724
P31639	SLC5A2	72.8	Sodium/glucose cotransporter 2 OS	1	1	0.8220	0.2438
P32926	DSG3	107.5	Desmoglein-3 OS	18	11	0.8210	0.2528
Q5Y7D1	HLA-DRB1	30.1	HLA class II histocompatibility antigen DR beta chain OS	9	1	0.8210	0.7699
I3L303	RPS15A	6	40S ribosomal protein S15a OS	21	2	0.8190	0.5208
H3BQZ9	APRT	16.6	Adenine phosphoribosyltransferase OS	41	4	0.8180	0.7231
O94907	DKK1	28.7	Dickkopf-related protein 1 OS	25	7	0.8180	0.3353
Q15007	WTAP	44.2	Pre-mRNA-splicing regulator WTAP OS	16	4	0.8180	0.7798
O00743	PPP6C	35.1	Serine/threonine-protein phosphatase 6 catalytic subunit OS	12	3	0.8180	0.6567
M0R2K3	BABAM1	21.8	BRISC and BRCA1-A complex member 1 (Fragment) OS	17	2	0.8160	0.7527
P33993	MCM7	81.3	DNA replication licensing factor MCM7 OS	20	9	0.8150	0.6531
O15118	NPC1	142.1	NPC intracellular cholesterol transporter 1 OS	1	1	0.8150	0.8585
Q5VY30	RBP4	23	Retinol-binding protein OS	11	3	0.8150	0.3228
P19971	TYMP	49.9	Thymidine phosphorylase OS	46	15	0.8150	0.2216
P50402	EMD	29	Emerin OS	23	4	0.8130	0.8585
Q6PKG0	LARP1	123.4	La-related protein 1 OS	11	5	0.8130	0.6406
Q96QR8	PURB	33.2	Transcriptional activator protein Pur-beta OS	35	9	0.8130	0.4117
P08727	KRT19	44.1	Keratin, type I cytoskeletal 19 OS	59	24	0.8120	0.2134
Q5T749	KPRP	64.1	Keratinocyte proline-rich protein OS	5	2	0.8110	0.7746
Q9H0H5	RACGAP1	71	Rac GTPase-activating protein 1 OS	3	1	0.8110	0.8129
E9PDQ5	ARHGEF7	61.6	Rho guanine nucleotide exchange factor 7 OS	2	1	0.8110	0.8549
Q9Y2W2	WBP11	70	WW domain-binding protein 11 OS	7	3	0.8110	0.4632
P13807	GYS1	83.7	Glycogen [starch] synthase, muscle OS	5	2	0.8100	0.8344
Q15651	HMGN3	10.7	High mobility group nucleosome-binding domain-containing protein 3 OS	16	2	0.8100	0.4002
Q14696	MESDC2	26.1	LRP chaperone MESD OS	10	2	0.8100	0.6406
I3L3H2	EIF4A3	44.5	RNA helicase OS	34	14	0.8100	0.4346
Q9UH65	SWAP70	69	Switch-associated protein 70 OS	11	6	0.8090	0.2032
H0YBS3	BPNT2	9.3	Golgi-resident adenosine 3',5'-bisphosphate 3'-phosphatase (Fragment) OS	7	1	0.8080	0.8474
Q6EEV4-2	POLR2M	8.8	Isoform 5 of DNA-directed RNA polymerase II subunit GRINL1A, isoforms 4/5 OS	29	2	0.8080	0.8050
P98160	HSPG2	468.5	Basement membrane-specific heparan sulfate proteoglycan core protein OS	2	5	0.8070	0.6340
A0A0C4DGB5	CAST	81	Calpain inhibitor OS	41	22	0.8070	0.8358
Q96EW2	HSPBAP1	55.1	HSPB1-associated protein 1 OS	1	1	0.8060	0.8344

P10606	COX5B	13.7	Cytochrome c oxidase subunit 5B, mitochondrial OS	29	2	0.8050	0.5194
F5H7N8	OLR1	24.5	Oxidized low-density lipoprotein receptor 1 (Fragment) OS	19	3	0.8050	0.3933
Q3YEC7	RABL6	79.5	Rab-like protein 6 OS	10	5	0.8050	0.3754
Q9H1E1	RNASE7	17.4	Ribonuclease 7 OS	10	1	0.8050	0.8424
Q96RL7	VPS13A	360	Vacuolar protein sorting-associated protein 13A OS	0	1	0.8050	0.1916
Q99879	HIST1H2BM	14	Histone H2B type 1-M OS	83	23	0.8040	0.1879
A0A494C 1A0	ACTN2	99.7	Alpha-actinin-2 (Fragment) OS	17	20	0.8030	0.3470
P25774	CTSS	37.5	Cathepsin S OS	12	3	0.8020	0.6362
Q9NYV4	CDK12	164. 1	Cyclin-dependent kinase 12 OS	1	1	0.8020	0.8461
H0YBR2	ESRP1	56.1	Epithelial-splicing regulatory protein 1 (Fragment) OS	1	1	0.8010	0.7760
Q8N1F7	NUP93	93.4	Nuclear pore complex protein Nup93 OS	4	3	0.8010	0.7826
P61224	RAP1B	20.8	Ras-related protein Rap-1b OS	21	3	0.8010	0.7837
O94804	STK10	112. 1	Serine/threonine-protein kinase 10 OS	5	2	0.8010	0.7812
Q13573	SNW1	61.5	SNW domain-containing protein 1 OS	16	4	0.8010	0.7049
P07205	PGK2	44.8	Phosphoglycerate kinase 2 OS	17	7	0.8000	0.8264
P62913	RPL11	20.2	60S ribosomal protein L11 OS	31	5	0.7990	0.2649
J3KP15	SRSF2	15.4	Serine/arginine-rich splicing factor 2 (Fragment) OS	38	4	0.7990	0.5192
A0A2U3T ZM0	CHD4	216. 7	DNA helicase OS	2	4	0.7980	0.6750
F5GYW6	XPOT	6.9	Exportin-T (Fragment) OS	48	3	0.7980	0.7975
P06748	NPM1	32.6	Nucleophosmin OS	75	21	0.7980	0.1726
P28347	TEAD1	47.9	Transcriptional enhancer factor TEF-1 OS	3	1	0.7980	0.3673
P45973	CBX5	22.2	Chromobox protein homolog 5 OS	37	5	0.7970	0.4464
P39060	COL18A1	178. 1	Collagen alpha-1(XVIII) chain OS	1	1	0.7970	0.8208
P23434	GCSH	18.9	Glycine cleavage system H protein, mitochondrial OS	38	5	0.7970	0.6061
P09234	SNRPC	17.4	U1 small nuclear ribonucleoprotein C OS	13	1	0.7970	0.7311
P52758	HRSP12	14.5	2-iminobutanoate/2-iminopropanoate deaminase OS	20	2	0.7960	0.3741
C9JN98	SERPINE2	30.4	Glia-derived nexin (Fragment) OS	13	2	0.7960	0.7702
P09341	CXCL1	11.3	Growth-regulated alpha protein OS	27	3	0.7960	0.4907
C9JW52	IGFBP2	8.7	Insulin-like growth factor-binding protein 2 (Fragment) OS	17	1	0.7960	0.7934
Q9H3U1-2	UNC45A	101. 6	Isoform 2 of Protein unc-45 homolog A OS	8	4	0.7960	0.7969
O15067	PFAS	144. 6	Phosphoribosylformylglycinamidine synthase OS	26	20	0.7960	0.2001
Q8IZ83	ALDH16A1	85.1	Aldehyde dehydrogenase family 16 member A1 OS	22	11	0.7950	0.3806
Q9P2R6	RERE	172. 3	Arginine-glutamic acid dipeptide repeats protein OS	1	1	0.7950	0.8398
O15254	ACOX3	77.6	Peroxisomal acyl-coenzyme A oxidase 3 OS	3	2	0.7950	0.4537
Q5SRN1	CDC40	60.8	Pre-mRNA-processing factor 17 OS	3	1	0.7950	0.7437
P46778	RPL21	18.6	60S ribosomal protein L21 OS	28	3	0.7940	0.6766
I6LPK7	BAX	15.9	Apoptosis regulator BAX OS	8	1	0.7930	0.8112
O43837	IDH3B	42.2	Isocitrate dehydrogenase [NAD] subunit beta, mitochondrial OS	4	2	0.7920	0.7826
Q03701	CEBPZ	120. 9	CCAAT/enhancer-binding protein zeta OS	1	1	0.7910	0.5925
P35269	GTF2F1	58.2	General transcription factor IIF subunit 1 OS	17	7	0.7910	0.4327
P53985	SLC16A1	53.9	Monocarboxylate transporter 1 OS	4	1	0.7910	0.7729
Q9NR30	DDX21	87.3	Nucleolar RNA helicase 2 OS	8	4	0.7910	0.5988
O43598	DNPH1	19.1	2'-deoxynucleoside 5'-phosphate N-hydrolase 1 OS	30	3	0.7900	0.3748
Q9NVZ3	NECAP2	28.3	Adaptin ear-binding coat-associated protein 2 OS	7	1	0.7900	0.7872
Q53LP3	SOWAHC	55.6	Ankyrin repeat domain-containing protein SOWAHC OS	4	1	0.7890	0.8282

P13716	ALAD	36.3	Delta-aminolevulinic acid dehydratase OS	27	6	0.7880	0.3127
P02533	KRT14	51.5	Keratin, type I cytoskeletal 14 OS	61	28	0.7880	0.1469
P61081	UBE2M	20.9	NEDD8-conjugating enzyme Ubc12 OS	31	7	0.7880	0.2137
Q9BYX7	POTEKP	42	Putative beta-actin-like protein 3 OS	32	23	0.7880	0.8344
E9PI99	NFYC	16.2	Nuclear transcription factor Y subunit gamma (Fragment) OS	24	3	0.7870	0.6161
A0A087WTG3	CUL3	39.1	Cullin-3 OS	3	1	0.7860	0.8158
C9JDU0	TSSC4	11.5	Protein TSSC4 (Fragment) OS	20	3	0.7860	0.7213
Q92597	NDRG1	42.8	Protein NDRG1 OS	23	6	0.7840	0.1370
P14859	POU2F1	76.4	POU domain, class 2, transcription factor 1 OS	3	1	0.7830	0.8289
P26373	RPL13	24.2	60S ribosomal protein L13 OS	37	9	0.7820	0.1325
Q9NTU7	CBLN4	21.8	Cerebellin-4 OS	3	1	0.7820	0.7181
P54802	NAGLU	82.2	Alpha-N-acetylglucosaminidase OS	5	3	0.7800	0.8263
O95864	FADS2	52.2	Acyl-CoA 6-desaturase OS	2	1	0.7790	0.7575
P51991-2	HNRNPA3	37	Isoform 2 of Heterogeneous nuclear ribonucleoprotein A3 OS	38	9	0.7790	0.7317
Q96RS6	NUDCD1	66.7	NudC domain-containing protein 1 OS	3	2	0.7790	0.7080
A0A0G2JHC2	PPP1R18	68	PPP1R18 OS	2	1	0.7790	0.7837
F8WF17	LGALSL	8.1	Galectin-related protein OS	13	1	0.7780	0.7987
H0YJM2	UBR7	20.5	Putative E3 ubiquitin-protein ligase UBR7 (Fragment) OS	13	1	0.7780	0.8001
P07357	C8A	65.1	Complement component C8 alpha chain OS	2	1	0.7770	0.7992
P04792	HSPB1	22.8	Heat shock protein beta-1 OS	82	11	0.7770	0.1203
P22307	SCP2	59	Non-specific lipid-transfer protein OS	6	3	0.7770	0.4594
Q9NT62	ATG3	35.8	Ubiquitin-like-conjugating enzyme ATG3 OS	10	2	0.7760	0.7527
P31944	CASP14	27.7	Caspase-14 OS	7	1	0.7750	0.8223
A0A0G2JNJ7	CNOT3	39.5	CCR4-NOT transcription complex subunit 3 (Fragment) OS	6	3	0.7750	0.4800
C9IZ93	UBE2F	18.6	NEDD8-conjugating enzyme UBE2F OS	6	1	0.7740	0.3892
Q15814	TBCC	39.2	Tubulin-specific chaperone C OS	2	1	0.7730	0.7942
P52895	AKR1C2	36.7	Aldo-keto reductase family 1 member C2 OS	81	32	0.7720	0.1099
A0A5F9ZHB6	PRIM1	54	DNA primase OS	3	1	0.7720	0.8187
Q8WW12	PCNP	18.9	PEST proteolytic signal-containing nuclear protein OS	58	9	0.7710	0.1059
Q15654	TRIP6	50.3	Thyroid receptor-interacting protein 6 OS	28	5	0.7710	0.4391
O75396	SEC22B	24.6	Vesicle-trafficking protein SEC22b OS	24	3	0.7710	0.5740
Q14978-3	NOLC1	73.7	Isoform 3 of Nucleolar and coiled-body phosphoprotein 1 OS	18	10	0.7700	0.1975
E5RHK8	DNM3	72.8	Dynamin GTPase OS	4	2	0.7690	0.8165
O75400	PRPF40A	108.7	Pre-mRNA-processing factor 40 homolog A OS	1	1	0.7690	0.8165
P23258	TUBG1	51.1	Tubulin gamma-1 chain OS	5	2	0.7690	0.7561
A8MUM1	TSSC1	46.3	EARP and GARP complex-interacting protein 1 OS	4	1	0.7680	0.7114
O75718	CRTAP	46.5	Cartilage-associated protein OS	2	1	0.7670	0.8058
P10412	HIST1H1E	21.9	Histone H1.4 OS	39	15	0.7670	0.0987
E9PNY1	ZFPL1	22.7	Zinc finger protein-like 1 (Fragment) OS	4	1	0.7670	0.6406
Q9NYJ1	COA4	10.1	Cytochrome c oxidase assembly factor 4 homolog, mitochondrial OS	36	2	0.7660	0.6737
P01023	A2M	163.2	Alpha-2-macroglobulin OS	4	8	0.7650	0.0945
Q92769	HDAC2	55.3	Histone deacetylase 2 OS	16	6	0.7640	0.1821
Q96DI7	SNRNP40	39.3	U5 small nuclear ribonucleoprotein 40 kDa protein OS	6	1	0.7640	0.8051
P69905	HBA2	15.2	Hemoglobin subunit alpha OS	33	4	0.7630	0.1629
J3KQN4	RPL36A	16.4	60S ribosomal protein L36a OS	9	1	0.7620	0.7477

H3BUZ9	MPI	37.7	Mannose-6-phosphate isomerase OS	11	2	0.7620	0.7684
G3V3R7	ATXN3	37.7	Ubiquitinyl hydrolase 1 (Fragment) OS	10	2	0.7620	0.7494
A0A2R8Y811	RPS14	16.1	40S ribosomal protein S14 (Fragment) OS	43	7	0.7610	0.1427
Q96PP4	TSGA13	31.8	Testis-specific gene 13 protein OS	6	1	0.7610	0.2341
A0A087WTA8	COL1A2	129.1	Collagen alpha-2(I) chain OS	1	1	0.7600	0.7798
Q9Y2U8	LEMD3	99.9	Inner nuclear membrane protein Man1 OS	5	2	0.7580	0.7724
Q9UN86	G3BP2	54.1	Ras GTPase-activating protein-binding protein 2 OS	12	6	0.7570	0.4479
H7BXY3	DHX30	130.5	RNA helicase OS	1	1	0.7570	0.8051
A6NML8	DIAPH2	124.8	Diaphanous homolog 2 (Drosophila), isoform CRA_c OS	1	1	0.7560	0.7987
Q07666	KHDRBS1	48.2	KH domain-containing, RNA-binding, signal transduction-associated protein 1 OS	34	9	0.7560	0.0870
Q8WWM7	ATXN2L	113.3	Ataxin-2-like protein OS	7	3	0.7540	0.3977
A0A0G2JHL0	BRD2	67.2	Bromodomain-containing protein 2 (Fragment) OS	1	1	0.7540	0.7992
Q9BV57	ADI1	21.5	1,2-dihydroxy-3-keto-5-methylthiopentene dioxygenase OS	17	2	0.7530	0.7311
A0A0A0MRB5	MADD	176.4	MAP kinase-activating death domain protein OS	1	1	0.7530	0.5414
Q9NRX4	PHPT1	13.8	14 kDa phosphohistidine phosphatase OS	86	8	0.7500	0.0795
K7EN66	CNP	13.6	2',3'-cyclic-nucleotide 3'-phosphodiesterase (Fragment) OS	5	1	0.7500	0.7798
Q15642	TRIP10	68.3	Cdc42-interacting protein 4 OS	2	1	0.7500	0.7437
Q01780	EXOSC10	100.8	Exosome component 10 OS	2	1	0.7500	0.7987
P62266	RPS23	15.8	40S ribosomal protein S23 OS	45	5	0.7490	0.2335
P00734	F2	70	Prothrombin OS	4	2	0.7490	0.2267
O75688	PPM1B	52.6	Protein phosphatase 1B OS	7	3	0.7480	0.5761
P84098	RPL19	23.5	60S ribosomal protein L19 OS	24	6	0.7470	0.1843
Q92990	GLMN	68.2	Glomulin OS	2	1	0.7430	0.7870
A8MTL9	SERPINB8	15.3	Serpin-like protein HMSD OS	11	2	0.7410	0.7875
Q92466	DDB2	47.8	DNA damage-binding protein 2 OS	20	5	0.7400	0.3386
P28799	GRN	63.5	Progranulin OS	34	15	0.7400	0.0555
O15027	SEC16A	251.7	Protein transport protein Sec16A OS	8	9	0.7390	0.2141
P16401	HIST1H1B	22.6	Histone H1.5 OS	38	14	0.7380	0.0517
F5H7W8	C12orf43	31.7	Protein CUSTOS OS	5	1	0.7380	0.7782
F8WDK3	PPP4R2	7.3	Serine/threonine-protein phosphatase 4 regulatory subunit 2 (Fragment) OS	44	2	0.7380	0.6495
A0A2R8Y688	HADHA	39.2	Trifunctional enzyme subunit alpha, mitochondrial OS	2	1	0.7380	0.7437
Q9GZS3	WDR61	33.6	WD repeat-containing protein 61 OS	4	1	0.7380	0.6923
Q01082-3	SPTBN1	251.2	Isoform 2 of Spectrin beta chain, non-erythrocytic 1 OS	31	54	0.7360	0.6340
Q15291	RBBP5	59.1	Retinoblastoma-binding protein 5 OS	2	1	0.7360	0.7837
Q86UD1	OAF	30.7	Out at first protein homolog OS	4	1	0.7350	0.7383
A0A0C4DG89	DDX46	117.4	RNA helicase OS	2	2	0.7350	0.6453
P17028	ZNF24	42.1	Zinc finger protein 24 OS	3	1	0.7350	0.7761
A0A087WT80	PLCB1	129.6	1-phosphatidylinositol 4,5-bisphosphate phosphodiesterase OS	1	1	0.7320	0.5137
P26640	VARS	140.4	Valine--tRNA ligase OS	6	5	0.7320	0.1939
P13984	GTF2F2	28.4	General transcription factor IIF subunit 2 OS	17	3	0.7310	0.4143
P16278	GLB1	76	Beta-galactosidase OS	2	1	0.7300	0.6406
Q96AG4	LRRC59	34.9	Leucine-rich repeat-containing protein 59 OS	27	7	0.7300	0.3470
O14908	GIPC1	36	PDZ domain-containing protein GIPC1 OS	6	2	0.7290	0.7097
Q71DI3	HIST2H3A	15.4	Histone H3.2 OS	66	23	0.7280	0.5234

P35580-4	MYH10	232.4	Isoform 4 of Myosin-10 OS	6	9	0.7280	0.7725
Q9UBV4	WNT16	40.7	Protein Wnt-16 OS	2	1	0.7270	0.6604
P62891	RPL39	6.4	60S ribosomal protein L39 OS	24	2	0.7250	0.7317
O75843	AP1G2	87.1	AP-1 complex subunit gamma-like 2 OS	1	1	0.7250	0.7735
Q9Y5J7	TIMM9	10.4	Mitochondrial import inner membrane translocase subunit Tim9 OS	52	3	0.7240	0.1676
Q14151	SAFB2	107.4	Scaffold attachment factor B2 OS	15	9	0.7240	0.1123
A0A0G2JRQ5	GSTT1	24.7	Glutathione transferase OS	4	1	0.7220	0.7722
J3KT25	IMPACT	20.5	Protein IMPACT (Fragment) OS	17	1	0.7220	0.7334
P03973	SLPI	14.3	Antileukoproteinase OS	56	13	0.7210	0.0348
O60637	TSPAN3	28	Tetraspanin-3 OS	3	1	0.7190	0.3900
Q9NQX3	GPHN	79.7	Gephyrin OS	8	3	0.7150	0.3048
E9PC90	CCNB1	44.9	G2/mitotic-specific cyclin-B1 (Fragment) OS	4	1	0.7130	0.7256
P05109	S100A8	10.8	Protein S100-A8 OS	52	8	0.7130	0.0297
Q96B26	EXOSC8	30	Exosome complex component RRP43 OS	14	2	0.7120	0.6520
Q92620	DHX38	140.4	Pre-mRNA-splicing factor ATP-dependent RNA helicase PRP16 OS	3	2	0.7110	0.7025
P09238	MMP10	54.1	Stromelysin-2 OS	8	3	0.7100	0.5778
Q01658	DR1	19.4	Protein Dr1 OS	28	4	0.7090	0.2528
O43567	RNF13	42.8	E3 ubiquitin-protein ligase RNF13 OS	9	2	0.7070	0.4073
P62072	TIMM10	10.3	Mitochondrial import inner membrane translocase subunit Tim10 OS	27	1	0.7060	0.7494
Q01081	U2AF1	27.9	Splicing factor U2AF 35 kDa subunit OS	15	2	0.7060	0.7245
O43264	ZW10	88.8	Centromere/kinetochore protein zw10 homolog OS	3	1	0.7050	0.7383
A0A2R8Y6W5	MEA1	20.4	Male-enhanced antigen 1 OS	16	2	0.7050	0.6521
P07498	CSN3	20.3	Kappa-casein OS	4	1	0.7040	0.0261
Q14195	DPYSL3	61.9	Dihydropyrimidinase-related protein 3 OS	13	6	0.7030	0.5943
P36954	POLR2I	14.5	DNA-directed RNA polymerase II subunit RPB9 OS	6	1	0.7030	0.6848
Q9UBB4	ATXN10	53.5	Ataxin-10 OS	8	2	0.7010	0.7469
Q7Z3T8	ZFYVE16	168.8	Zinc finger FYVE domain-containing protein 16 OS	0	1	0.7010	0.7056
Q86W42	THOC6	37.5	THO complex subunit 6 homolog OS	6	1	0.7000	0.7437
Q8N3D4	EHBP1L1	161.8	EH domain-binding protein 1-like protein 1 OS	1	1	0.6990	0.6945
I3L3I6	NMRAL1	24.5	NmrA-like family domain-containing protein 1 (Fragment) OS	3	1	0.6990	0.6398
P04040	CAT	59.7	Catalase OS	20	6	0.6970	0.1185
Q9BZM5	ULBP2	27.4	UL16-binding protein 2 OS	16	2	0.6970	0.4528
P62280	RPS11	18.4	40S ribosomal protein S11 OS	53	9	0.6960	0.0300
P68431	HIST1H3F	15.4	Histone H3.1 OS	66	24	0.6960	0.0176
M0R0C3	TIMM50	12	Mitochondrial import inner membrane translocase subunit TIM50 (Fragment) OS	16	1	0.6940	0.7446
P08697	SERPINF2	54.5	Alpha-2-antiplasmin OS	5	3	0.6930	0.0156
Q96I25	RBM17	44.9	Splicing factor 45 OS	2	1	0.6930	0.4411
P29966	MARCKS	31.5	Myristoylated alanine-rich C-kinase substrate OS	23	8	0.6920	0.0153
O15160	POLR1C	39.2	DNA-directed RNA polymerases I and III subunit RPAC1 OS	14	3	0.6910	0.6841
Q9UIW2	PLXNA1	210.9	Plexin-A1 OS	1	2	0.6860	0.6340
P09622	DLD	54.1	Dihydrolipoyl dehydrogenase, mitochondrial OS	31	9	0.6830	0.0407
A0A2U3TZV8	PLCH1	188.6	Phosphoinositide phospholipase C OS	0	1	0.6830	0.6774
Q9NYU2	UGGT1	177.1	UDP-glucose:glycoprotein glucosyltransferase 1 OS	3	3	0.6830	0.5302
Q14061	COX17	6.9	Cytochrome c oxidase copper chaperone OS	46	1	0.6820	0.6117
P20061	TCN1	48.2	Transcobalamin-1 OS	3	1	0.6820	0.6239

O75144	ICOSLG	33.3	ICOS ligand OS	3	1	0.6780	0.6427
A0A0U1RQV4	ROCK1	133	Non-specific serine/threonine protein kinase OS	3	2	0.6750	0.7167
Q16651	PRSS8	36.4	Prostasin OS	9	2	0.6740	0.5800
P21912	SDHB	31.6	Succinate dehydrogenase [ubiquinone] iron-sulfur subunit, mitochondrial OS	3	1	0.6720	0.0685
Q709C8	VPS13C	422.1	Vacuolar protein sorting-associated protein 13C OS	0	1	0.6720	0.0685
A0A0A0MQS3	C19orf53	8.9	Leydig cell tumor 10 kDa protein homolog (Fragment) OS	9	1	0.6700	0.6117
Q96RI1	NR1H4	55.9	Bile acid receptor OS	1	1	0.6690	0.4997
P08779	KRT16	51.2	Keratin, type I cytoskeletal 16 OS	46	23	0.6680	0.1151
Q12965	MYO1E	127	Unconventional myosin-Ie OS	1	2	0.6670	0.3977
P06576	ATP5B	56.5	ATP synthase subunit beta, mitochondrial OS	41	14	0.6660	0.0098
P78386	KRT85	55.8	Keratin, type II cuticular Hb5 OS	4	2	0.6660	0.2717
Q16706	MAN2A1	131.1	Alpha-mannosidase 2 OS	3	2	0.6650	0.6904
Q9UPU5	USP24	294.2	Ubiquitin carboxyl-terminal hydrolase 24 OS	1	2	0.6650	0.4789
Q86SJ2	AMIGO2	57.9	Amphoterin-induced protein 2 OS	5	2	0.6630	0.4573
P40306	PSMB10	28.9	Proteasome subunit beta type-10 OS	30	5	0.6620	0.0466
Q02880	TOP2B	183.2	DNA topoisomerase 2-beta OS	2	1	0.6590	0.6848
E5RJG0	FZD6	11.6	Frizzled-6 OS	17	1	0.6590	0.6332
Q9NY12	GAR1	22.3	H/ACA ribonucleoprotein complex subunit 1 OS	3	1	0.6590	0.6499
Q8N4P3	HDDC3	20.3	Guanosine-3',5'-bis(diphosphate) 3'-pyrophosphohydrolase MESH1 OS	10	1	0.6550	0.6332
Q9H2J4	PDCL3	27.6	Phosducin-like protein 3 OS	13	2	0.6530	0.6679
P55735	SEC13	35.5	Protein SEC13 homolog OS	8	1	0.6530	0.5564
E9PQR7	VPS28	18.5	Vacuolar protein sorting-associated protein 28 homolog (Fragment) OS	22	3	0.6520	0.4468
Q9NPH2	ISYNA1	61	Inositol-3-phosphate synthase 1 OS	13	6	0.6490	0.2540
A0A5F9ZHM4	LDHB	37.4	L-lactate dehydrogenase OS	18	6	0.6490	0.2087
K7ENR6	PSMG2	26.7	Proteasome assembly chaperone 2 OS	14	3	0.6490	0.1574
D7RIG5	HLA-DRB3	29.8	HLA class II histocompatibility antigen DR beta chain OS	5	2	0.6480	0.3634
C9J0X3	PDLIM2	18.1	PDZ and LIM domain protein 2 (Fragment) OS	9	1	0.6480	0.6651
Q16718	NDUFA5	13.5	NADH dehydrogenase [ubiquinone] 1 alpha subcomplex subunit 5 OS	9	1	0.6470	0.0034
Q15059	BRD3	79.5	Bromodomain-containing protein 3 OS	1	1	0.6460	0.5414
F5H5A1	ACSF3	35.1	Malonate--CoA ligase ACSF3, mitochondrial OS	3	1	0.6460	0.2220
P00747	PLG	90.5	Plasminogen OS	3	3	0.6460	0.2848
Q9NP58	ABCB6	93.8	ATP-binding cassette sub-family B member 6, mitochondrial OS	1	1	0.6450	0.0031
H3BPY5	CARHSP1	13.5	Calcium-regulated heat-stable protein 1 (Fragment) OS	65	4	0.6450	0.4769
A0A3B3IRN5	FMOD	32.7	Fibromodulin OS	5	1	0.6440	0.6632
B8ZZQ6	PTMA	11.8	Prothymosin alpha OS	19	3	0.6430	0.0054
Q9Y608	LRRFIP2	82.1	Leucine-rich repeat flightless-interacting protein 2 OS	2	1	0.6410	0.6101
Q9UIV8	SERPINB13	44.2	Serpin B13 OS	66	29	0.6400	0.0687
P13647	KRT5	62.3	Keratin, type II cytoskeletal 5 OS	42	29	0.6390	0.0024
O95302	FKBP9	63	Peptidyl-prolyl cis-trans isomerase FKBP9 OS	3	2	0.6390	0.2135
E7EQV9	RPL15	20.5	Ribosomal protein L15 (Fragment) OS	13	2	0.6390	0.1151
F8W543	NAP1L1	38.2	Nucleosome assembly protein 1-like 1 OS	65	18	0.6380	0.5194
Q00403	GTF2B	34.8	Transcription initiation factor IIB OS	7	1	0.6380	0.6596
Q02388	COL7A1	295	Collagen alpha-1(VII) chain OS	2	3	0.6340	0.0666
O60826	CCDC22	70.7	Coiled-coil domain-containing protein 22 OS	2	1	0.6290	0.6500
Q71UI9	H2AFV	13.5	Histone H2A.V OS	38	4	0.6290	0.3545

J3KP36	WASHC2C	137.9	WASH complex subunit 2C OS	5	4	0.6280	0.5980
Q8NHV1	GIMAP7	34.5	GTPase IMAP family member 7 OS	6	2	0.6270	0.0290
P01042	KNG1	71.9	Kininogen-1 OS	1	1	0.6260	0.2464
J3KMX3	AFP	70.4	Alpha-fetoprotein OS	11	8	0.6250	0.0014
P17066	HSPA6	71	Heat shock 70 kDa protein 6 OS	21	15	0.6240	0.5205
Q16629	SRSF7	27.4	Serine/arginine-rich splicing factor 7 OS	11	2	0.6240	0.4591
P47929	LGALS7	15.1	Galectin-7 OS	40	3	0.6230	0.0292
P25705	ATP5A1	59.7	ATP synthase subunit alpha, mitochondrial OS	8	3	0.6220	0.1701
Q53GS9	USP39	65.3	U4/U6.U5 tri-snRNP-associated protein 2 OS	2	1	0.6220	0.5348
Q9UKV3	ACIN1	151.8	Apoptotic chromatin condensation inducer in the nucleus OS	10	8	0.6190	0.0010
E9PHY5	EPB41L2	104.3	Band 4.1-like protein 2 OS	6	3	0.6190	0.1125
Q5SRP5	APOM	14.2	Apolipoprotein M OS	5	1	0.6180	0.4767
H0YN48	UACA	63.9	Uveal autoantigen with coiled-coil domains and ankyrin repeats (Fragment) OS	2	1	0.6160	0.2755
P19012	KRT15	49.2	Keratin, type I cytoskeletal 15 OS	25	10	0.6110	0.1813
P48634	PRRC2A	228.7	Protein PRRC2A OS	2	2	0.6110	0.2851
Q13426	XRCC4	38.3	DNA repair protein XRCC4 OS	2	1	0.6100	0.4997
Q9Y2K3	MYH15	224.5	Myosin-15 OS	0	1	0.6100	0.0041
P19957	PI3	12.3	Elafin OS	49	5	0.6090	0.0007
Q969P0	IGSF8	65	Immunoglobulin superfamily member 8 OS	6	3	0.6090	0.5367
Q8N7H5	PAF1	59.9	RNA polymerase II-associated factor 1 homolog OS	5	2	0.6090	0.5967
O00160	MYO1F	124.8	Unconventional myosin-If OS	1	1	0.6080	0.4530
Q9HCN8	SDF2L1	23.6	Stromal cell-derived factor 2-like protein 1 OS	9	1	0.6070	0.4364
P49207	RPL34	13.3	60S ribosomal protein L34 OS	6	1	0.6050	0.3658
Q9UBF2	COPG2	97.6	Coatomer subunit gamma-2 OS	5	3	0.6050	0.5870
H3BU49	ARL2BP	13.8	ADP-ribosylation factor-like protein 2-binding protein OS	11	1	0.6030	0.4425
P35443	THBS4	105.8	Thrombospondin-4 OS	5	4	0.6020	0.0838
Q9C0C9	UBE2O	141.2	(E3-independent) E2 ubiquitin-conjugating enzyme OS	1	1	0.6000	0.5241
P16070	CD44	81.5	CD44 antigen OS	16	11	0.5970	0.0004
D6R9W4	DBN1	36.4	Drebrin (Fragment) OS	3	1	0.5970	0.3109
P15085	CPA1	47.1	Carboxypeptidase A1 OS	3	1	0.5960	0.3900
P81605	DCD	11.3	Dermcidin OS	75	5	0.5890	0.0008
Q9BXR0	QTRT1	44	Queuine tRNA-ribosyltransferase catalytic subunit 1 OS	7	2	0.5860	0.3446
H0YCU9	TAGLN	16.8	Transgelin (Fragment) OS	9	1	0.5850	0.2086
M0QZD8	LOC400499	357.6	Uncharacterized protein OS	1	1	0.5740	0.5192
Q9ULW0	TPX2	85.6	Targeting protein for Xklp2 OS	7	4	0.5720	0.0795
Q9Y4F5	CEP170B	171.6	Centrosomal protein of 170 kDa protein B OS	1	1	0.5690	0.5151
F8W9B8	EXOC5	74.3	Exocyst complex component 5 OS	2	1	0.5690	0.0128
Q14203	DCTN1	141.6	Dynactin subunit 1 OS	6	6	0.5670	0.0058
Q969E4	TCEAL3	22.5	Transcription elongation factor A protein-like 3 OS	17	2	0.5660	0.1260
Q9H2U1	DHX36	114.7	ATP-dependent DNA/RNA helicase DHX36 OS	1	1	0.5640	0.5030
Q6IA86	ELP2	92.4	Elongator complex protein 2 OS	1	1	0.5640	0.3330
P02452	COL1A1	138.9	Collagen alpha-1(I) chain OS	1	1	0.5610	0.2015
Q9UKV8	ago-02	97.1	Protein argonaute-2 OS	7	3	0.5610	0.4255
O75446	SAP30	23.3	Histone deacetylase complex subunit SAP30 OS	8	1	0.5600	0.0026
Q5T085	AMY1B	25.6	Alpha-amylase (Fragment) OS	9	2	0.5590	0.4327

Q8TDB6	DTX3L	83.5	E3 ubiquitin-protein ligase DTX3L OS	4	2	0.5570	0.2320
Q9H2D6	TRIOBP	261.2	TRIO and F-actin-binding protein OS	0	1	0.5570	0.1569
O95497	VNN1	57	Pantetheinase OS	2	1	0.5560	0.1165
Q8TEA7	TBCK	100.6	TBC domain-containing protein kinase-like protein OS	1	1	0.5550	0.0824
B4DP31	PRPSAP1	31.2	Phosphoribosyl pyrophosphate synthase-associated protein 1 OS	5	1	0.5450	0.1759
Q9Y5X9	LIPG	56.8	Endothelial lipase OS	3	1	0.5430	0.4920
O95361	TRIM16	63.9	Tripartite motif-containing protein 16 OS	6	2	0.5410	0.4591
P15090	FABP4	14.7	Fatty acid-binding protein, adipocyte OS	24	3	0.5370	0.0042
P16402	HIST1H1D	22.3	Histone H1.3 OS	39	14	0.5370	0.0645
C9JHK9	ABCF2	26.9	ATP-binding cassette sub-family F member 2 (Fragment) OS	11	2	0.5360	0.2901
P14780	MMP9	78.4	Matrix metalloproteinase-9 OS	7	4	0.5360	0.2148
Q9UDT6	CLIP2	115.8	CAP-Gly domain-containing linker protein 2 OS	2	2	0.5290	0.2218
H3BMU4	ZSCAN29	36.8	Zinc finger and SCAN domain-containing protein 29 (Fragment) OS	2	1	0.5240	0.0001
P35908	KRT2	65.4	Keratin, type II cytoskeletal 2 epidermal OS	68	37	0.5210	0.0000
Q9Y371	SH3GLB1	40.8	Endophilin-B1 OS	3	1	0.5190	0.2848
P02538	KRT6A	60	Keratin, type II cytoskeletal 6A OS	40	26	0.5150	0.0000
E9PNP3	AAMDC	15.6	Mth938 domain-containing protein OS	21	2	0.5130	0.1484
Q00653	NFKB2	96.7	Nuclear factor NF-kappa-B p100 subunit OS	1	1	0.5090	0.2479
Q5QPL9	RALY	24.7	RNA-binding protein Raly (Fragment) OS	3	1	0.5090	0.3806
P42262	GRIA2	98.8	Glutamate receptor 2 OS	1	1	0.5040	0.4097
X6RLX0	ERC1	128.4	ELKS/Rab6-interacting/CAST family member 1 OS	1	1	0.4980	0.1063
E9PR67	MTMR9	11.4	Myotubularin-related protein 9 OS	20	1	0.4880	0.3284
A0A087WTD7	AKAP13	161.1	A-kinase anchor protein 13 OS	1	1	0.4820	0.0869
P19827	ITIH1	101.3	Inter-alpha-trypsin inhibitor heavy chain H1 OS	1	2	0.4820	0.0030
P02794	FTH1	21.2	Ferritin heavy chain OS	85	16	0.4730	0.0000
Q5EBM0	CMPK2	49.4	UMP-CMP kinase 2, mitochondrial OS	7	2	0.4720	0.1499
Q9NZH8	IL36G	18.7	Interleukin-36 gamma OS	18	2	0.4710	0.2161
U3KQF3	TPI1	10.6	Triosephosphate isomerase (Fragment) OS	97	18	0.4690	0.0095
A0A0A0MS54	PRKACB	41.3	cAMP-dependent protein kinase catalytic subunit beta OS	6	2	0.4680	0.1099
Q5T200	ZC3H13	196.5	Zinc finger CCCH domain-containing protein 13 OS	1	1	0.4640	0.1787
A0A1W2PQG6	CSTB	6.2	Cystatin-B (Fragment) OS	39	1	0.4620	0.0013
K7EIJ2	SMAD4	15.9	Mothers against decapentaplegic homolog 4 (Fragment) OS	16	2	0.4450	0.0620
B3KNX7	PAK1	58.2	Non-specific serine/threonine protein kinase OS	8	3	0.4450	0.0760
P25311	AZGP1	34.2	Zinc-alpha-2-glycoprotein OS	4	1	0.4430	0.0637
P02748	C9	63.1	Complement component C9 OS	2	1	0.4420	0.2326
Q13131	PRKAA1	64	5'-AMP-activated protein kinase catalytic subunit alpha-1 OS	1	1	0.4340	0.2329
Q96Q11	TRNT1	50.1	CCA tRNA nucleotidyltransferase 1, mitochondrial OS	7	2	0.4340	0.1625
P08621	SNRNP70	51.5	U1 small nuclear ribonucleoprotein 70 kDa OS	2	1	0.4300	0.0231
E9PQ80	CHMP4A	24.2	Charged multivesicular body protein 4a (Fragment) OS	15	2	0.4240	0.0021
A0A087WW36	ACP6	16.3	Lysophosphatidic acid phosphatase type 6 (Fragment) OS	5	1	0.4160	0.1422
A0A024QZ42	PDCD6	14.4	HCG1985580, isoform CRA_c OS	9	1	0.4150	0.2039
P23634	ATP2B4	137.8	Plasma membrane calcium-transporting ATPase 4 OS	1	1	0.4120	0.0043
P07204	THBD	60.3	Thrombomodulin OS	4	1	0.4120	0.1372
P51884	LUM	38.4	Lumican OS	15	4	0.4100	0.0000
Q96KQ7	EHMT2	132.3	Histone-lysine N-methyltransferase EHMT2 OS	3	1	0.4070	0.2135

Q92626	PXDN	165.<br>2	Peroxidasin homolog OS	1	1	0.4000	0.0000
Q14508	WFDC2	13	WAP four-disulfide core domain protein 2 OS	49	3	0.3870	0.0104
P20073	ANXA7	52.7	Annexin A7 OS	6	1	0.3730	0.0489
E9PIP0	CHID1	13.4	Chitinase domain-containing protein 1 (Fragment) OS	14	1	0.3700	0.1254
P53367	ARFIP1	41.7	Arfaptin-1 OS	2	1	0.3640	0.0104
P62760	VSNL1	22.1	Visinin-like protein 1 OS	11	2	0.3590	0.0242
Q6P589	TNFAIP8L2	20.5	Tumor necrosis factor alpha-induced protein 8-like protein 2 OS	4	1	0.3540	0.0035
A0A2R8Y<br>6T2	SYNGAP1	144.<br>5	Ras/Rap GTPase-activating protein SynGAP OS	1	1	0.3460	0.0203
Q2TAA2	IAH1	27.6	Isoamyl acetate-hydrolyzing esterase 1 homolog OS	13	2	0.3320	0.0752
Q9Y3A5	SBDS	28.7	Ribosome maturation protein SBDS OS	14	3	0.3300	0.0001
A6NMB1	SIGLEC16	53	Sialic acid-binding Ig-like lectin 16 OS	1	1	0.3290	0.0139
O75909-1	CCNK	41.3	Isoform 3 of Cyclin-K OS	5	1	0.3280	0.0047
Q13155	AIMP2	35.3	Aminoacyl tRNA synthase complex-interacting multifunctional protein 2 OS	15	2	0.3250	0.0024
Q02413	DSG1	113.<br>7	Desmoglein-1 OS	6	4	0.3250	0.0001
Q8WZA9	IRGQ	62.7	Immunity-related GTPase family Q protein OS	1	1	0.3020	0.0354
Q04695	KRT17	48.1	Keratin, type I cytoskeletal 17 OS	24	12	0.3020	0.0067
P18510	IL1RN	20	Interleukin-1 receptor antagonist protein OS	7	1	0.3000	0.0073
Q96C36	PYCR2	33.6	Pyrroline-5-carboxylate reductase 2 OS	3	1	0.2850	0.0001
Q9BT09	CNPY3	30.7	Protein canopy homolog 3 OS	4	1	0.2720	0.0275
Q9GZQ8	MAP1LC3B	14.7	Microtubule-associated proteins 1A/1B light chain 3B OS	11	1	0.2640	0.0000
C9JAB9	NCK1	20.1	Cytoplasmic protein NCK1 (Fragment) OS	6	1	0.2560	0.0040
O75167	PHACTR2	69.7	Phosphatase and actin regulator 2 OS	3	1	0.2560	0.0162
E9PDW2	OXCT1	36.7	Succinyl-CoA:3-ketoacid coenzyme A transferase 1, mitochondrial OS	2	1	0.2500	0.0049
Q9P2W9	STX18	38.7	Syntaxin-18 OS	4	1	0.2450	0.0150
Q8IYH5	ZZZ3	102	ZZ-type zinc finger-containing protein 3 OS	1	1	0.2380	0.0000
K7EL65	IMPA2	12.7	Inositol monophosphatase 2 OS	16	2	0.2240	0.0000
C9JPE1	SLC25A20	25.1	Mitochondrial carnitine/acylcarnitine carrier protein OS	4	1	0.2100	0.0000
H0YLF3	B2M	8.5	Beta-2-microglobulin (Fragment) OS	100	15	0.2000	0.0038
D6RFL4	CD14	23.3	Monocyte differentiation antigen CD14 (Fragment) OS	8	1	0.1990	0.0000
P04259	KRT6B	60	Keratin, type II cytoskeletal 6B OS	35	24	0.1790	0.0000
P02808	STATH	7.3	Statherin OS	55	5	0.1660	0.0000
P11684	SCGB1A1	10	Uteroglobin OS	77	10	0.1610	0.0000
Q92523	CPT1B	87.7	Carnitine O-palmitoyltransferase 1, muscle isoform OS	2	1	0.1370	0.0000
P13674	P4HA1	61	Prolyl 4-hydroxylase subunit alpha-1 OS	3	1	0.1370	0.0000
P0DP57	LYNX1	10.2	Secreted Ly-6/uPAR domain-containing protein 2 OS	13	1	0.1350	0.0000
P40261	NNMT	29.6	Nicotinamide N-methyltransferase OS	3	1	0.1320	0.0000
Q9Y6V0	PCLO	560.<br>4	Protein piccolo OS	0	1	0.1240	0.0000
Q8IVD9	NUDCD3	40.8	NudC domain-containing protein 3 OS	13	3	0.0910	0.0000
Q6PGP7	TTC37	175.<br>4	Tetratricopeptide repeat protein 37 OS	1	1	0.0670	0.0000
A0A075B<br>6R3	HELZ	64.2	Probable helicase with zinc finger domain OS	1	1	0.0570	0.0000
Q9UP95	SLC12A4	120.<br>6	Solute carrier family 12 member 4 OS	1	1	0.0370	0.0000
H0Y6I0	GOLGA4	246.<br>5	Golgin subfamily A member 4 (Fragment) OS	1	2	0.0180	0.0000
O95155	UBE4B	146.<br>1	Ubiquitin conjugation factor E4 B OS	3	2	0.0140	0.0000
E9PSA5	ALKBH3	25.9	Alpha-ketoglutarate-dependent dioxygenase alkB homolog 3 (Fragment) OS	6	1	0.0100	0.0000
Q04118	PRB3	31	Basic salivary proline-rich protein 3 OS	7	1	0.0100	0.0000

Q9ULT8	HECTD1	289.2	E3 ubiquitin-protein ligase HECTD1 OS	1	1	0.0100	0.0000
Q96KP1	EXOC2	104	Exocyst complex component 2 OS	1	1	0.0100	0.0000
Q53EP0	FNDC3B	132.8	Fibronectin type III domain-containing protein 3B OS	2	1	0.0100	0.0000
P11217	PYGM	97	Glycogen phosphorylase, muscle form OS	18	14	0.0100	0.0000
Q01546	KRT76	65.8	Keratin, type II cytoskeletal 2 oral OS	10	7	0.0100	0.0000
Q8N3R9	MPP5	77.2	MAGUK p55 subfamily member 5 OS	2	1	0.0100	0.0000
V5IRT4	UQCC2	14.8	Mitochondrial nucleoid factor 1 (Fragment) OS	6	1	0.0100	0.0000
P20929	NEB	772.4	Nebulin OS	0	1	0.0100	0.0000
M0R3A4	PIH1D1	20.8	PIH1 domain-containing protein 1 (Fragment) OS	9	1	0.0100	0.0000
M0QZH0	RCN3	19.8	Reticulocalbin-3 (Fragment) OS	8	1	0.0100	0.0000
A0A087WV42	PRH1	19.1	Salivary acidic proline-rich phosphoprotein 1/2 OS	24	1	0.0100	0.0000
F5H8G1	STARD10	21.9	START domain-containing protein 10 (Fragment) OS	7	1	0.0100	0.0000
K4DIA7	CD151	25.6	Tetraspanin (Fragment) OS	4	1	0.0100	0.0000
Q15750	TAB1	54.6	TGF-beta-activated kinase 1 and MAP3K7-binding protein 1 OS	9	2	0.0100	0.0000
P11172	UMPS	52.2	Uridine 5'-monophosphate synthase OS	3	1	0.0100	0.0000

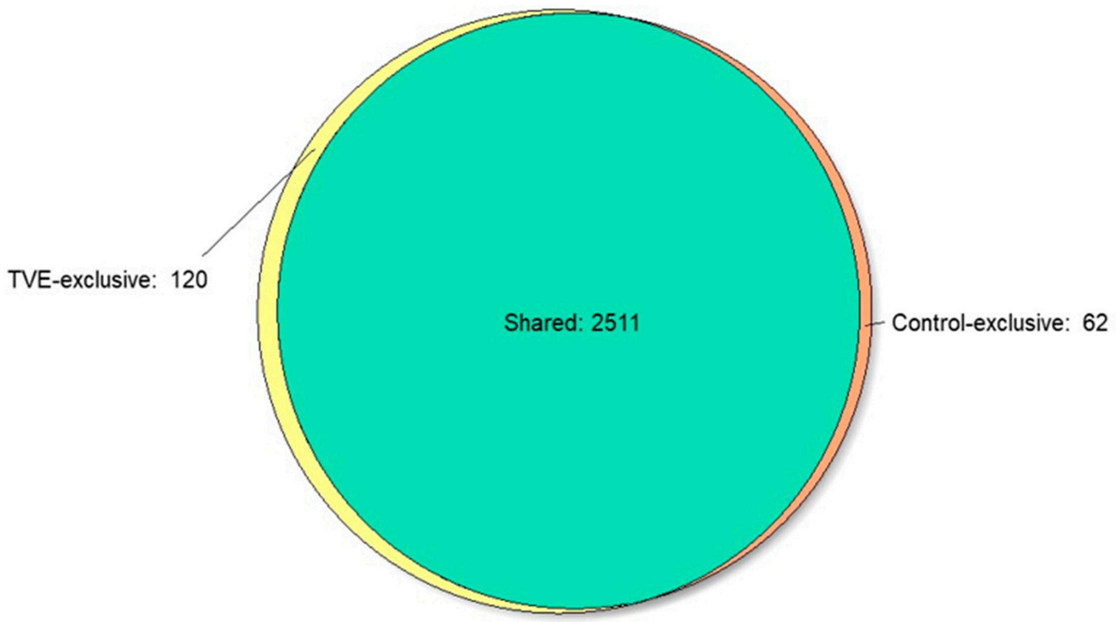

Figure S1: Venn diagram of unique and common proteins identified in HaCat cell secretomes treated or not with TVE.

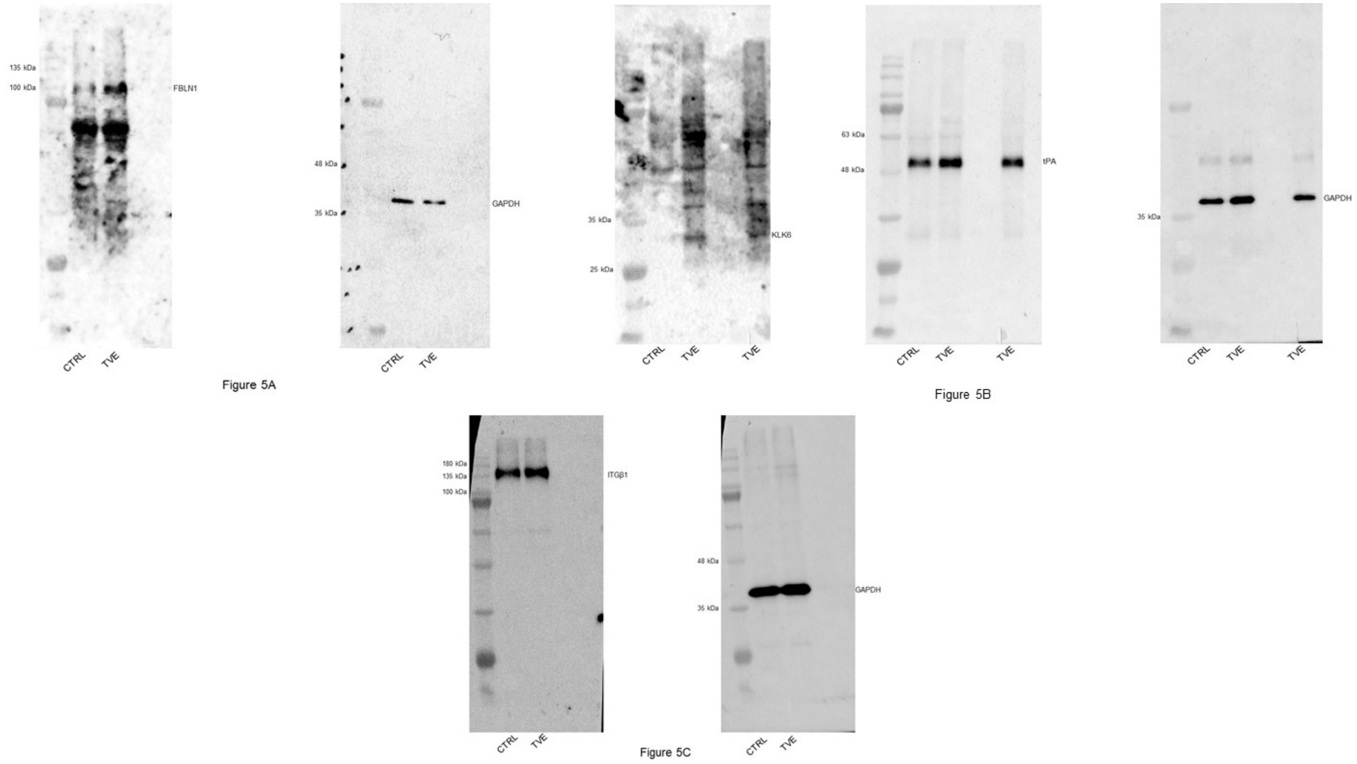

Figure S2: Uncropped western blotting analysis reported in Figure 5.
